# Engineering of Glioblastoma‐Derived Biomimetic Vesicles and Their Structural and Molecular Features

**DOI:** 10.1002/adhm.202503775

**Published:** 2026-05-08

**Authors:** Noelia Hernández‐Lobato, Hanan Abumanhal‐Masarweh, Martí de Cabo, Pablo Guerra, Marilena Hadjidemetriou, Neus Lozano, Kostas Kostarelos

**Affiliations:** ^1^ Nanomedicine Lab Catalan Institute of Nanoscience and Nanotechnology (ICN2) CSIC and BIST Barcelona Spain; ^2^ Departament de Química Facultat de Ciències Universitat Autònoma de Barcelona Bellaterra Spain; ^3^ NanoOmics Lab School of Biological Sciences and Manchester Cancer Research Centre The University of Manchester Manchester UK; ^4^ Centre For Nanotechnology in Medicine Faculty of Biology, Medicine & Health The University of Manchester Manchester UK; ^5^ Servei De Microscòpia i Difracció De Raigs X Universitat Autònoma De Barcelona Bellaterra Barcelona Spain; ^6^ Cryo‐Electron Microscopy Platform Molecular Biology Institute of Barcelona (IBMB‐CSIC) Joint Electron Microscopy Center at ALBA (JEMCA) Barcelona Spain; ^7^ Institute of Neuroscience Universitat Autònoma de Barcelona Barcelona Spain; ^8^ Institució Catalana de Recerca i Estudis Avançats (ICREA) Barcelona Spain

**Keywords:** cell‐derived, cryo‐EM, glioblastoma, liposomes, proteomics

## Abstract

Biomimetic nanosystems and vesicles have arisen as a novel approach to design vesicular transport systems with diverse therapeutic potential. The ‘biomimetic’ strategy involves the integration of cell membrane components into lipid bilayers, conferring them with biological properties originating from the cell of origin. Until now, most studies have primarily focused on the evaluation of the biological activity and function of different biomimetic nanosystems with limited exploration of the engineering parameters selected and little characterization of their features at the molecular level. This study aimed to address this knowledge gap by describing a preparation method for biomimetic lipid vesicles using traditional liposome fabrication principles and cellular components exclusively derived from glioblastoma (GL261) cell membrane proteins. Critical engineering parameters were studied, such as bilayer lipid and cholesterol content, the degree of surface PEGylation and some processing aspects like purification and quantification. Following fabrication, the GL261‐derived vesicles underwent purification using size exclusion chromatography to separate unbound proteins from the vesicles. Subsequently, the GL261‐derived vesicles were characterized by cryo‐EM and differential scanning calorimetry (DSC) to assess their morphological and thermal properties, respectively. Both cholesterol and PEGylated lipid content played an important role on the structural and colloidal features of the biomimetic vesicles (BV). Mass spectroscopy (LC‐MS/MS) revealed the proteomic signature of the fabricated vesicles at the molecular level. Collectively, these findings advance the rational engineering of BV and offer an in‐depth proteomic framework that reveals their molecular identity and functional potential. By connecting the design principles of fabrication with the molecular features of the vesicles, this study paves the way for next‐generation biomimetic platforms for cancer chemotherapy, immunomodulation and cancer vaccination.

## Graphical abstract

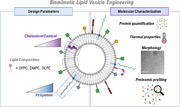

## 1 Introduction

Nanoparticle (NP) drug delivery systems are promising tools in nanomedicine, demonstrating remarkable clinical benefit in recent years. One of the strategies that has emerged over the last years is the incorporation of cellular components into these nanoparticles, mainly derived from the cell membrane, generating hybrid vesicle systems with some biological properties deriving from the cell of origin [1]. These ‘biomimetic’ systems attempt to address many of the challenges faced by more traditional drug delivery strategies, such as providing long blood circulation and stealth properties [2], offering specific targeting to sites of interest [3, 4] and generating specific immune responses [5]. More importantly, such approaches can also pave the way to the development of personalized nanomedicines featuring specific molecular components from each patient.

Under the umbrella of ´biomimetic´ systems, a considerable number of different nanocarrier designs can be found, that can generally be classified in two categories: cell‐membrane wrapped nanoparticles [4, 6] and biomimetic vesicles (BV) [7]. Cell‐membrane wrapped nanoparticles are composed of a synthetic, usually solid or semi‐solid (e.g. polymeric), NP core covered by a membrane bilayer that includes cell‐derived membrane components. These systems are typically generated by a top‐down approach, where the deconstructed cell membrane components are allowed to re‐assemble onto nanoparticles by extrusion or sonication methods. Alternatively, BV consist primarily of either: (i) the whole cell membrane components (in some cases with additional inclusion of synthetic lipids), termed as (cell) ´ghosts´ [8, 9]; or, (ii) of synthetic lipids as the main lipid component, with incorporation of a selected or random mix of protein extracts from the cells of origin [10, 11]. The strategy of incorporating selected protein cell components can theoretically offer some advantages in comparison to the incorporation of the whole cell membrane extract or the whole cell‐lysates, mainly in minimizing the complexity of the components to be incorporated, and offering a more controlled system, hence facilitating more accurate and potentially a more industrially translatable technology. Recent studies using BV have reported various therapeutic applications, such as anti‐inflammation [12], chemotherapy [13, 14, 15, 16], gene therapy [17], targeted drug delivery [18, 19, 20], and immunotherapy [21, 22, 23, 24].

With regards to the preparation of BV, most published works rely on traditional liposome fabrication techniques, such as lipid film hydration with an aqueous solution of the biological source followed by extrusion. There are relatively few investigations using alternative fabrication techniques such as ethanol injection [25] or microfluidics [26, 27, 28]. Although lipid film hydration is one of the most established techniques for BV preparation, systematic studies investigating the influence of key parameters, such as lipid bilayer composition, surface functionalization, and related physicochemical factors, remain scarce [10, 29, 30]. Consequently, there is an urgent need to identify and optimize the key parameters that influence the fabrication of BV, ensuring their long‐term stability, reproducibility, and functionality. Development of standardized protocols will be critical to advance these systems toward clinical translation, ultimately unlock their full potential for therapeutic applications.

Moreover, the majority of studies regarding BV focus on their functional evaluation, rather than in their methodological optimization and characterization, typically focused on measurements of hydrodynamic radius and surface charge, as well as basic morphological evaluation. The lack of characterization is especially noticeable in the proteomic analysis of the BV undertaken. From the few studies that provide proteomic characterization, the vast majority only perform qualitative evaluation by SDS‐PAGE and western blot, frequently to determine if certain proteins of interest present in the source have been incorporated in the resulting biomimetic systems. From the few studies that do execute proteomics analysis by LC‐MS/MS, most focused on describing the proteins present in the cell sources used (and not those that are part of the final BV) [31, 32, 33]. We consider proteomic profiling of any biomimetic system crucial, and a determinant factor of their downstream pharmacological and biological performance.

One of the potential uses of BVs proposed is as a novel delivery system of therapeutics in the treatment of glioblastoma multiforme (GBM). GBM is a grade IV malignant brain tumor constituted by a genetically unstable and heterogeneous population of cells, comprising tumoral cells, reactive astrocytes, and cancer stem cells [34, 35], typically resistant to chemotherapy, which are highly infiltrative. In addition, GBM is characterized by a high cell mitotic activity and vascularization [36] that lead to fast progression of the disease resulting in an aggressive and invasive phenotype. To date, multiple strategies have been attempted to improve its poor prognosis. The vast majority of those have been unsuccessful attempts to develop efficient targeted therapeutics, and biomimetic nanosystems have lately been considered in that context [37]. Until now, the preparation of GBM cell‐derived biomimetics has been little explored. Most studies have focused on utilizing GBM‐derived systems to exploit their inherent homotypic targeting of homologous tumors. For example, Jia et al. used glioma cell membrane proteins to integrate with liposomal doxorubicin, and reported their enhanced diffusion and penetration into GBM tissues [38]. Similarly, Li et al. engineered plasma membrane BV derived from RG2 cells co‐encapsulating the chemotherapeutics elemene and cabazitaxel [39], while more recently Xu et al. fabricated U251MG cell membrane BVs carrying temozolomide and a proteolysis‐targeting chimera (PROTAC) BRD4 inhibitor to overcome GBM chemoresistance [40]. Also, Arduino et al. also reported U87MG cell membrane–derived vesicles co‐loaded with paclitaxel and carboplatin using an in‐house 3D‐printed microfluidic chip [41]. In all these studies, the BV exhibited strong homotypic targeting toward GBM cells, enhanced blood–brain barrier (BBB) permeability in vitro and significantly suppressed tumor growth in vivo compared to conventional liposomal formulations. Alternative approaches have explored the combination of membranes from multiple cell types to endow hybrid vesicles with complementary biological functions. For instance, Liu et al. fabricated hybrid cell membrane–coated, ICG‐loaded liposomes by combining B16F10 (melanoma) and G422 (glioblastoma) membranes, achieving improved BBB penetration and tumour‐specific accumulation for enhanced image‐guided glioma resection [42]. Likewise, Zhou et al. developed a hybrid macrophage (RAW.264) and GBM (U87) plasma cell membrane biomimetic vesicular system capable of efficiently crossing the BBB and delivering paclitaxel to GBM cells [43].

In this study, we sought to address the limited understanding of how engineering variables influence the structural, biophysical, and molecular characteristics of BV. Using a glioblastoma‐derived membrane protein source (GL261), which constitutes one of the most widely used preclinical orthotopic GBM models, we systematically examined key fabrication parameters, including lipid and cholesterol ratios, PEGylation degree, and purification conditions, and evaluated their impact on vesicle integrity and composition. We further coupled these engineering assessments with detailed morphological, thermal, and proteomic characterization.

## 2 Materials and Methods

### 2.1 Membrane Protein Extraction

GL261 cells were seeded at a density of approximately 15 000 cells/cm^2^ and cultured in Roswell Park Memorial Institute (RPMI‐1640) medium supplemented with 10% fetal bovine serum (FBS) and 1% penicillin‐streptomycin solution. Cultures were maintained at 37°C in a humidified incubator with 5% CO_2_ and 95% of relative humidity. After reaching a confluence of around 90%, cells were expanded by removing old media, washed twice with phosphate saline buffer (PBS) without calcium and magnesium, trypsinized, and replated in new flasks with fresh media. Cells expanded between passages 14 to 18 were used for the preparation of membrane protein extracts. All culture media, buffers, and supplements were purchased from Sigma–Aldrich (Merck, Spain).

Protein extracts were produced using ProteoExtract Native Membrane Protein Extraction Kit purchased from Sigma‐Aldrich (Merck, Spain, Cat. No. 444810). Briefly, after reaching a GL261 cell confluence of approximately 90% in a T75 flask, the old media was removed and cells were washed twice with 5 mL of Washing Buffer. Then, 6 mL of Extraction Buffer I and 30 µL of Protease Inhibitor were added, and cells were incubated for 10 min at 4°C and 0.5 g. The cell suspension was centrifuged for 10 min at 4°C and 9 000 g. The supernatant was discarded, and the cell pellet was resuspended in 3 mL of Extraction Buffer II and 30 µL of protease inhibitor by pipetting multiple times up and down. The resulting cell suspension was incubated for 30 min at 4°C and 0.5 g and centrifuged for 10 min at 4°C and 9 000 g. The pellet was discarded and the supernatant contained the membrane protein extract, which was quantified by fluorescamine assay.

### 2.2 Liposomes and BV Preparation

Liposomes and glioblastoma BV were prepared using 1,2‐dipalmitoyl‐sn‐glycero‐3‐phosphocholine (DPPC, Cat. No. 850355P), 1,2‐dimyristoyl‐sn‐glycero‐3‐phosphocholine (DMPC, Cat. No. 850345P) and 1,2‐dilauroyl‐sn‐glycero‐3‐phosphocholine (DLPC, Cat. No. 850335P) purchased from Avanti Polar Lipids, cholesterol purchased from Sigma‐Aldrich (Merck, Spain, Cat. No. C3045) and 1,2‐distearoyl‐sn‐glycero‐3‐phosphoethanolamine‐N‐[carboxy(polyethylene glycol)‐1000] (sodium salt) (DSPE‐PEG_2000_, Cat. No. 588200) purchased from Lipoid GmbH. Several compositions were investigated by using DPPC, DMPC, and DLPC as primary phospholipids and varying the percentage of cholesterol (0%, 15%, and 40%), as well as the degree of PEGylation (0%, 1%, and 5%) in the vesicles. Liposomes and BV were produced by standard liposome preparation techniques, such as lipid film hydration and extrusion. Briefly, 20 µmol of lipid (with the appropriate amount of phospholipid (DPPC, DMPC, DLPC), cholesterol and DPPC:DSPE‐PEG_2000_) were dissolved in chloroform:methanol (4:1 v/v) and the solvent was evaporated to complete dryness in a rotary evaporator equipped with a water bath at 45°C. For liposomes, the lipid film was hydrated only with 2 mL of PBS, meanwhile for BV, it was hydrated with 2 mL of protein extract at 300 µg/mL in PBS. In both cases, the mixture was incubated in a water bath at 45°C for 1 h. The mixture was vortexed for a few seconds until full resuspension of the lipid film and extruded five times at 45°C with a LIPEX liposome extruder (Evonik, Germany) equipped with polycarbonate membranes with porous size of 800, 400, and 200 nm.

### 2.3 Dynamic Light Scattering (DLS) and Surface Charge

The hydrodynamic size, polydispersity index and ζ‐potential of GL261‐derived vesicles and liposomes were measured using a Zeta‐sizer Nano ZS (Malvern Panalytical, United Kingdom) using disposable capillary cells by diluting 10 µL of sample in 1 mL of samples in deionized water.

### 2.4 Biomimetic Vesicle Purification

The purification of the GL261 BV was performed by size exclusion chromatography. The efficiency of separation between the BV and the unbound proteins was evaluated using two solid phases: Sepharose CL‐4B (Cat. No. CL4B200) and Sephadex G25 (Cat. No. G2580), both purchased from Sigma–Aldrich. Econo‐Pac Chromatography Columns (Biorad, Spain) were filled with 10 cm of previously hydrated size exclusion chromatography solid phase and washed three times with saline buffer prior use. BV were then introduced in the size exclusion chromatography column and 1 mL fractions were collected. Fraction eluted after introducing BV inside the column was considered as fraction F0. The lipid content in each of the fractions was analyzed by Stewart assay whereas the protein content was analyzed by fluorescamine assay. After purification, liposomes and BV were concentrated using Vivaspin 6 (Sartorius) at 9,000 g and 4°C until reaching around 200 µL for protein quantification and proteomics analysis.

### 2.5 Lipid Content Quantification by STEWART assay

The lipid content in the vesicles was evaluated by the Stewart assay. In a 1.5 mL Eppendorf tube, 400 µL of Stewart Reagent (0.1 M FeCl_3_ and 0.4 M NH_4_SCN in water, both purchased from Sigma–Aldrich, Cat. No. 236489 and 221988) was added, together with between 5 and 20 µL of vesicles and 1 mL of chloroform. The biphasic mixture was vortexed for 20 s and centrifuged for 5 min at 20°C and 3 000 g. The organic phase is transferred to a clean Eppendorf tube, and its absorbance was recorded at 470 nm. The absorbance was interpolated in a calibration curve prepared with the same lipid formulation as the sample to obtain the concentration of the sample.

### 2.6 Protein Content Quantification by Fluorescamine Assay

Fluorescamine assay was used for the quantification of the protein content in the membrane protein extracts and the vesicle samples. Briefly, 150 µL of sample in PBS was added into a black 96 well fluorescence plate. Then, 50 µL of a 3 mg/mL solution of fluorescamine (ThermoScientific, Cat. No. 43749) in DMSO was added to each well and samples were homogenized by mild shaking for 10 s. The fluorescence intensity was measured in the plate reader SpectraMax iD3 at ICN2 Nanobioelectronics and Biosensors Group, using excitation and emission wavelengths of 400 and 460 nm, respectively. The fluorescence signal was then interpolated onto a bovine serum albumin calibration curve (BSA, ThermoScientific, Cat. No. 23210B), ranging from 10 to 200 µg/mL.

### 2.7 Cryo‐Transmission Electron Microscopy

The native morphology of the BV was studied by cryo‐transmission electron microscopy (cryoTEM) at the Joint Electron Microscopy Center at ALBA (JEMCA). Samples were vitrified by depositing 3.0 µL of vesicles in a previously glow‐discharged grid (Micro to Nano, EMR Lacey Carbon support film on copper 400 square mesh, Cat. No. 22‐1MLC40), blotted for 4 s, and plunged into liquid ethane at −186°C (Vitrobot cryo‐plunger, Thermo Scientific). Samples were images in Glacios 200 kV transmission electron microscope (ThermoScientific) with extreme field emission gun (X‐FEG) optics, equipped with a cryogenic sample manipulator robot and Falcon 4 direct electron detector. The images were taken using an applied defocus of ‐2.00 µm, an exposition time of 6 s and a dose of 4263.85e/nm^2^.

### 2.8 Differential Scanning Calorimetry

The thermal properties of the BV and liposomes were analyzed by Differential Scanning Calorimetry (DSC) at the Thermal Analysis and Calorimetry Service from the Institute for Advanced Chemistry of Catalonia (IQAC‐CSIC). Briefly, 100 µL of sample were placed in an aluminum sample holder and hermetically sealed. Then, samples were placed in a DSC821 calorimeter (Metler Toledo, New Zealand) together with an empty crucible as a reference and underwent controlled heating from 0°C to 70°C at 5°C/min, meanwhile monitoring the heat flow.

### 2.9 Protein Analysis by SDS‐PAGE

The proteins incorporated in the vesicles were visualized by SDS‐PAGE. The preparation of the sample consisted in mixing 20 µL of sample with 16 µL of Novex Tris‐Glycine SDS Running Buffer (2X) (Invitrogen, USA, Cat. No LC2676) and 4 µL NuPAGE Sample Reducing Agent (10X) (Invitrogen, USA, Cat. No. NP0004). Samples were vortexed and incubated at 95°C for 5 min. After cooling down, samples were added to Novex Tris‐Glycine Mini Protein Gels, 4–20%, 1.0 mm, WedgeWell, using Thermo Scientific PageRuler Prestained Protein Ladder (10 to 180 kDa, Cat. No. 26619) as a reference. Gels were run in Novex Tris‐Glycine SDS Running Buffer (Cat. No. LC2675) for 40 min at 225 V, stained for 3 h with Thermo Scientific Imperial Protein Stain (Cat. No. 24615) and washed several times with ultrapure water.

### 2.10 Protein Analysis by Mass Spectrometry

Proteins contained in membrane protein extracts and BV were digested using S‐Trap micro columns, as published before by our group [44, 45]. Briefly, samples containing 3 µg of protein were mixed with lysis buffer containing 5% SDS in triethylammonium bicarbonate buffer (TEAB, 50 mm, pH 7.5; Sigma–Aldrich, Cat. No. T7408) to enable protein denaturation prior reduction with dithiothreitol (5 mm, ThermoScientific, Cat. No. BP172) and alkylated iodoacetamide (15 mM; Sigma–Aldrich, Cat. No. I1149). Unreacted iodoacetamide was quenched with dithiothreitol (5 mm). Samples were centrifuged (14 000 g, 10 min, 4°C) for the collection of protein lysates present in the supernatant. Protein lysates were acidized with phosphoric acid (12%) and mixed with six‐volume equivalents of binding buffer (100 mm TEAB in 90% methanol, pH 7.1). Proteins were immobilized in S‐Trap micro columns (ProtiFi, LLC, USA) by centrifugation (4 000 g, 2 min, 4°C), washed with methyl *tert*‐butyl ether: methanol (1:3) and washed four times more with binding buffer. Immobilized proteins were then digested with trypsin (0.1 µg/mL, 1 h, 47°C). Resultant peptides were eluted from S‐Trap columns using digestion buffer (50 mM TEAB), 0.1% formic acid in water and 30% acetonitrile in water containing 0.1% formic acid. Peptide samples were desalted using Oligo R3 beads (Life Technologies, Cat. No 1133903) stored in 50% acetonitrile in water with 0.1% formic acid in 30% acetonitrile and dried in a Speedvac Savant (Thermo Scientific).

Digested samples were analyzed by liquid chromatography mass spectrometry/mass spectrometry (LC‐MS/MS). The separation was performed on a Thermo RSLC system consisting of a NCP3200RS nano pump, WPS3000TPS autosampler, TCC3000RS column oven and analytical column (Waters nanoEase M/Z Peptide CSH C18 Column, 130Å, 1.7 µm, 75 µm X 250 mm) configured with buffer A as 0.1% formic acid in water and buffer B as 0.1% formic acid in acetonitrile. A multistage gradient was used varying the percentage of buffer B, as described: 1% to 6% B over 2 min, 6% to 18% over 44 min, 18% to 29% over 7 min, and 29% to 65% over 1 min before washing for 4 min at 65%. Then, the multistage gradient was dropped down to 2% B in 1 min. The analytical column was connected to a Thermo Exploris 480 mass spectrometry system via a Thermo nanospray Flex Ion. Fragmentation data was obtained from signals with a charge state of +2 or +3 and an intensity over 5 000, and they were dynamically excluded from further analysis for a period of 15 s after a single acquisition within a 10 ppm window.

### 2.11 Data Analysis and Statistics of MS data

For the statistical comparison of the proteins identified in the GL261‐derived membrane protein extracts and BV was performed using Mascot Software and Scaffold, as previously described by our group [46]. Briefly, acquired mass spectrometry data was matched to SwissProt_2016_04 database for mouse taxonomy using Mascot (Matrix Science UK). Relative protein quantification was performed based on spectral counting using Scaffold software (version 4.3.2, Proteome Software, Inc.). Protein identification were considered valid when their probability was higher than 99.0% and contained at least two identified peptides.

### 2.12 Data and Statistical Analysis

Unless specified, all data processing and plots generation were performed using OriginPro (version b9.5.0.193) software. Statistical significance of data presented as mean ± standard deviation (SD) was assessed using one‐way ANOVA followed by Bonferroni post hoc correction, with *p* < 0.05 considered statistically significant.

## 3 Results and Discussion

### 3.1 Engineering of GL261 BV

**FIGURE 1:**
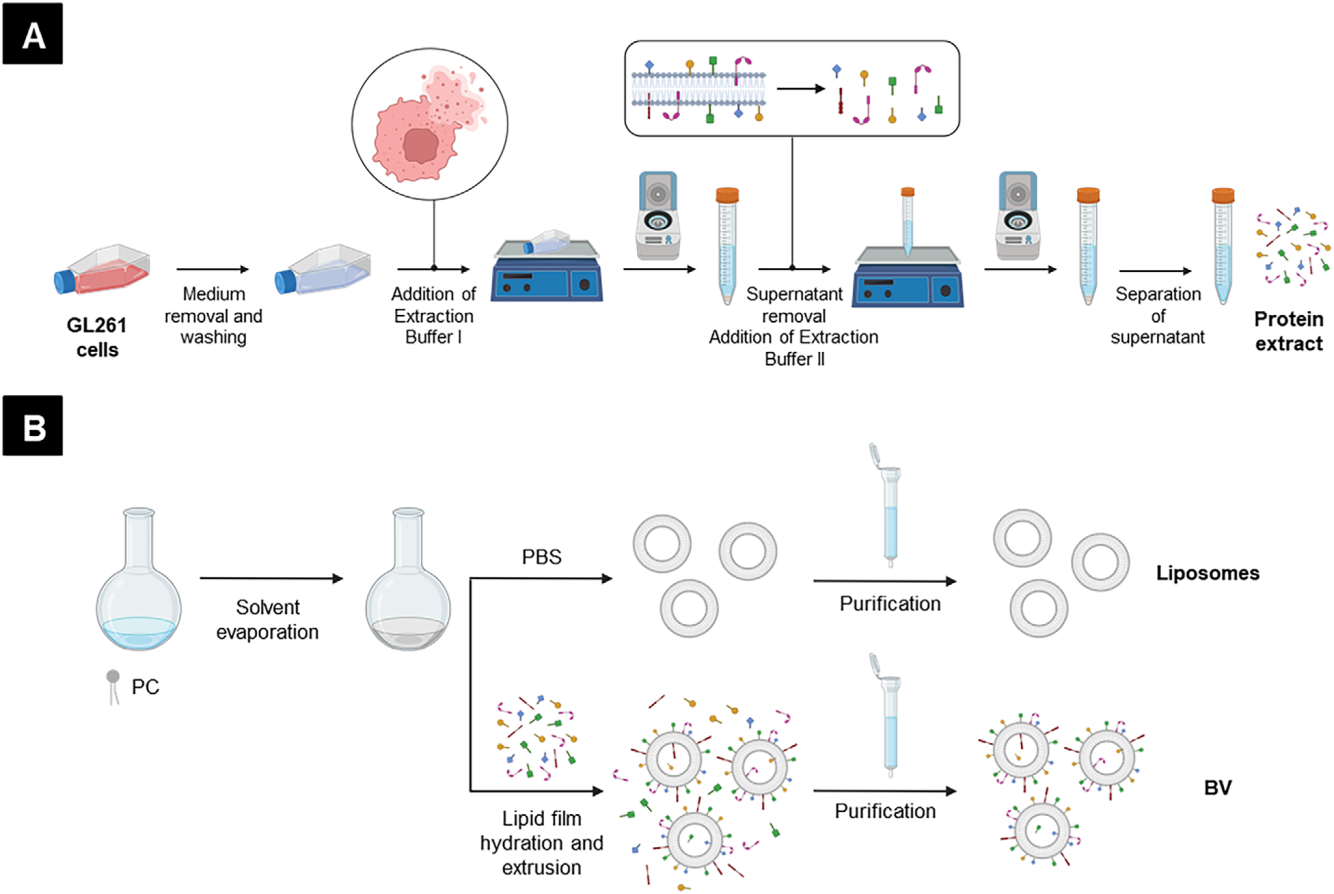
Graphical representation of the methodologies used for the preparation of the GL261 BV. (A) The extraction of GL261 membrane proteins with ProteoExtract Native Membrane Kit. (B) Preparation of liposomes and BV based on lipid film hydration and extrusion. Liposomes and BV were purified using size exclusion chromatography columns (see Figures S5 and S6).

Glioblastoma BV were composed of a synthetic lipid core incorporating proteins extracted from GL261 immortalized murine cell line. Proteins were extracted from cultured GL261 cells using the ProteoExtract Native Membrane Protein Extraction Kit. This kit was specifically chosen among the variety commercially available because it was reported to provide the lowest contamination of cytosolic proteins in the protein extract [47]. Briefly, as it is schematized in Figure 1A, the preparation of the protein extract relied on two sequential steps. First, Buffer I was used to lyse GL261 cells and then, the protein extract was obtained after the incubation of the cell suspension with Buffer II. The protein concentration of the extract was then quantified using fluorescamine assay. The methodology and conditions followed are detailed in Figure S1A. The concentration of the protein extract was obtained by correlating the sample's fluorescence intensity to a calibration curve built with BSA (Figure S1B) and was determined to be 1553 ± 135 µg/mL (*n* = 3), as shown in Figure S1C. The sensitivity of the fluorescamine assay was also studied by evaluating the signal to noise ratio of increasing BSA concentrations (Figure S1D). In here, we observed that for concentrations of BSA lower than 10 µg/mL, the signal to noise ratio was lower than 3 and the variability of the sample was around 30%. Therefore, fluorescence signals corresponding to BSA concentration values lower than 10 µg/mL could not be accurately measured and were considered as not quantifiable in all the protein content quantifications conducted in this study.

BV were fabricated using conventional liposome fabrication techniques, such as lipid film hydration and extrusion, followed by a purification step (Figure 1B). The protein extract obtained from GL261 cells was incorporated during the hydration step to form BV. Liposomes were prepared as a negative control without the incorporation of protein by hydrating the lipid film with PBS. It is worth highlighting that the preparation method used in this study does not provide selective directionality of proteins inside the vesicle. Therefore, proteins embedded in the membrane could be potentially oriented toward the outside environment of the vesicle or toward the inner core. Additionally, given the probable presence of soluble proteins, these could also be localized inside the vesicles’ aqueous inner core or adsorbed on the vesicle surface forming a corona. This has not been a matter of investigation during this study but could potentially be studied in the future and would have a direct impact on the therapeutic application of the vesicles. Further investigations in this direction could involve protein labelling or immunogold cryo‐electron microscopy [48, 49]. Given the situation where these BV would be used as targeted carriers, the proteins incorporated would be required to hold directionality toward the outer environment. Nonetheless, some other applications, such as immunotherapy, would only require the proteins to be contained inside the vesicles, independently of their directionality. The molecular design of the BV was based on using phosphatidylcholines (PC) as a backbone, since these are the major components of cell membrane lipid bilayers, and further optimized according to two parameters: (i) the modulation of the lipid bilayer rigidity by evaluating different cholesterol concentrations, and the number of carbons in the phospholipid alkyl chains; and (ii) the modification of the vesicle surface hydrophilicity by altering degree of PEGylation, by changing the ratio of the DSPE‐PEG_2000_ lipid component.

### 3.2 Cholesterol Content

It is well reported in the literature that the incorporation of cholesterol into the liposomal bilayer affects intrinsic membrane properties such as membrane fluidity and permeability, the phase transition temperature (Tm), as well as the packing degree of phospholipids [50]. These properties have a direct impact on valuable characteristics from drug formulations, such as the stability over time and the drug incorporation efficiency. Similarly, in the present study it was hypothesized that the cholesterol content could potentially impact on the protein embedding into the BV.

**FIGURE 2:**
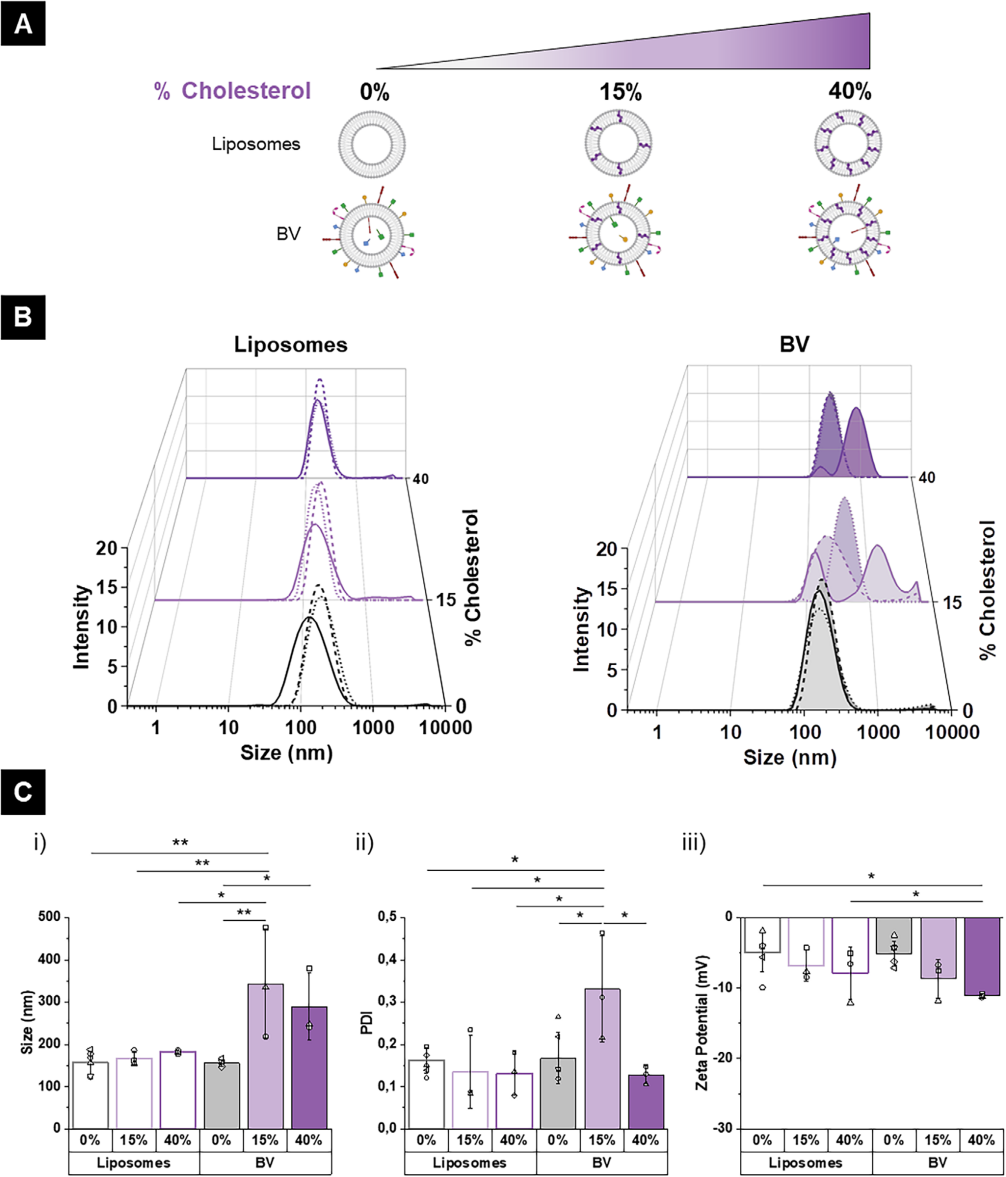
Engineering of BV by altering their cholesterol content. (A) Graphical representation of liposomes and BV prepared with different cholesterol content. (B) Colloidal stability evaluation by dynamic light scattering of liposomes and vesicles before purification with 0% (black lines), 15% (light purple lines), and 40% (dark purple lines) cholesterol content. For BV, filled curves are used. For each composition the size curves of *n* = 3 are shown. (C) Evaluation of i) size and ii) polydispersity index by dynamic light scattering and iii) zeta potential by electrophoretic mobility of liposomes (empty bars) and BV (filled bars) for 0% (black), 15% (light purple), and 40% (dark purple) cholesterol content. Data is represented as the mean ± standard deviation of *n* = 3. Statistical significance is indicated on the chart only for comparisons with p‐values < 0.05.

Most of the liposome formulations currently used in the clinic contain up to a 45% (mol/mol) of cholesterol in their composition [51]. Therefore, as shown in Figure 2A, liposomes and BV were prepared initially containing DPPC as the backbone phospholipid and the concentration of cholesterol was screened between 0%, 15%, and 40% cholesterol (mol/mol). After the extrusion with polycarbonate membranes (Figure 2B), no significant differences in the size distribution were identified in liposomes when analyzed by DLS, where samples exhibited highly monodisperse size distribution curves and maxima around 160 nm. Analysis by DLS after the extrusion of BV revealed that when cholesterol was added to the lipid bilayer, vesicles showed increased polydispersity and low reproducibility between replicates. Size distribution data was analyzed more deeply by considering mean hydrodynamic size (Figure 2C i) and polydispersity index (Figure 2C ii) values. With regards to liposomes, the size and polydispersity index were found to be below 200 nm and 0.20, respectively, for the three different concentrations of cholesterol studied. Nonetheless, for GL261 BV, samples containing 15% and 40% of cholesterol showed either higher sizes than 200 nm or polydispersity indexes higher than 0.20. This increased size and polydispersity were attributed to an increased rigidity of the membrane due to the incorporation of cholesterol and as a result, the production of BV with small, narrow, and monodisperse size distribution was hampered. Therefore, the vesicle system with optimal mean size and monodispersity was that composed of DPPC alone.

The surface charge of the liposomes and BV was also monitored by measuring the zeta potential by electrophoretic mobility (Figure 2C iii). In this, all formulations were showing slightly negative values ranging from −5 to −10 mV. No significant differences were found between liposomes and BV. Although surface charge has been reported previously as an indication for protein incorporation into BV, changes in the surface charge can be attributed to a variety of factors, such as protein charge, the amount of protein incorporated and their conformation and orientation. As a result, further studies are needed to confirm protein incorporation into the generated vesicles.

### 3.3 Lipid Composition

For comparative purposes, the same study was conducted using DMPC and DLPC as the main phospholipid component of the vesicles. Compared to DPPC, which contains two units of palmitic acid (16C) in its structure, DMPC contains two units of myristic acid (14C), and DLPC contains two units of lauric acid (12C). The number of carbon atoms in the fatty acids forming the phospholipid has a direct impact on the fluidity of the lipid bilayer, where shorter chains lead to more fluid membranes. As shown in Figure S2A,B, DMPC and DLPC exhibit similar patterns of behavior to DPPC, where the incorporation of cholesterol produces an increase in the hydrodynamic size and PDI after extrusion of BV when analyzed by DLS. In the case of liposomes alone (without any cell component incorporated), no significant differences were observed in size nor PDI after extrusion, which were always below 200 nm and 0.2, respectively, independently of the phospholipid and the cholesterol concentration used. A closer look at the PDI value of the formulations containing 0% cholesterol may suggest that DPPC shows more similar values between liposomes and BV. Contrarily, DMPC and DLPC show higher PDI and higher variability for BV compared to liposomes, overcoming the limit of 0.2.

With regards to surface charge, as shown in Figure S2C, no significant differences are observed between DPPC, DMPC and DLPC, which range from −2 to −10 mV. Moreover, the majority of BV show a slight decrease in the overall surface charge compared to liposomes, which would reinforce the hypothesis that proteins are present on the biomimetic vesicle surface, independently of the phospholipid used in the lipid formulation.

We concluded that among the different phospholipids studied, DPPC stood out as the most suitable option to continue our investigations on the fabrication of BV, as it was resulting in consistent mean hydrodynamic diameters below 200 nm and lower polydispersity compared to the other phospholipids.

### 3.4 The Effect of PEGylation on the Colloidal Stability of GBM‐Derived Vesicles

The incorporation of stealth components into vesicles is a widely used and advantageous strategy, especially in the case of polyethylene glycol (PEG). This is because PEGylation provides interesting benefits [52, 53], such as extended circulation times, as well as improved stability. The stabilization of the formulation is increased following two approaches: (i) by hindering the accessibility of degradative enzymes and (ii) by protecting the structural integrity of vesicles and increasing their shelf‐life.

**FIGURE 3:**
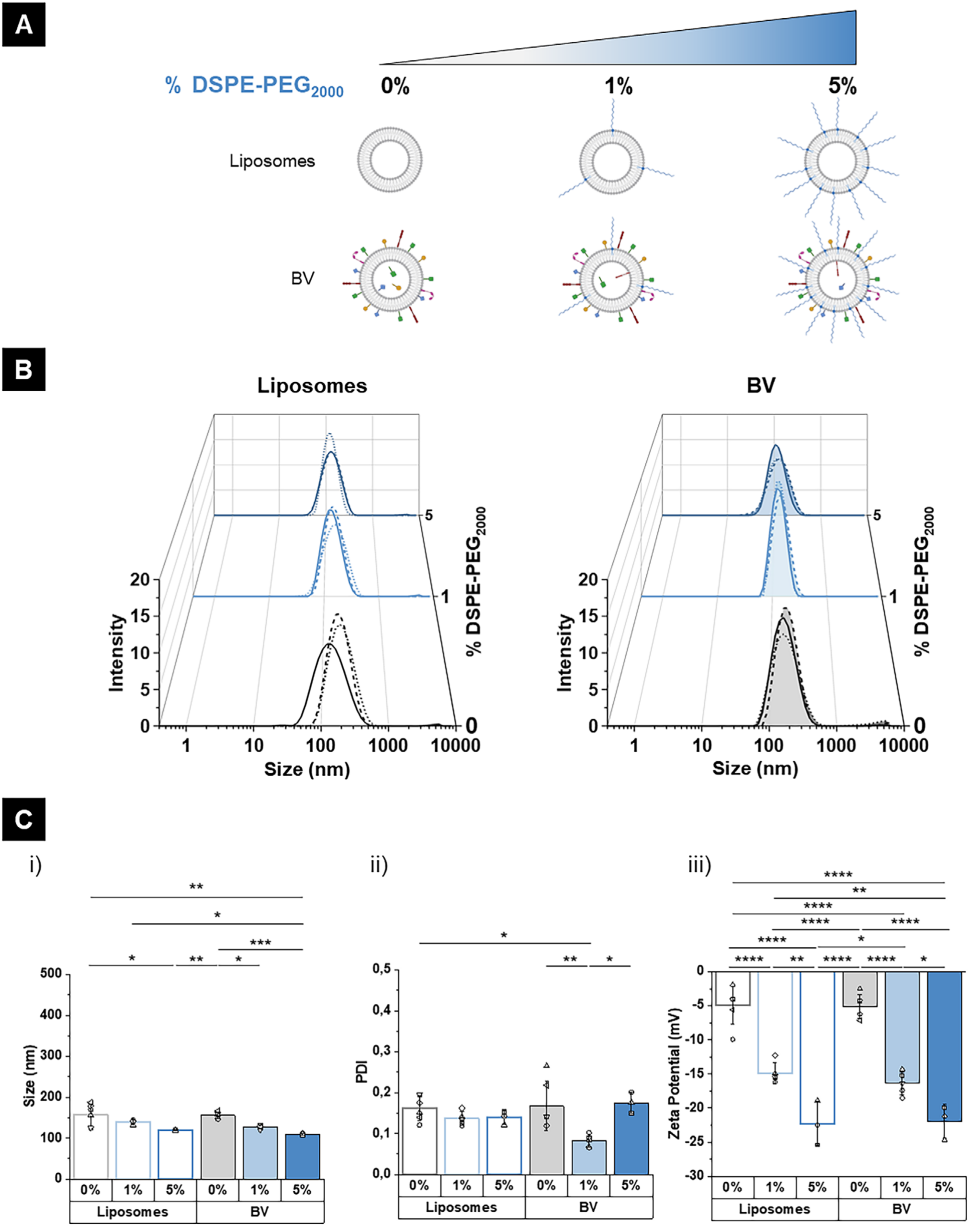
Engineering of BV by altering their degree of PEGylation. (A) Graphical representation of the liposomes and BV prepared with different DSPE‐PEG_2000_ content. (B) Colloidal stability evaluation by dynamic light scattering of liposomes and BV before purification composed of DPPC with 0% (black lines), 1% (light blue lines) and 5% (dark blue lines) DSPE‐PEG_2000_. For BV, filled curves are used. For each formulation, size curves of *n* = 3 are represented. (C) Evaluation of i) size and ii) polydispersity index by dynamic light scattering and iii) zeta potential by electrophoretic mobility of liposomes (empty bars) and BV (filled bars) for 0% (black), 1% (light blue), and 5% (dark blue) DSPE‐PEG_2000_ content. Data is represented as the mean ± standard deviation of at least *n* = 3. Statistical significance is indicated on the chart only for comparisons with p‐values < 0.05.

In this study, PEGylation was therefore contemplated as a stabilization strategy by turning the surface of the vesicles more hydrophilic. This was performed by incorporating different molar percentages of DSPE‐PEG_2000_ to the lipid composition while using DPPC as main phospholipid. In the liposome formulations currently available in the clinic [51], the concentration of PEG ranges from 5% to 10%. According to this and to the recently reported immunogenicity of PEG [54], the degree of PEGylation of liposomes and BV evaluated in this study ranged from incorporating from 1% to 5 of DSPE‐PEG_2000_ (mol/mol) to DPPC (Figure 3A).

Right after extrusion, the colloidal stability of the prepared liposomes and BV was monitored using DLS (Figure 3B). Liposomes showed narrow and reproducible size distribution curves with maxima around 160 nm for DPPC alone and around 120 nm for 1% and 5% DSPE‐PEG_2000_ formulations. This phenomenon was also observed in BV, which showed comparable size distributions to liposomes. The size distribution data for both liposomes and BV was evaluated more in detail through the analysis of the hydrodynamic size (Figure 3C i) and polydispersity index (Figure 3C ii). With regards to the hydrodynamic size, both liposomes and BV containing 0%, 1%, and 5% of DSPE‐PEG_2000_ displayed sizes lower than 200 nm, while showing a slight decrease in the mean value with increased concentration of DSPE‐PEG_2000_. In relation to the polydispersity index, all the formulations of liposomes and BV showed values below 0.20. Nonetheless, the formulation containing 1% of DSPE‐PEG_2000_ showed the lowest values, 0.14 ± 0.02 for the liposomes and 0.08 ± 0.02 for the BV. The surface charge of the prepared vesicles was also studied by monitoring the zeta potential measured by electrophoretic mobility (Figure 3C iii). No significant differences were found in the surface charge when comparing liposomes and BV, however the surface charge was observed to become more negative when the percentage of PEGylation increased. This event was attributed to the presence of the carboxylate groups at the distal end of the PEG chain in DSPE‐PEG_2000_.

In addition, the colloidal stability of DPPC:DSPE‐PEG_2000_ (1%) BV was evaluated studied while storing at 4°C (Figure S3). After 1 month, the size distribution and polydispersity index of liposomes remained unchanged. In the case of the BV, the size distribution maxima stayed steady around 150 nm. The polydispersity index was slightly increased from 0.09 to 0.15, even though it continued to fulfill the acceptance criteria, as it remained lower than 0.20. Hence, the addition of DSPE‐PEG_2000_ to the BV formulation contributed to narrow down hydrodynamic size and polydispersity, as well as helped stabilizing the formulation, which remained unchanged for one month. Consequently, DPPC:DSPE‐PEG_2000_ (1%) was the formulation selected to continue with the purification and characterization of the BV.

### 3.5 Purification of GBM‐Derived Vesicles by Size Exclusion Chromatography

While several methodologies have been applied for the purification of the unbound proteins from the BV, in this study we chose size exclusion chromatography for the purification of liposomes as it provides high selectivity for the separation according to the size of components present in a mixture, as well as the scalability and ease of use. We evaluated two different size exclusion chromatography solid phases that are highly used across the literature for the purification of liposomes, Sephadex G25 and Sepharose CL‐4B, in order to maximize the purification yield of the BV. With regards to the differences of both solid phases, Sephadex G25 is composed of cross‐linked dextran, has a fractionation range of 1–5 kDa and is typically used for buffer exchange and the removal of contaminants. On the contrary, Sepharose CL‐4B is composed of cross‐linked agarose, has a fractionation range of 60–20.000 kDa and is commonly used for the purification of big molecules as well as for coupling affinity ligands to the matrix.

The efficiency on the purification was evaluated by determining the yield and degree of separation between DPPC:DSPE‐PEG_2000_ (1%) liposomes and protein extract. The lipid content in each of the fractions was evaluated by using Stewart assay, where the absorbance of each of the samples was interpolated in a calibration curved built with known concentrations of the lipid mixture forming the vesicles (Figure S4). As it is shown in, the major part of the liposomes were collected in fractions F4 to F6, correlating with previous results obtained in our lab [55, 56]. A fraction of liposomes was also found in fraction F7; however, this was a minority compared to the content found in F4‐F6. These results were observed for both Sephadex G25 and Sepharose CL‐4B, nonetheless, the lipid content in F4‐F6 for Sepharose CL‐4B was higher than in the case of Sephadex G25, 90.0 ± 5.6% vs. 74.3 ± 19.1%, respectively. Consequently, the fractions selected for the obtention of purified BV were F4‐F6. With regards to the protein extract, the quantification in each of the fractions was performed using fluorescamine assay, where the fluorescence intensity of each of the samples was interpolated in a BSA calibration curve. In the case of Sephadex G25, the protein extract started eluting from the size exclusion chromatography column in F8, whereas for Sepharose CL‐4B it started eluting in F10. Therefore, considering the results obtained for liposomes and protein extract, both solid phases would be appropriate for a successful purification of BV when selecting F4‐F6, as both would provide a proper separation between the vesicles and the non‐incorporated proteins. BV made of DPPC:DSPE‐PEG_2000_ were purified as well using size exclusion chromatography (Figure S5) and their lipid and protein content was analyzed for both solid phases. No significant differences were found between Sephadex G25 and Sepharose CL‐4B in the quantification of neither the lipid content (6.8 ± 0.7 µmol vs. 6.3 ± 0.6 µmol, respectively) nor the protein content (29.8 ± 6.7 µg vs. 37.5 ± 12.8 µg, respectively), as displayed in Figure S6. The results obtained for the purification and quantification of BV reinforce the premise that both Sephadex G25 and Sepharose CL‐4B would be suitable for a proper purification. Nevertheless, Sepharose CL‐4B is the preferred solid phase to continue with the study, as it provides a bigger separation between purified BV and protein extract.

In our particular case, fluorescamine assay was the adopted method to measure the protein content over more standard methods in the literature, such as BCA assay. This is because BCA assay (the methodology followed is shown in Figure S7A) is a colorimetric method based on the measurement of absorbance and the presence of liposomes in the sample involves the scattering of the incident light, leading to a false signal of absorbance (Figure S7B). For the BCA assay, the protein concentration was calculated by interpolating the sample signal into a standard calibration curve built with BSA (Figure S7C).

### 3.6 Thermal and Morphological Characterization of GBM BV

**FIGURE 4:**
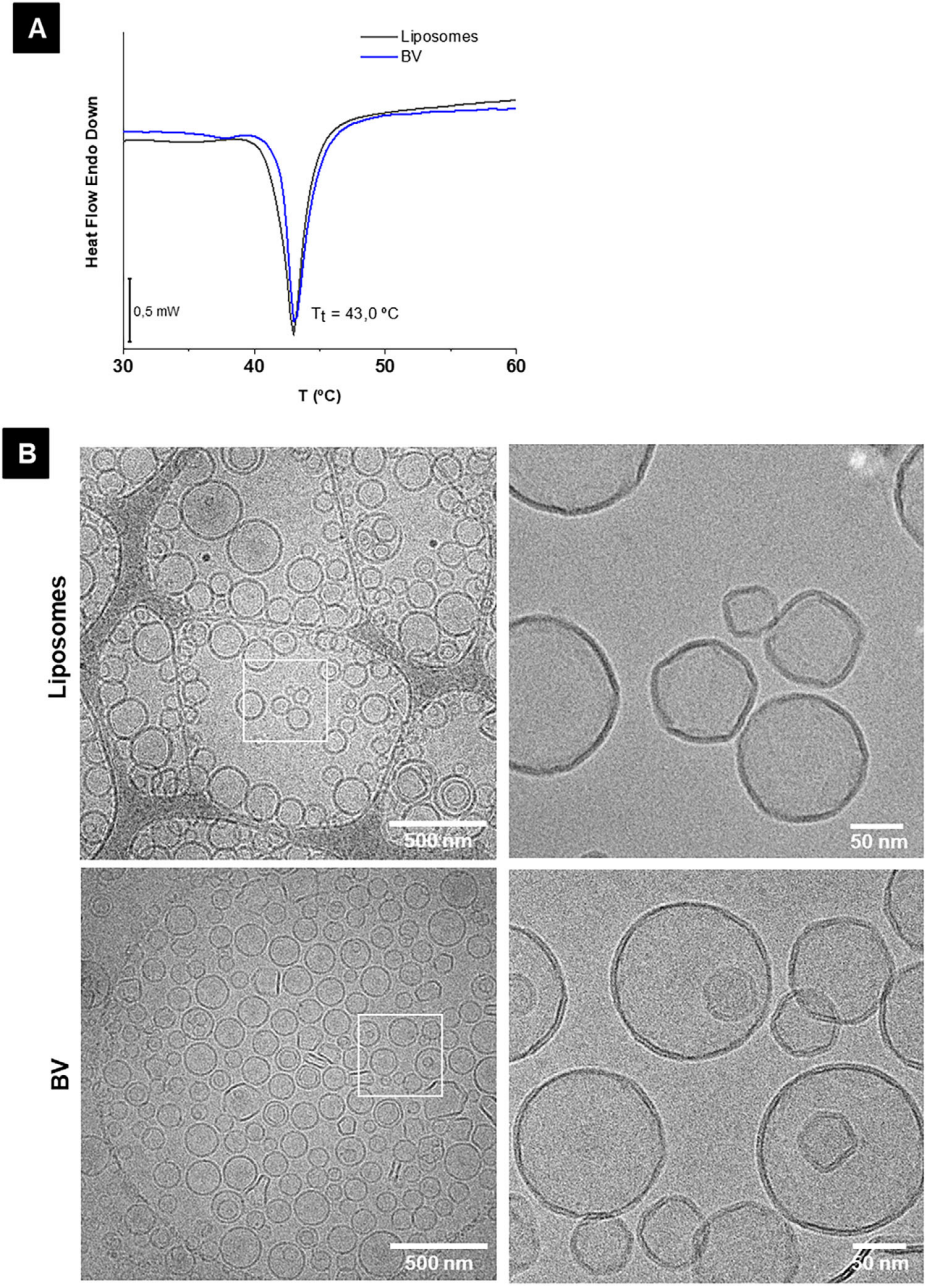
Thermal and morphological characterization of BV composed of DPPC:DSPE‐PEG_2000_ (1%) after purification. (A) DSC thermograms representing the heat flow (endothermic peaks down) versus the sample temperature of liposomes (black) and BV (blue). (B) Morphological evaluation of liposomes and BV by cryo‐EM.

After the purification of the vesicles, key physicochemical aspects were evaluated, including their thermal properties and their morphology. Their thermal properties are important to determine membrane fluidity, bilayer phase transition temperature and overall colloidal stability [57]. DPPC:DSPE‐PEG_2000_ (1%) vesicles prepared in this study were evaluated by DSC (Figure 4A) and it was observed that both liposomes and BV had the same transition temperature at 43°C. This was thought to indicate that proteins incorporated in the vesicles did embed deeply into the lipid bilayer. These results were in agreement with Molinaro et al. in which no significant change in the transition temperature is observed for BV generated with different protein to lipid ratios [10].

Liposomes and BV composed of DPPC:DSPE‐PEG_2000_ were observed by cryoTEM and the size distribution for both of them was found to be highly monodispersed (Figure 4B). In the case of liposomes, the morphology was predominantly spherical, unilamellar and univesicular, characterized by the negligible presence of multilamellar or multivesicular liposomes. GBM BV were found to have similar sizes and morphologies compared to liposomes, where spherical, unilamellar, and univesicular vesicles predominated. Higher magnification images allowed the visualization of the lipid bilayer in both liposomes and BV and confirmed their similar morphology. Some liposomes and BV were found to have a face‐edged, polygonal morphology, especially those with smaller sizes. This effect has been previously shown in the literature [58, 59, 60] and is due to the cryoTEM sample preparation, which was performed at 20°C, a temperature significantly lower than the gel‐to‐liquid crystalline phase transition temperature of both systems [61, 62]. At this temperature, both liposomes and BV's lipid bilayers show a more ordered and tightly‐packed structure, leading to the appearance of polygonal shapes. As a confirmation, liposomes were imaged after being warmed up at 50°C, displaying a more spherical morphology in most of the cases (Figure S8A). To build up on these findings, liposomes containing DMPC and DLPC as main phospholipids were also imaged (Figure S8B,C). In the case of DMPC, some polygonal morphologies were found, correlating with the fact that sample preparation was performed below the transition temperature of the lipid and therefore the lipid bilayer would be found in the gel‐phase. Nonetheless, the liposomes’ membrane was found to be less winding, which could be related with the fact that DMPC has a lower number of carbons in the hydrophobic tails compared to DPPC. For DLPC (T_t_ = −2°C), liposomes showed a generalized spherical morphology, due to the fact that the cryoTEM sample was performed above the transition temperature. Therefore, in this case the lipid bilayer was found in a fluid‐phase.

To test the robustness and reproducibility of the BV fabrication protocol developed, we repeated the preparation of BV using a colorectal cancer model immortalized cell line (MC38). As shown in Figure S9, MC38 protein extract‐containing vesicles were engineered and their hydrodynamic size, PDI, and surface charge were studied. The MC38‐derived BV showed similar morphologies compared to the GL261 equivalents, where unilamellar and univesicular vesicles predominated.

Neither the analysis performed by DSC nor cryoTEM could provide evidence supporting that the proteins from the extract were contained as part of the BV prepared in this study. As a result, performing proteomic analysis of these systems becomes crucial to demonstrate in an unequivocal manner the incorporation of proteins in BV, as well as providing deep knowledge of the identity of the proteins incorporated.

### 3.7 Proteomic Analysis of GBM‐Derived Vesicles

The thermal and morphological characterization shown above can provide only indirect evidence of protein incorporation onto BV. The amount of proteins incorporated onto the vesicles is a key feature for the future use of the BV, having a direct impact on the protein dose potentially administered. Demonstrating protein incorporation by direct protein quantification and identification in the BV is therefore fundamental to determine the potential capabilities and use of these systems, including their biological fate once inside the body.

**FIGURE 5:**
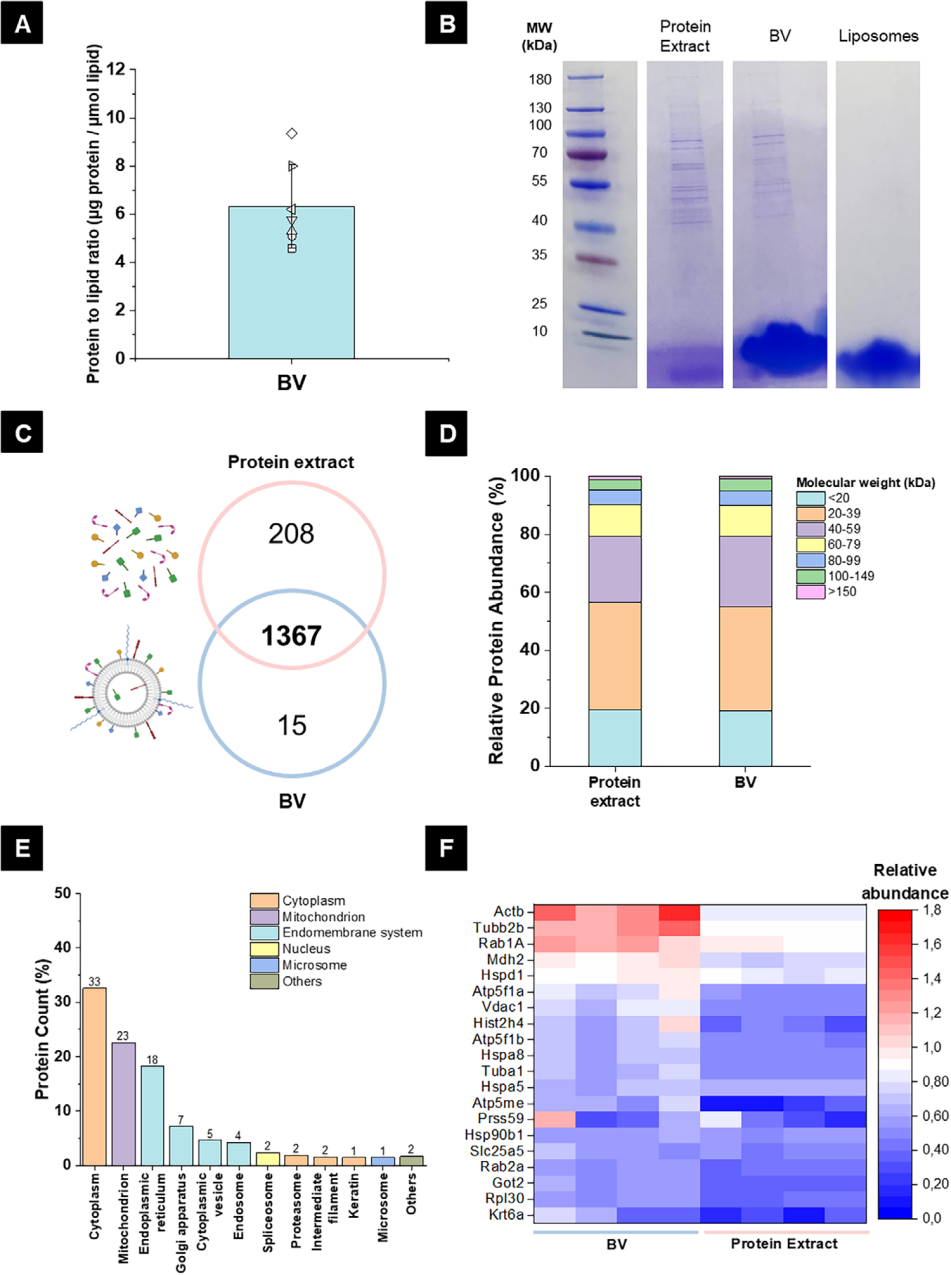
Proteomic analysis of BV prepared in this study. (A) Protein quantification in the BV represented by protein to lipid ratio, i.e. total protein content expressed in µg per µmol of lipid after purification and concentration. Data is represented by indicating the individual replicates in light blue dots (*n* = 7), the mean in a dark blue line and the standard deviation in black whiskers. (B) SDS‐PAGE gels of protein extract, DPPC:DSPE‐PEG_2000_ (1%) BV and liposomes. (C) Venn diagram indicating the unique proteins identified in the protein extract and BV, as well as the common identified proteins between both previously mentioned samples. The full list of the identified proteins is specified in Annex 1. (D) Relative protein abundances in protein extract and BV according to molecular weight. (E) Subcellular localization of the common proteins identified in the membrane protein extract and the BV, by correlating their genes into DAVID software and showing *p* < 0,01. Subcellular compartments with a protein count lower than 1% were included inside the category others (F) Relative abundance for the top 20 more abundant proteins identified (according to gene name) in the BV compared to the protein extract. The relation between the gene name, the protein name and the subcellular localization is specified in Table S1.

The BV composed of DPPC:DSPE‐PEG_2000_ prepared in this study were concentrated after purification and contained 6,3 ± 1,5 µg of protein per µmol of lipid (*n* = 7, prepared using the same stock of protein extract) (Figure 5A), measured by fluorescamine and Stewart assay (Figure S10). This value corresponded to around 10% of the initial protein amount and correlated with the values before concentration, suggesting that the process of concentration does not alter the protein: lipid content relation. These values are also in agreement with some of the studies previously reported [9, 38], nonetheless, it is worth pointing out that in general there is lack of consistency in the protein doses used for biological studies using biomimetic systems reported in the literature [16, 63, 64]. In addition, the dose of biomimetic systems is usually referred to as either the nanoparticle content or the lipid content [17, 65, 66], not the protein content.

Proteins incorporated in the BV were initially evaluated qualitatively by SDS‐PAGE. When loading the same volume of sample, it could be observed that both protein extract and BV shared similar protein bands (Figure 5B). This indicated that the biomimetic vesicle proteome contained similar features to the protein extract proteome. Liposomes of the same lipid composition were also run under the same conditions as a negative control for comparative purposes, showing the absence of any protein band but the characteristic stain for lipids, also present in BV.

Proteins present in the extract and in the BV were then analyzed quantitatively by LC‐MS/MS. As shown in Figure 5C, *n* = 1575 proteins were identified for the protein extract in comparison to the *n* = 1382 proteins identified in the BV. The protein list specifying the relative abundances, standard deviation, and cumulative values is shown in Table S1. Out of these proteins identified for each of the two groups, *n* = 1367 proteins were identified both in protein extract and BV, confirming the gel‐based evidence that the majority of proteins present in the extract were incorporated in the vesicles. In the BV there were n = 15 proteins that were not included in protein extract. This phenomenon is due to stochastic nature of LC‐MS/MS detection. Proteins that are present in very low abundance may be below the detection threshold for more complex samples like protein extract, but detectable in the BV in which they are borderline in abundance. The relative abundances of the proteins were also evaluated regarding their molecular weight and similar profiles were found between protein extract and BV (Figure 5D). It was observed that BV contained fewer large molecular weight proteins, suggesting a possible restriction in their incorporation, possibly leading to a spatial selection process.

The subcellular localization of the commonly identified proteins was studied using the bioinformatic tool DAVID [67, 68] after converting each of the common proteins identified into their corresponding encoded gene. As shown in Figure 5E, the subcellular localization of the genes is classified in different groups, schematized in different colors. A significant part of the proteins was related to the endosome system, belonging to organelles such as the endoplasmic reticulum, Golgi apparatus, sarcoplasmic reticulum, endosome and cytoplasmic vesicles. Some of the identified genes were associated to mitochondria and an important part was correlated to the cytoplasmic proteins, including cytoplasmic region as well as the proteasome, the intermediate filament and the keratin, forming part of the cytoskeleton. Finally, we also found some molecular contributions for spliceosomes, located inside the nucleus and microsomes. The result of this analysis suggested that membrane proteins from all cell compartments are contained in the BV, with a more heavily weighed contribution from cytosolic proteins [69], correlating as well with studies reported in the literature using the same protein extraction method [10, 70] or starting from cell‐lysates [22, 71, 72]. It is noteworthy to state that the protein extracts used in this study for the preparation of the BV were obtained using a commercial kit and protocol particularly developed (and marketed) for the extraction of membrane proteins. However, our results from LC/MS‐MS suggested that there is also a considerable contribution from cytosolic proteins contained in this protein extract. Generally, the total abundance of membrane proteins inside the cell is considerably low compared to the cytosolic fraction. Therefore, extracting a purified fraction of membrane proteins without cytosolic contamination should not be surprising.

The proteins common to both the extract and the BV were further evaluated by GO analysis. This analysis focused on proteins involved in adhesion, intercellular communication, intracellular trafficking, and other essential cell functions, aiming to highlight molecular mechanisms that may be relevant to cell behavior and interaction. With regards to proteins involved in cell adhesion, we identified the presence of different types of integrins, catenin, cadherin, and L1CAM. Cadherin, for instance is frequently overexpressed on cancer cell membranes and has been reported to promote self‐recognition through homotypic binding [73, 74, 75]. It was also identified the presence of some tetraspanins, like CD9 and CD63, which are involved in crucial cellular processes, such as cell migration, protein transport, maintenance of membrane integrity, and communication processes [76, 77]. Specifically, CD9 has been associated with oncogenic and pro‐metastatic functions [78]. Additionally, proteins involved in cell‐cell communication and immunity processes, like MHC‐I, CD44, and CD109, were also found. Some proteins related to cancer processes and tumor progression were also identified, such as the receptor CD276, and proteins related to HRAS and KRAS oncogenes. The receptor CD276, also known as B7‐H3, is highly expressed in cancerous and immune cells within the tumor microenvironment that acts as an immune checkpoint [79]. CD276 has been reported to promote invasion and angiogenesis and has been related to tumor cell proliferation, metastasis, and therapeutic resistance [80, 81]. Additionally, HRAS and KRAS have been associated with the initiation and progression of numerous cancer diseases [82, 83]. The presence of different chaperones was also noticeable, like calreticulin, HSP1, HSP5, HSP8, HSP40, and HSP90, as well as some proteins from the cytoskeleton, such as actin, keratin, tubulin, vimentin, among others. Finally, a substantial number of proteins related to metabolic and protein synthesis processes were identified, such as a various number of ATP synthase units, ion transporters, cytochromes, and different members of the RAS family, as well as ribosome units and proteins related to mRNA processing and splicing.

Clustering analysis using the STRING platform (k‐means algorithm) revealed that the majority of proteins enriched in the BV are involved in metabolic pathways and protein synthesis, consistent with the biological process enrichment results shown in Figure S11. The identified functional clusters included: (i) ribonucleoprotein complex; (ii) aerobic respiration and mitochondrion inner membrane components; (iii) Fatty acid beta‐oxidation; (iv) Golgi vesicle‐mediated transport; (v) endoplasmic reticulum organization; and (vi) sphingolipid metabolism and ceramide biosynthesis.

Common proteins between the extract and the BV were further analyzed using KEGG pathway mapping to identify those with potential roles in key cellular processes. For that purpose, Uniprot accession IDs from the 1367 common proteins between the extract and the BV were converted into KEGG‐compatible identifiers. Due to incomplete annotation of certain proteins, particularly predicted or unreviewed entries, not all identified proteins could be mapped, resulting in only 777 IDs included in the final KEGG analysis. Pathway mapping performed using KEGG Mapper revealed a predominant enrichment of proteins involved in core metabolic processes, particularly oxidative phosphorylation, glycolysis, and the TCA cycle, alongside strong representation of ribosomal and protein processing pathways. These findings indicate a coordinated regulation of energy metabolism and proteostasis, further supported by enrichment of pathways associated with oxidative stress, ER protein processing, and autophagy. Although several disease‐associated KEGG pathways were identified, these largely reflect shared molecular components related to mitochondrial function and protein homeostasis rather than disease‐specific mechanisms, with only a limited representation of pathways classically associated with cancer, including glioma. The complete list of pathways identified in this analysis can be found in Table S3. Collectively, these findings point to an integrated network of metabolic activity, protein turnover, and adaptive signaling processes, consistent with cellular states characterized by high biosynthetic demand and dynamic structural remodeling.

The common proteins between the extract and the BV were studied deeper by comparing the relative abundance of the 20 most abundant proteins in the BV to the extract alone. As seen in Figure 5F, the 20 most abundant proteins in the BV originated from different subcellular locations, such as the cytoskeleton in the case of actin, tubulin and keratin; the mitochondria, like malate dehydrogenase, ATP synthase and aspartate aminotransferase; and membrane proteins, such as the clusters of RAS‐related proteins, the voltage‐dependent anion‐selective channel protein 1 and the 78 kDa glucose‐regulated protein. Aside from the previously mentioned proteins, there were some chaperones like endoplasmin, the 60 kDa heat shock protein and the cluster of heat shock cognate, as well as histone H4 belonging to the nucleus and serine protease 59 located in the Golgi apparatus. When comparing the relative abundance of the 20 most abundant proteins between the BV with the protein extract alone, the BV did not exactly mirror that of the protein extract, offering another indication of possible molecular selectivity during the incorporation. Looking at the molecular weight of the top 20 most abundant proteins (Table S2), the majority component for the BV consisted of molecular weights lower than 60 kDa, meaning that smaller proteins incorporated better into the BV.

During the last decade a considerable number of studies have addressed the engineering of BV using various cell types, the most widely used have been red blood cells, mesenchymal stem cells, and different types of immune cells, such as leukocytes, monocytes and macrophages. More recently, the preparation of cancer‐cell derived vesicles has been reported by various groups, particularly incorporating components from breast cancer, melanoma, and osteosarcoma cells. Within the framework of GBM, only a few studies have been published to date [38, 39, 43]. In the works by Jia et al. and Li et al., cell membrane proteins were isolated and incorporated onto synthetic liposomes loaded with different drugs for enhanced anti‐tumor chemotherapy. Along a similar direction, the work developed by Zhou et al. described the engineering of a biomimetic hybrid vesicle, containing both macrophage and glioma cell membrane components and encapsulating a chemotherapeutic drug. These works are mainly focused on the evaluation of the therapeutic activity of the biomimetic systems and report an increased tumor cell uptake and anti‐tumor activity. With regards to proteomic analysis, our results align well with those reported in these works using SDS‐PAGE to qualitatively demonstrate that the proteome of the BV is similar to the cell source of the biological components used. Nonetheless, our work takes a step further in the proteomic analysis of the BV, providing a molecular insight of the cell membrane extract‐containing BV.

Our study has attempted to decipher some of the key parameters that can affect the engineering of GBM BV, including the effect of lipid composition and PEGylation, as well as some processing aspects related to their purification. To build on our findings, future work could explore the validation of the methodology proposed here, using protein extracts from other cell lines, as well as different biomimetic protein sources. In addition, the determination of the exact position and embedding of the biomimetic protein molecules incorporated onto the vesicles (lipid bilayer anchoring, surface adsorption, inner aqueous phase encapsulation) could also be investigated in the future.

The results obtained in this study can contribute to the optimization of the engineering of BV and provide meaningful insight on the proteomic character of the resulting BV. This directly links to the underlying biological mechanism and downstream therapeutic efficacy of the BV. As indicated by the proteomic analysis, the presence of different adhesion proteins, like cadherin, could offer substantial potential for homotypic targeting to GBM. Additionally, the presence of proteins overexpressed in different types of tumors, such as CD276, HRAS, and KRAS, could substantiate their use as immunotherapeutic agents. Moreover, the BV generated in this study could serve as promising cancer vaccination platforms, as they have demonstrated to be a reliable representation of the proteomic signature and therefore the antigenic spectra of the protein extract. In this direction, more future studies are warranted to evaluate the biological activity of the vesicles and their functional validation, as well as their capability to deliver therapeutic molecules or activate the immune system.

Although this work provides valuable and much‐needed insights for the rational design of BV, it is constrained by the use of protein extracts derived exclusively from immortalized cell cultures. This restricts how well the vesicles recapitulate biological conditions and does not fully capture the complexity of clinical samples. Future studies should explore alternative cell sources, including primary cells and tissue‐derived samples, to validate the robustness of the fabrication approach and to enhance its translational relevance.

## 4 Conclusions

In this study, glioblastoma BV have been prepared using protein extracts obtained from the GL261 cell line. Several key parameters for the preparation of GL261 BV were evaluated, leading to an optimized lipid composition based on DPPC:DSPE‐PEG_2000_ (1%). It was concluded that Sepharose CL‐4B solid phase was the most suitable matrix for purification of the biomimetic systems, according to the purification yield and separation ability between the BV and the non‐incorporated proteins. The morphological evaluation of the BV by cryoTEM confirmed their size homogeneity and unilamellarity with minimal deviations from the liposome alone. Finally, LC/MS‐MS results demonstrated that the proteomic component incorporated onto the BV was stoichiometrically equivalent to the protein contents from the cell extract, turning BV into a ´molecular reflection´ of the protein extracts. We also obtained some evidence of molecular selectivity in the incorporation of biomimetic vesicle proteins for those with smaller molecular weights. From the vesicle proteins identified, a considerable portion belonged to membrane‐associated domains, whereas a considerable fraction was associated with other cytoplasmic compartments. The engineering of these vesicles paves the way for the design of novel therapeutic vesicular platforms, whose envisioned applications could include cell‐specific immuno‐presentation in cancer vaccination.

## Author Contributions

N.H.L. contributed to the conceptualization of the study, carried out the experimental investigations, performed data curation and formal analysis, and wrote the original draft. K.K. conceptualized and supervised the study, including securing funding for the project. N.L. conceptualized and supervised the study and co‐curated data analysis. M.H. conceptualized and funded the MS experiments and contributed to the formal analysis of the data. H.A.M. performed the data curation and primary analysis of MS data. K.K., N.L., M.H., and H.A.M. all contributed to the revision and edit of the manuscript. M.C. contributed to the training and technical support in the cryo‐electron microscopy. P.G. contributed to the imaging acquisition in the cryo‐electron microscopy.

## Conflicts of Interest

The authors declare no conflicts of interest.

## Supporting information




**Supporting File 1**: adhm71222‐sup‐0001‐SuppMat.docx.

**Supporting File 2**: adhm71222‐sup‐0002‐TableS1.docx.

**Supporting File 3**: adhm71222‐sup‐0003‐TableS2.docx.

**Supporting File 4**: adhm71222‐sup‐0004‐TableS3.docx.

## Data Availability

The data that support the findings of this study are available from the corresponding author upon reasonable request.

## References

[1] J. W. Yoo , D. J. Irvine , D. E. Discher , and S. Mitragotri , “Bio‐inspired, Bioengineered and Biomimetic Drug Delivery Carriers,” Nature Reviews Drug Discovery 10 (2011): 521–535, 10.1038/nrd3499. 21720407

[2] S. Y. Fam , C. F. Chee , C. Y. Yong , K. L. Ho , A. R. Mariatulqabtiah , and W. S. Tan , “Stealth Coating of Nanoparticles in Drug‐Delivery Systems,” Nanomaterials 10 (2020): 787, 10.3390/nano10040787. 32325941 PMC7221919

[3] H. Wang , Y. Liu , R. He , et al., “Cell Membrane Biomimetic Nanoparticles for Inflammation and Cancer Targeting in Drug Delivery,” Biomaterials Science 8 (2020): 552–568, 10.1039/C9BM01392J. 31769765

[4] P. Dash , A. M. Piras , and M. Dash , “Cell Membrane Coated Nanocarriers—An Efficient Biomimetic Platform for Targeted Therapy,” Journal of Controlled Release 327 (2020): 546–570, 10.1016/j.jconrel.2020.09.012. 32911013

[5] J. Liu , S. S. Liew , J. Wang , and K. Pu , “NIR‐Activated Phototheranostic Nanomaterials for Cancer Therapy,” Advanced Materials 34 (2022): 1.

[6] R. H. Fang , A. V. Kroll , W. Gao , and L. Zhang , “Cell Membrane‐Coated Nanotechnology,” Advanced Materials 30 (2018): 1. 10.1002/adma.201706759 PMC5984176 29582476

[7] M. F. Mougenot , V. S. Pereira , A. L. R. Costa , et al., “Biomimetic Nanovesicles—Sources, Design, Production Methods, and Applications,” Pharmaceutics 14 (2022): 2008, 10.3390/pharmaceutics14102008. 36297442 PMC9610935

[8] N. E. Toledano Furman , Y. Lupu‐Haber , T. Bronshtein , et al., “Reconstructed Stem Cell Nanoghosts: a Natural Tumor Targeting Platform,” Nano Letters 13 (2013): 3248–3255, 10.1021/nl401376w. 23786263

[9] H. Cao , Z. Dan , X. He , et al., “Liposomes Coated with Isolated Macrophage Membrane Can Target Lung Metastasis of Breast Cancer,” ACS Nano 10 (2016): 7738–7748, 10.1021/acsnano.6b03148. 27454827

[10] R. Molinaro , C. Corbo , J. O. Martinez , et al., “Biomimetic Proteolipid Vesicles for Targeting Inflamed Tissues,” Nature Materials 15 (2016): 1037–1046, 10.1038/nmat4644. 27213956 PMC5127392

[11] A. Zinger , G. Baudo , T. Naoi , et al., “Reproducible and Characterized Method for Ponatinib Encapsulation into Biomimetic Lipid Nanoparticles as a Platform for Multi‐Tyrosine Kinase‐Targeted Therapy,” ACS Applied Bio Materials 3 (2020): 6737–6745, 10.1021/acsabm.0c00685. 35019338

[12] C. Corbo , W. E. Cromer , R. Molinaro , et al., “Engineered Biomimetic Nanovesicles Show Intrinsic Anti‐inflammatory Properties for the Treatment of Inflammatory Bowel Diseases,” Nanoscale 9 (2017): 14581–14591, 10.1039/C7NR04734G. 28932838

[13] D. De Pasquale , C. Pucci , A. Desii , et al., “MXene‐Based Biointerfaces for Advanced Health Care Applications,” Advanced Healthcare Materials 12 (2023): 1.

[14] M. A. Scully , D. E. Wilkins , M. N. Dang , E. C. Hoover , S. B. Aboeleneen , and E. S. Day , “Cancer Cell Membrane Wrapped Nanoparticles for the Delivery of a Bcl‐2 Inhibitor to Triple‐Negative Breast Cancer,” Molecular Pharmaceutics 20 (2023): 3895–3913, 10.1021/acs.molpharmaceut.3c00009. 37459272 PMC10628893

[15] W. J. Goh , S. Zou , B. Czarny , and G. Pastorin , “nCVTs: A Hybrid Smart Tumour Targeting Platform,” Nanoscale 10 (2018): 6812–6819, 10.1039/C7NR08720A. 29595203

[16] H. He , C. Guo , J. Wang , et al., “Leutusome: a Biomimetic Nanoplatform Integrating Plasma Membrane Components of Leukocytes and Tumor Cells for Remarkably Enhanced Solid Tumor Homing,” Nano Letters 18 (2018): 6164–6174, 10.1021/acs.nanolett.8b01892. 30207473 PMC6292712

[17] J. Oieni , A. Lolli , D. D'Atri , et al., “Nano‐ghosts: Novel Biomimetic Nano‐vesicles for the Delivery of Antisense Oligonucleotides,” Journal of Controlled Release 333 (2021): 28–40, 10.1016/j.jconrel.2021.03.018. 33741386

[18] Y. Su , T. Huang , H. Sun , et al., “MXene‐Based Nanoplatforms for Advanced Biomedical Applications,” Advanced Healthcare Materials 12 (2023): 1.

[19] A. Zinger , C. Cvetkovic , M. Sushnitha , et al., “Nanoparticle Tracking and Distribution in Biological Systems Using Advanced Imaging Platforms,” Advancement of Science 8 (2021): 1.

[20] N. Zhang , J. Lin , and S. Y. Chew , “Neural Cell Membrane‐Coated Nanoparticles for Targeted and Enhanced Uptake by Central Nervous System Cells,” ACS Applied Materials & Interfaces 13 (2021): 55840–55850, 10.1021/acsami.1c16543. 34792341

[21] R. H. Fang , C. M. J. Hu , B. T. Luk , et al., “Cancer Cell Membrane‐Coated Nanoparticles for Anticancer Vaccination and Drug Delivery,” Nano Letters 14 (2014): 2181–2188, 10.1021/nl500618u. 24673373 PMC3985711

[22] G. Deng , Z. Sun , S. Li , et al., “Cell‐Membrane Immunotherapy Based on Natural Killer Cell Membrane Coated Nanoparticles for the Effective Inhibition of Primary and Abscopal Tumor Growth,” ACS Nano 12 (2018): 12096–12108, 10.1021/acsnano.8b05292. 30444351

[23] H. Y. Kim , M. Kang , Y. W. Choo , et al., “Immunomodulatory Lipocomplex Functionalized with Photosensitizer‐Embedded Cancer Cell Membrane Inhibits Tumor Growth and Metastasis,” Nano Letters 19 (2019): 5185–5193, 10.1021/acs.nanolett.9b01571. 31298024

[24] C. Liu , X. Liu , X. Xiang , et al., “MXene‐Based Flexible Electronics and Wearable Systems: Progress and Prospects,” Nature Nanotechnology 17 (2022): 947–963, 10.1038/s41565-022-01098-0.

[25] R. Cheng , F. Fontana , J. Xiao , et al., “Recombination Monophosphoryl Lipid A‐Derived Vacosome for the Development of Preventive Cancer Vaccines,” ACS Applied Materials & Interfaces 12 (2020): 44554–44562, 10.1021/acsami.0c15057. 32960566 PMC7549091

[26] R. Molinaro , M. Evangelopoulos , J. R. Hoffman , et al., “Design and Development of Biomimetic Nanovesicles Using a Microfluidic Approach,” Advanced Materials 30 (2018): 1, 10.1002/adma.201702749. 29512198

[27] A. Zinger , M. Sushnitha , T. Naoi , et al., “Enhancing Inflammation Targeting Using Tunable Leukocyte‐Based Biomimetic Nanoparticles,” ACS Nano 15 (2021): 6326–6339, 10.1021/acsnano.0c05792. 33724785 PMC8155322

[28] I. Arduino , R. Di Fonte , M. Tiboni , et al., “Microfluidic Development and Biological Evaluation of Targeted Therapy‐loaded Biomimetic Nano System to Improve the Metastatic Melanoma Treatment,” International Journal of Pharmaceutics 650 (2024): 123697, 10.1016/j.ijpharm.2023.123697. 38081557

[29] J. Oieni , L. Levy , N. Letko Khait , et al., “Nano‐Ghosts: Biomimetic Membranal Vesicles, Technology and Characterization,” Methods 177 (2020): 126–134, 10.1016/j.ymeth.2019.11.013. 31794834

[30] R. Rampado , A. Biccari , E. D'Angelo , et al., “Optimization of Biomimetic, Leukocyte‐Mimicking Nanovesicles for Drug Delivery against Colorectal Cancer Using a Design of Experiment Approach,” Frontiers in Bioengineering and Biotechnology 10 (2022): 1, 10.3389/fbioe.2022.883034. PMC9214241 35757799

[31] Q. Jiang , K. Wang , X. Zhang , et al., “MXene‐Based Functional Materials for Energy Storage and Conversion Applications,” Small 16 (2020): 1.

[32] J. Lyu , R. Shao , P. Y. Kwong Yung , and S. J. Elsässer , “Genome‐wide Mapping of G‐quadruplex Structures with CUT&Tag,” Nucleic Acids Research 50 (2022): 13, 10.1038/s41467-022-31799-y. PMC8860588 34792172

[33] V. Nica , A. Marino , C. Pucci , et al., “Cell‐Membrane‐Coated and Cell‐Penetrating Peptide‐Conjugated Trimagnetic Nanoparticles for Targeted Magnetic Hyperthermia of Prostate Cancer Cells,” ACS Applied Materials & Interfaces 15 (2023): 30008–30028, 10.1021/acsami.3c07248. 37312240 PMC10316402

[34] A. Thakur , C. Faujdar , R. Sharma , et al., “Glioblastoma: Current Status, Emerging Targets, and Recent Advances,” Journal of Medicinal Chemistry 65 (2022): 8596–8685, 10.1021/acs.jmedchem.1c01946. 35786935 PMC9297300

[35] J. Hawly , M. G. Murcar , A. Schcolnik‐Cabrera , and M. E. Issa , “Glioblastoma Stem Cell Metabolism and Immunity,” Cancer and Metastasis Reviews 43 (2024): 1015–1035, 10.1007/s10555-024-10183-w. 38530545

[36] A. Omuro and L. M. DeAngelis , “Glioblastoma and Other Malignant Gliomas,” Jama 310 (2013): 1842–1850, 10.1001/jama.2013.280319. 24193082

[37] X. Y. Lim , S. M. Capinpin , N. Bolem , et al., “Biomimetic Nanotherapeutics for Targeted Drug Delivery to Glioblastoma Multiforme,” Bioengineering & Translational Medicine 8 (2023): 1, 10.1002/btm2.10483. PMC10189489 37206213

[38] Y. Jia , Z. Sheng , D. Hu , et al., “Highly Penetrative Liposome Nanomedicine Generated by a Biomimetic Strategy for Enhanced Cancer Chemotherapy,” Biomaterials Science 6 (2018): 1546–1555, 10.1039/C8BM00256H. 29694474

[39] J. Li , H. Zeng , Y. You , et al., “MXene‐Based Nanomaterials for Biomedical Applications: A Review,” Journal of Nanobiotechnology 19 (2021): 1.33397416

[40] Q. Xu , X. Hu , I. Ullah , et al., “Biomimetic Hybrid PROTAC Nanovesicles Block Multiple DNA Repair Pathways To Overcome Temozolomide Resistance Against Orthotopic Glioblastoma,” Advanced Materials 37 (2025): 2504253, 10.1002/ADMA.202504253. 40347032

[41] I. Arduino , R. Di Fonte , F. Sommonte , et al., “Fabrication of Biomimetic Hybrid Liposomes via Microfluidic Technology: Homotypic Targeting and Antitumor Efficacy Studies in Glioma Cells,” International Journal of Nanomedicine 19 (2024): 13217–13233, 10.2147/IJN.S489872. 39679250 PMC11638480

[42] P. Liu , S. Lan , D. Gao , et al., “Targeted Blood‐brain Barrier Penetration and Precise Imaging of Infiltrative Glioblastoma Margins Using Hybrid Cell Membrane‐coated ICG Liposomes,” Journal of Nanobiotechnology 22 (2024): 603, 10.1186/s12951-024-02870-1. 39367395 PMC11452969

[43] M. Zhou , Y. Wu , M. Sun , et al., “Spatiotemporally Sequential Delivery of Biomimetic Liposomes Potentiates Glioma Chemotherapy,” Journal of Controlled Release 365 (2024): 876–888, 10.1016/j.jconrel.2023.11.046. 38030082

[44] L. Papafilippou , A. Nicolaou , A. C. Kendall , D. Camacho‐Muñoz , and M. Hadjidemetriou , “The Lipidomic Profile of the Nanoparticle‐biomolecule Corona Reflects the Diversity of Plasma Lipids,” Nanoscale 15 (2023): 11038–11051, 10.1039/D2NR05982G. 37357917

[45] J. P. M. Andrews , S. S. Joshi , E. Tzolos , et al., “First‐in‐Human Controlled Inhalation of Thin Graphene Oxide Nanosheets To Study Acute Cardiorespiratory Responses,” Nature Nanotechnology 19 (2024): 705–714, 10.1038/s41565-023-01572-3. PMC11106005 38366225

[46] M. Hadjidemetriou , S. McAdam , G. Garner , et al., “The Evolving Landscape of Nanoparticle Tracking in Biological Systems: From Imaging to Proteomics,” Advanced Materials 31 (2019): 1.

[47] S. Bünger , U. J. Roblick , and J. K. Habermann , “Tumor‐Derived Cell Lines as Models for Colorectal Cancer Research: Molecular Characterization and Applications,” Cytotechnology 61 (2009): 153–165.20072854

[48] H. Yi , J. D. Strauss , Z. Ke , et al., “Native Immunogold Labeling of Cell Surface Proteins and Viral Glycoproteins for Cryo‐Electron Microscopy and Cryo‐Electron Tomography Applications,” Journal of Histochemistry & Cytochemistry 63 (2015): 780–792, 10.1369/0022155415593323. 26069287 PMC4823802

[49] P. J. Peters , E. Bos , and A. Griekspoor , “Fluorescence Microscopy of Cells in Culture,” Current Protocols in Cell Biology 00 (2006): 4.3.1–4.3.6, 10.1002/0471143030.CB0407S30. 18228493

[50] M. L. Briuglia , C. Rotella , A. McFarlane , and D. A. Lamprou , “Influence of Cholesterol on Liposome Stability and on in Vitro Drug Release,” Drug Delivery and Translational Research 5 (2015): 231–242, 10.1007/s13346-015-0220-8. 25787731

[51] U. Bulbake , S. Doppalapudi , N. Kommineni , and W. Khan , “Liposomal Formulations in Clinical Use: an Updated Review,” Pharmaceutics 9 (2017): 12, 10.3390/pharmaceutics9020012. 28346375 PMC5489929

[52] F. M. Veronese and G. Pasut , “PEGylation, Successful Approach to Drug Delivery,” Drug Discovery Today 10 (2005): 1451–1458, 10.1016/S1359-6446(05)03575-0. 16243265

[53] S. Zalba , T. L. M. ten Hagen , C. Burgui , and M. J. Garrido , “Stealth Nanoparticles in Oncology: Facing the PEG Dilemma,” Journal of Controlled Release 351 (2022): 22–36, 10.1016/j.jconrel.2022.09.002. 36087801

[54] B. M. Chen , T. L. Cheng , and S. R. Roffler , “Polyethylene Glycol Immunogenicity: Theoretical, Clinical, and Practical Aspects of Anti‐Polyethylene Glycol Antibodies,” ACS Nano 15 (2021): 14022–14048, 10.1021/acsnano.1c05922. 34469112

[55] M. Hadjidemetriou , Z. Al‐Ahmady , M. Mazza , R. F. Collins , K. Dawson , and K. Kostarelos , “In Vivo Biomolecule Corona around Blood‐Circulating, Clinically Used and Antibody‐Targeted Lipid Bilayer Nanoscale Vesicles,” ACS Nano 9 (2015): 8142–8156, 10.1021/acsnano.5b03300. 26135229

[56] M. Hadjidemetriou , L. Papafilippou , R. D. Unwin , J. Rogan , A. Clamp , and K. Kostarelos , “Nano‐Scavengers for Blood Biomarker Discovery in Ovarian Carcinoma,” Nano Today 34 (2020): 100901, 10.1016/j.nantod.2020.100901.

[57] C. Demetzos , “Differential Scanning Calorimetry (DSC): a Tool to Study the Thermal Behavior of Lipid Bilayers and Liposomal Stability,” Journal of Liposome Research 18 (2008): 159–173, 10.1080/08982100802310261. 18770070

[58] L. M. Ickenstein , M. C. Arfvidsson , D. Needham , L. D. Mayer , and K. Edwards , “Disc Formation in Cholesterol‐free Liposomes during Phase Transition,” Biochimica et Biophysica Acta (BBA)—Biomembranes 1614 (2003): 135–138, 10.1016/S0005-2736(03)00196-2. 12896806

[59] L. M. Ickenstein , M. C. Sandström , L. D. Mayer , and K. Edwards , “Effects of Phospholipid Hydrolysis on the Aggregate Structure in DPPC/DSPE‐PEG2000 Liposome Preparations after Gel to Liquid Crystalline Phase Transition,” Biochimica et Biophysica Acta (BBA)—Biomembranes 1758 (2006): 171–180, 10.1016/j.bbamem.2006.02.016. 16574061

[60] L. Farkuh , P. T. Hennies , C. Nunes , et al., “Characterization of Phospholipid Vesicles Containing Lauric Acid: Physicochemical Basis for Process and Product Development,” Heliyon 5 (2019): e02648, 10.1016/j.heliyon.2019.e02648. 31720452 PMC6838897

[61] M. Andersson , L. Hammarström , and K. Edwards , “Effect of Bilayer Phase Transitions on Vesicle Structure, and Its Influence on the Kinetics of Viologen Reduction,” The Journal of Physical Chemistry 99 (1995): 14531–14538, 10.1021/j100039a047.

[62] M. M. Zetterberg , S. Ahlgren , V. Agmo Hernández , N. Parveen , and K. Edwards , “Optimization of Lipodisk Properties by Modification of the Extent and Density of the PEG Corona,” Journal of Colloid and Interface Science 484 (2016): 86–96, 10.1016/j.jcis.2016.08.067. 27592189

[63] C.‐M. J. Hu , R. H. Fang , K.‐C. Wang , et al., “Nanoparticle Biointerfacing by Platelet Membrane Cloaking,” Nature 526 (2015): 118–121, 10.1038/nature15373. 26374997 PMC4871317

[64] C. D. Pack , R. Bommireddy , L. E. Munoz , et al., “Tumor Membrane‐based Vaccine Immunotherapy in Combination with Anti‐CTLA‐4 Antibody Confers Protection against Immune Checkpoint Resistant Murine Triple‐negative Breast Cancer,” Human Vaccines & Immunotherapeutics 16 (2020): 3184–3193, 10.1080/21645515.2020.1754691. 32530786 PMC8641616

[65] Y. Lupu‐Haber , T. Bronshtein , H. Shalom‐Luxenburg , et al., “Pretreating Mesenchymal Stem Cells with Cancer Conditioned‐Media or Proinflammatory Cytokines Changes the Tumor and Immune Targeting by Nanoghosts Derived from these Cells,” Advanced Healthcare Materials 8 (2019): 1, 10.1002/adhm.201801589. 30963725

[66] R. Molinaro , J. O. Martinez , A. Zinger , et al., “Leukocyte‐Mimicking Nanovesicles for Effective Doxorubicin Delivery to Treat Breast Cancer and Melanoma,” Biomaterials Science 8 (2020): 333–341, 10.1039/C9BM01766F. 31714542

[67] D. W. Huang , B. T. Sherman , and R. A. Lempicki , “Systematic and Integrative Analysis of Large Gene Lists Using DAVID Bioinformatics Resources,” Nature Protocols 4 (2009): 44–57, 10.1038/nprot.2008.211. 19131956

[68] B. T. Sherman , M. Hao , J. Qiu , et al., “DAVID: A Web Server for Functional Enrichment Analysis and Functional Annotation of Gene Lists (2021 update),” Nucleic Acids Research 50 (2022): W216–W221, 10.1093/nar/gkac194. 35325185 PMC9252805

[69] A. O. Helbig , A. J. R. Heck , and M. Slijper , “Exploring the Membrane Proteome—Challenges and Analytical Strategies,” Journal of Proteomics 73 (2010): 868–878, 10.1016/j.jprot.2010.01.005. 20096812

[70] C. Corbo , A. Parodi , M. Evangelopoulos , et al., “Proteomic Profiling of a Biomimetic Drug Delivery Platform,” Current Drug Targets 16 (2014): 1540–1547, 10.2174/1389450115666141109211413. PMC4426090 25382209

[71] A. Nasiri Kenari , K. Kastaniegaard , D. W. Greening , “Proteomic Insights into Extracellular Vesicles: Methods and Biological Applications,” et al., Proteomics 19 (2019): 1.

[72] Y. R. Neupane , H. K. Handral , S. A. Alkaff , et al., “Nanomedicine Strategies for Targeted Drug Delivery and Cancer Therapy: Recent Advances and Challenges,” Acta Pharmaceutica Sinica B 13 (2023): 1008–1035, 10.1016/j.apsb.2022.10.022.

[73] A. S. Sultan , J. Xie , M. J. LeBaron , E. L. Ealley , M. T. Nevalainen , and H. Rui , “Stat5 Promotes Homotypic Adhesion and Inhibits Invasive Characteristics of Human Breast Cancer Cells,” Oncogene 24 (2005): 746–760, 10.1038/sj.onc.1208203. 15592524

[74] L. Chen , W. Hong , W. Ren , T. Xu , Z. Qian , and Z. He , “Recent Progress in Targeted Delivery Vectors Based on Biomimetic Nanoparticles,” Signal Transduction and Targeted Therapy 6 (2021): 225, 10.1038/s41392-021-00631-2. 34099630 PMC8182741

[75] F. N. Arslan , J. Eckert , T. Schmidt , and C. P. Heisenberg , “Holding It Together: When cadherin Meets Cadherin,” Biophysical Journal 120 (2021): 4182–4192, 10.1016/j.bpj.2021.03.025. 33794149 PMC8516678

[76] K. J. Susa , A. C. Kruse , and S. C. Blacklow , “Tetraspanins: Structure, Dynamics, and Principles of Partner‐protein Recognition,” Trends in Cell Biology 34 (2024): 509–522, 10.1016/j.tcb.2023.09.003. 37783654 PMC10980598

[77] M. E. Hemler , “Tetraspanin Functions and Associated Microdomains,” Nature Reviews Molecular Cell Biology 6 (2005): 801–811, 10.1038/nrm1736. 16314869

[78] M. E. Hemler , “Tetraspanin Proteins Promote Multiple Cancer Stages,” Nature Reviews Cancer 14 (2013): 49–60, 10.1038/nrc3640. 24505619

[79] M. Ding , H. Liao , N. Zhou , Y. Yang , S. Guan , and L. Chen , “B7‐H3‐Induced Signaling in Lung Adenocarcinoma Cell Lines with Divergent Epidermal Growth Factor Receptor Mutation Patterns,” BioMed Research International 2020 (2020): 8824805, 10.1155/2020/8824805. 33426073 PMC7775133

[80] A. A. Getu , A. Tigabu , M. Zhou , J. Lu , Ø. Fodstad , and M. Tan , “New Frontiers in Immune Checkpoint B7‐H3 (CD276) Research and Drug Development,” Molecular Cancer 22 (2023): 43, 10.1186/s12943-023-01751-9. 36859240 PMC9979440

[81] N. Cheng , Y. Bei , Y. Song , et al., “B7‐H3 augments the Pro‐angiogenic Function of Tumor‐associated Macrophages and Acts as a Novel Adjuvant Target for Triple‐negative Breast Cancer Therapy,” Biochemical Pharmacology 183 (2021): 114298, 10.1016/j.bcp.2020.114298. 33153969

[82] D. Johnson , C. E. Chee , W. Wong , R. C. T. Lam , I. B. H. Tan , and B. B. Y. Ma , “Advances in Nanomedicine for Cancer Therapy: Emerging Platforms and Clinical Translation Challenges,” Cancer Treatment Reviews 125 (2024): 102715, 10.1016/J.CTRV.2024.102700. 38422896

[83] M. Pązik , K. Michalska , M. Żebrowska‐Nawrocka , I. Zawadzka , M. Łochowski , and E. Balcerczak , “Clinical Significance of HRAS and KRAS Genes Expression in Patients with Non–small‐cell Lung Cancer—Preliminary Findings,” BMC Cancer 21 (2021): 130, 10.1186/s12885-021-07858-w. 33549031 PMC7866659

