# Supplementary material for: Engineering of Glioblastoma‐Derived Biomimetic Vesicles and Their Structural and Molecular Features
Source: Adv Healthc Mater. 2026 May 8;15(23):e03775. doi: 10.1002/adhm.202503775 (PMC13280188; doi:10.1002/adhm.202503775)
Supplement: Supplementary file 1 — Supporting File 1: adhm71222‐sup‐0001‐SuppMat.docx. [file ADHM-15-0-s001.docx]

## Supporting Experimental

***BCA assay.*** The measurements of protein concentration by BCA assay were performed as indicated by manufacturer of Pierce™ BCA Protein assay Kit (ThermoScientific). Briefly, 25 µL of sample were mixed with 200 µL of working reagent (prepared by mixing 50 parts of reagent A and 1 part of reagent B). Samples were mildly shaked for 10 seconds and incubated at 37°C for 30 min. Then, the absorbance at 562 nm was measured in the plate reader SpectraMax® iD3 at ICN2 Nanobioelectronics and Biosensors Group. Each sample was prepared in triplicates. The absorbance of each sample was interpolated into a calibration curve prepared with BSA, ranging from 10 to 750 µg/mL.

***Cryo-electron transmission microscopy.*** The native morphology of the BV was studied by cryo-electron transmission microscopy (CryoTEM) at the Microscopy and X-ray diffraction service at UAB. Briefly, the sample was vitrified by depositing 3.9 µL of vesicles in a previously glow-discharged grid (Micro to Nano, EMR Lacey Carbon support film on copper 400 square mesh), blotted for 2.5 s and plunged into liquid ethane at -186°C (Leica EM GP cryo-plunger). The grid was then transferred into a cryoholder (GATAN) and visualised in a JEOL-2011 transmission electron microscope adapted for cryogenic microscopy, working at a voltage of 200kV and equipped with CMOS Gatan Rio 16 camera and EDS Oxford Instruments X-max detector. The images were taken using an exposition time of 1 to 2 s.

## Supporting Figures


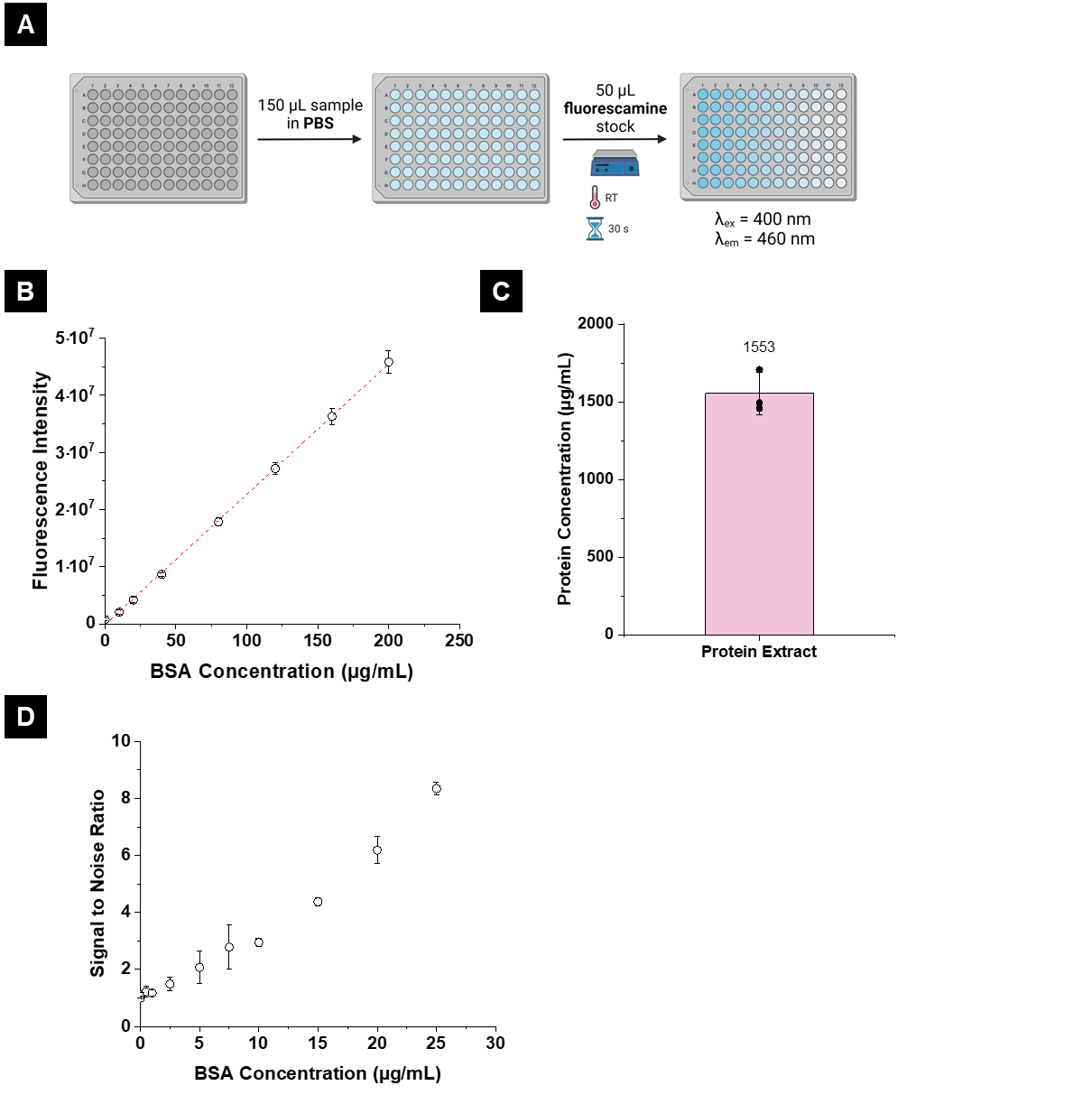


**Figure S1.** **Protein quantification in protein extract obtained from GL261 cell line by fluorescamine assay. A)** General protocol for the measurement of protein content by fluorescamine assay. Briefly, the sample in PBS is added in a 96 well plate together with a stock fluorescamine solution at 3 mg/mL in DMSO. After mild shaking, the fluorescence intensity at 460 nm is measured (λ_em_ = 400 nm). **B)** Calibration curve for fluorescamine assay by representing fluorescence intensity measured at 460 nm versus the concentration of bovine serum albumin (BSA) between 10 and 200 µg/mL. Data represented as the mean ± standard deviation of at least n=3. **C)** Quantification of protein content in protein extract obtained from GL261 cell line using fluorescamine assay. Data is represented as the mean ± SD (n=3) indicating the individual replicates. **D)** Signal to noise ratio in fluorescamine assay with respect to BSA concentration. Data represented as the mean ± standard deviation of at least n=3.


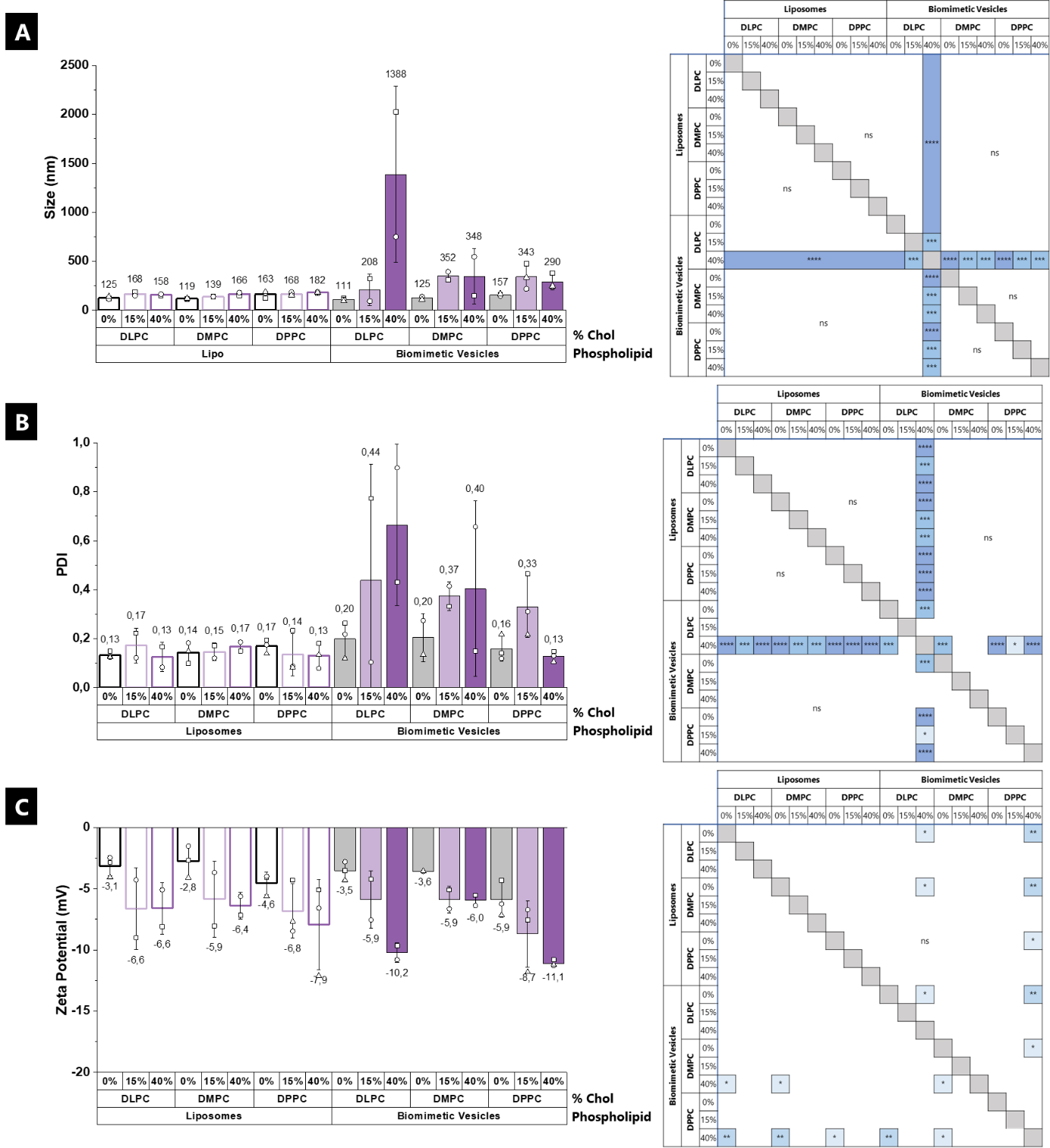


**Figure S2.** **Engineering of the lipid formulation of BV according to the phospholipid length and cholesterol content.** Evaluation of **A)** size and **B)** polydispersity index by dynamic light scattering and **C)** zeta potential by electrophoretic mobility of liposomes (empty bars, L) and BV (filled bars, V) composed of DPPC, DMPC and DLPC and 0% (black), 15% (light purple) and 40% (dark purple) cholesterol content. Data belonging to DPPC corresponds to the one shown in Figure 2. Data is represented as the mean ± standard deviation of at least n=2. Statistical significance is displayed in compact significance matrix analyses with coloured cells indicating statistically significant pairwise differences (p < 0.05) between formulations. Non-coloured cells denote comparisons without statistical significance (ns).


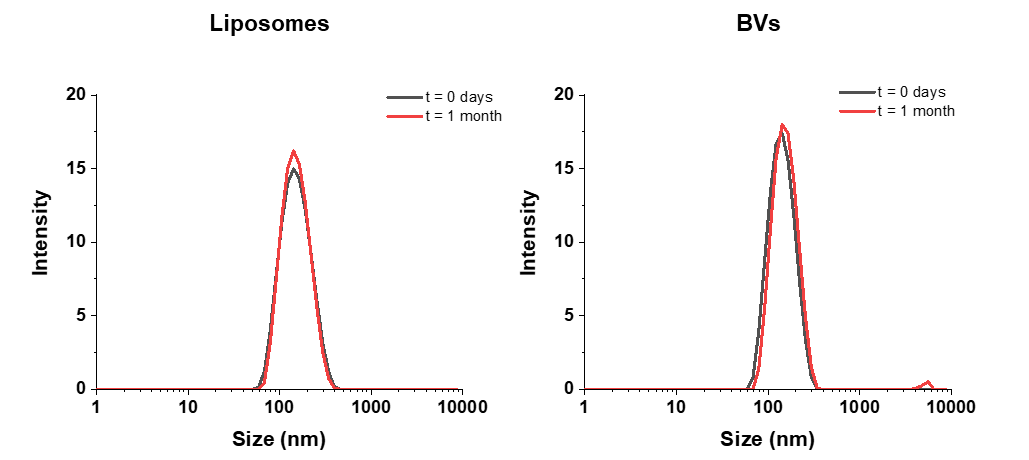


**Figure S3. Colloidal stability over time of liposomes and BV made of DPPC:DSPE-PEG (1%).** Size distribution curves of liposomes and BV obtained by Dynamic Light Scattering at times 0 and 1 month.


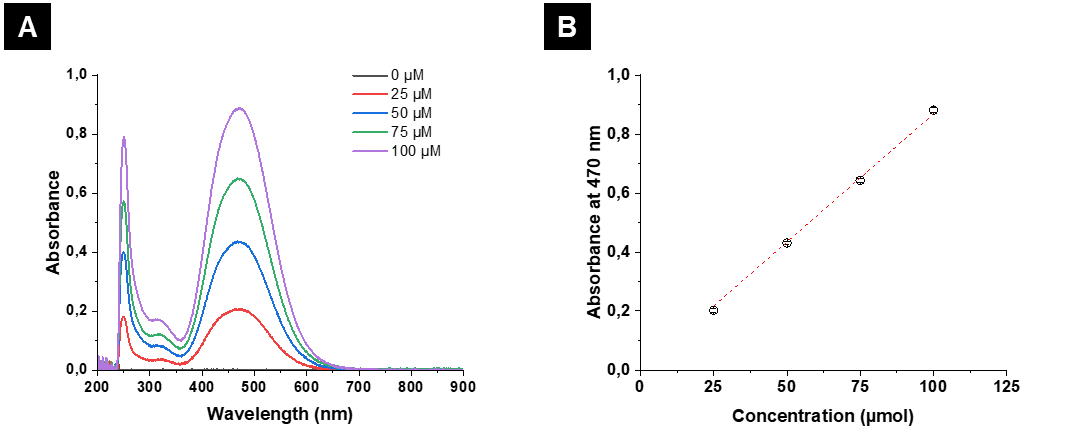


**Figure S4.** **Calibration curve used for lipid quantification by Stewart assay. A)** UV-Vis absorbance spectra of Stewart assay calibration samples composed of DPPC:DSPE-PEG_2000_ (1%). **B)** Calibration curve of the absorbance at 470 nm used for lipid quantification by Stewart assay for DPPC:DSPE-PEG_2000_ (1%) liposomes and BV samples. Data represented as the mean ± standard deviation of n=2.

**
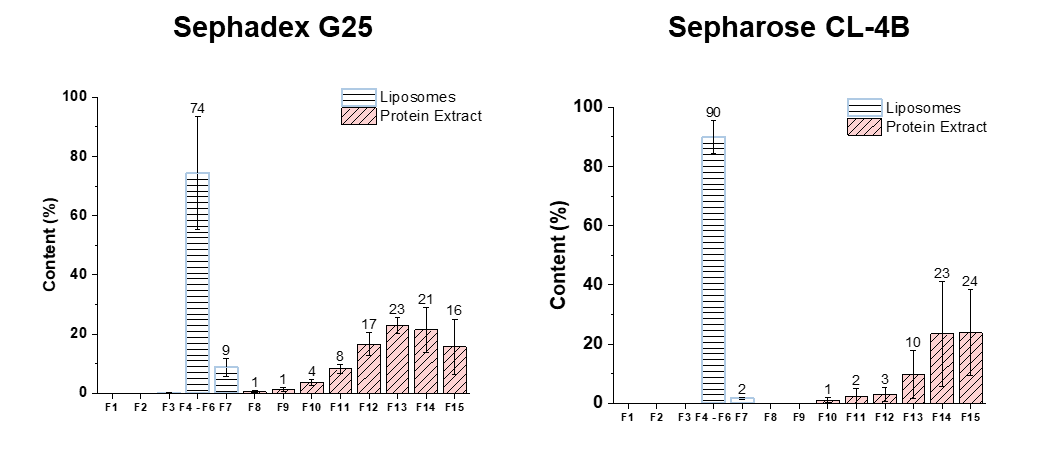
**

**Figure S5.** **Purification of BV composed of DPPC:DSPE-PEG_2000_ (1%) by size exclusion chromatography using Sephadex G25 and Sepharose CL-4B as solid phases.** Quantification in percentage of the lipid and protein content in the SEC fractions, after the separate purification of liposomes (empty blue bars) and GL261 protein extract (pink bars) by Stewart assay (horizontal lines) and fluorescamine assay (diagonal lines), respectively. Signals measured by fluorescamine assay corresponding to protein concentrations below 10 µg/mL were not considered as they were under the limit of quantification. Data is represented as mean ± standard deviation of at least n=3 individual replicates.


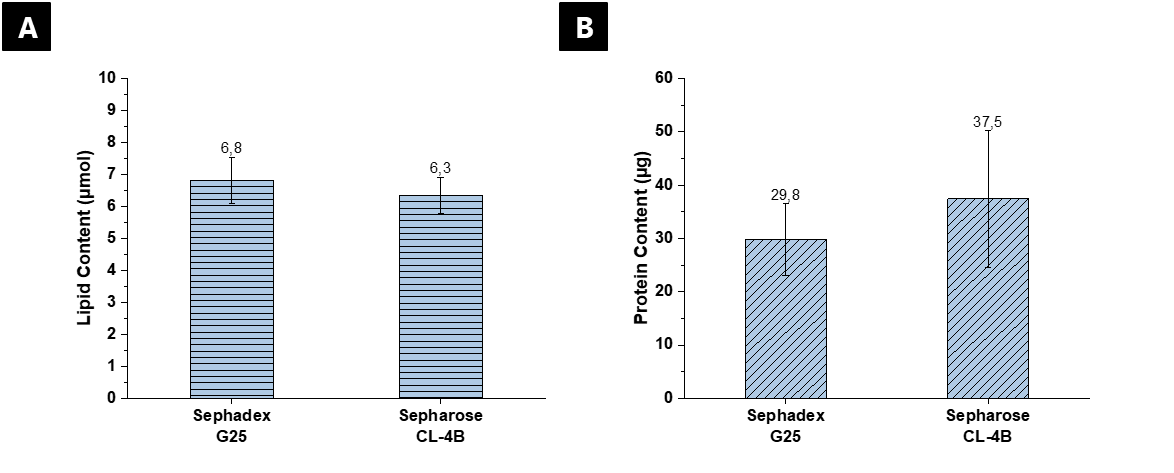


**Figure S6.** **Determination of lipid and protein content in BV** composed of DPPC:DSPE-PEG_2000_ (1%) after purification by size exclusion chromatography using Sephadex G25 and Sepharose CL-4B as solid phases. **A)** Lipid content (expressed in µmol) and **B)** protein content, determined by Stewart and fluorescamine assay, respectively. Data is represented as the mean ± standard deviation of at least n=3 individual replicates.


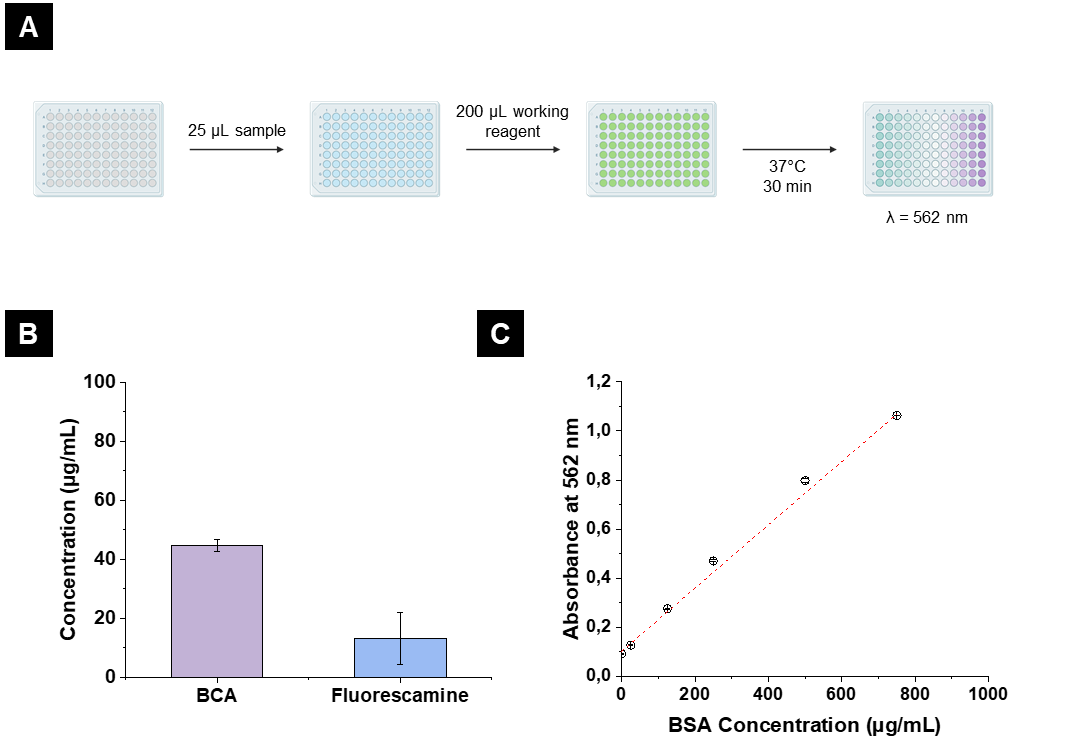


**Figure S7.** **Fluorescamine assay enables protein content measurement without influence of scattering.
A)** Schematic representation for the measurement of protein content by BCA assay. **B)** Comparison of the signal of liposomes control composed of DPPC:DSPE-PEG_2000_ (1%) at 10 mM in BCA and fluorescamine assay. **C)** Calibration curve used for BCA assay built by the absorbance at 562 nm versus the BSA concentration between 25 and 750 µg/mL. Data represented as the mean ± standard deviation of at least n=3.


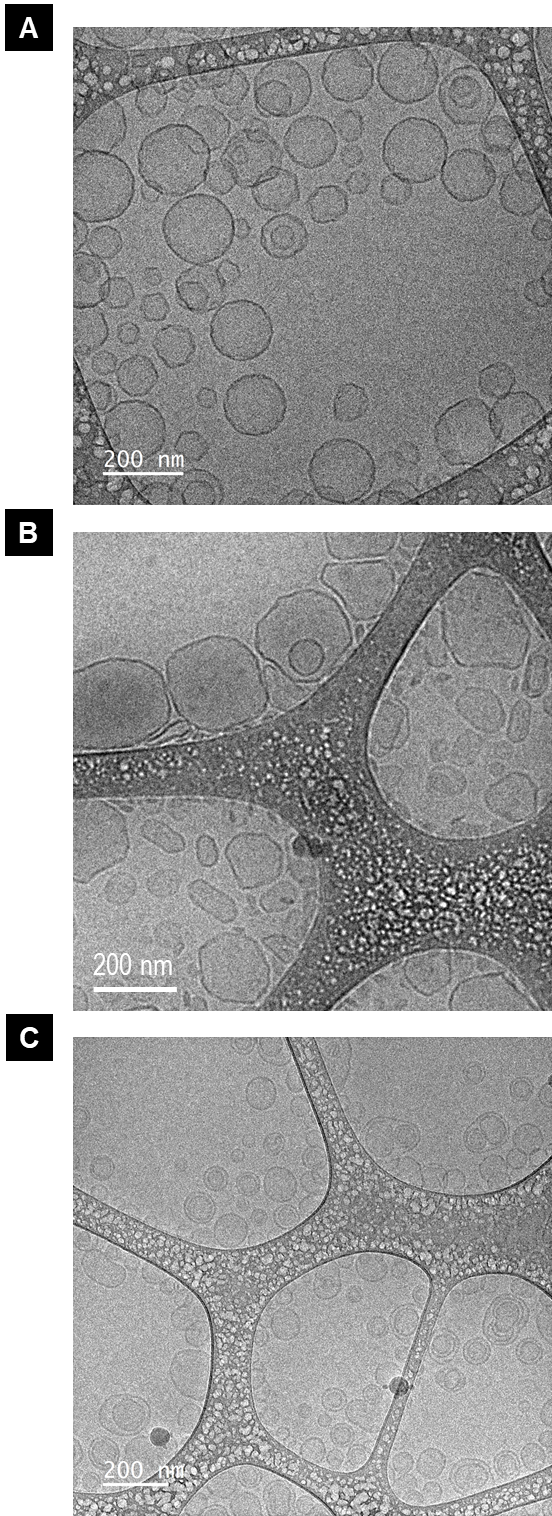


**Figure S8.** **Evaluation of the effect of temperature in the liposomes polygonal morphology.** CryoTEM micrographs of liposomes composed of A) DPPC:DSPE-PEG_2000_ (99:1 mol/mol) pre-warmed at 50ºC prior cryoplunging; B) DMPC:DSPE-PEG_2000_ (99:1 mol/mol) and C) DLPC:DSPE-PEG_2000_ (99:1 mol/mol). Both DMPC:DSPE-PEG_2000_ and DLPC:DSPE-PEG_2000_ were kept at 22ºC prior cryoplunging.


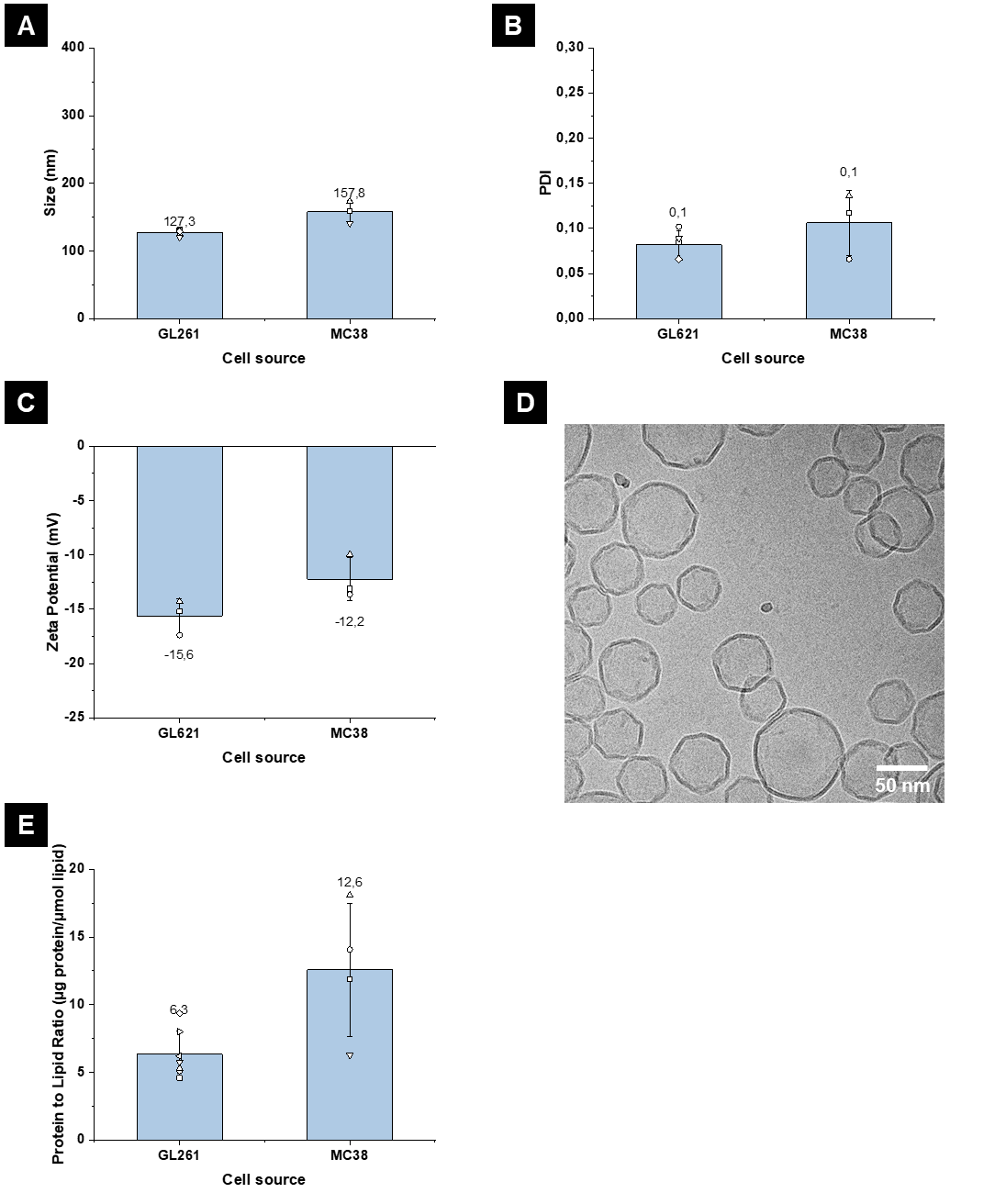


**Figure S9.** **Colorectal carcinoma (MC38) derived BV.** Description of the MC38-derived vesicles, by: **A)** mean hydrodynamic size, **B)** polydispersity index; **C)** zeta potential (compared to those from GL261); **D)** morphology in water by cryoEM; and **E)** protein to lipid ratio (compared to those from GL261).


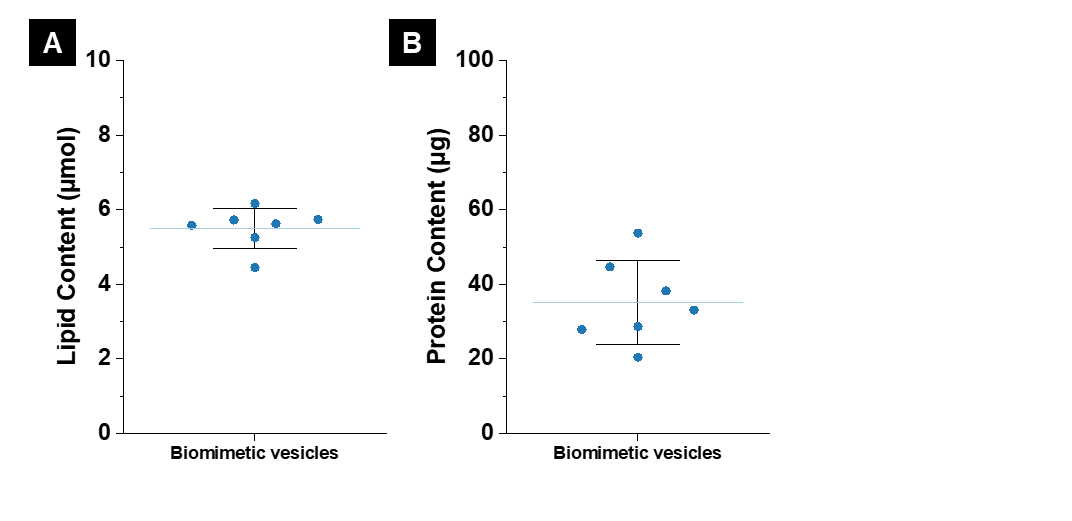


**Figure S10. BV molecular content quantification. A)** Lipid content quantification and **B)** Protein content quantification in BV composed of DPPC:DSPE-PEG_2000_ (1%) after concentration with Vivaspin 6 and prior proteomic analysis.


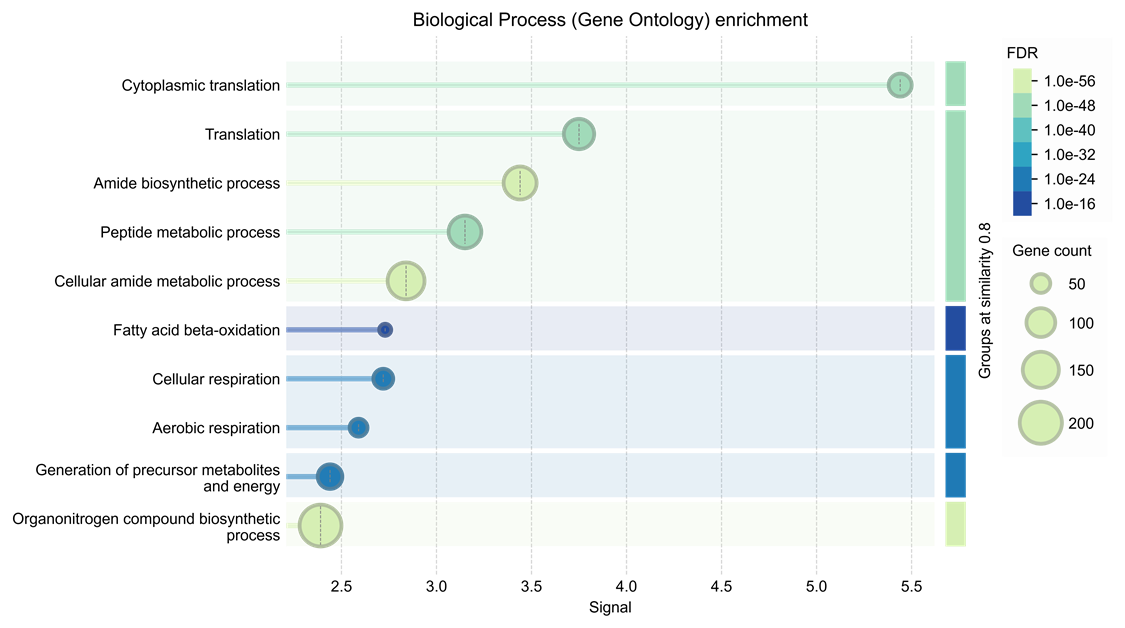


**Figure S11.** **Gene Ontology (GO) biological process enrichment analysis of proteins identified in the BV.** Bubble plot showing the most significantly enriched biological processes, with bubble size proportional to gene count and bubble color corresponding to false discovery rate (FDR). Major enriched categories include metabolic pathways, protein synthesis machinery, mitochondrial respiration, lipid metabolism, and vesicle/ER–Golgi transport processes. This enrichment pattern supports the presence of functionally diverse membrane-associated and intracellular proteins in the BV.
