# Supplementary material for: Engineering of Glioblastoma‐Derived Biomimetic Vesicles and Their Structural and Molecular Features
Source: Adv Healthc Mater. 2026 May 8;15(23):e03775. doi: 10.1002/adhm.202503775 (PMC13280188; doi:10.1002/adhm.202503775)
Supplement: Supplementary file 2 — Supporting File 2: adhm71222‐sup‐0002‐TableS1.docx. [file ADHM-15-0-s004.docx]

**Supporting Table 1.**
**Full list of identified proteins both in protein extract and BV**. For each protein, the protein name, encoded gene name, molecular weight, relative abundance (RA), standard deviation (SD) and cumulative value (CV) are specified.

|  |  |  | **Protein extract** | | | **BV** | | |
| --- | --- | --- | --- | --- | --- | --- | --- | --- |
| **Protein Name** | **Gene Name** | **MW (kDa)** | **RA** | **SD** | **CV** | **RA** | **SD** | **CV** |
| Cluster of Filamin, alpha | Flna | 280 | 0,2387 | 0,0098 | 4,0856 | 0,1563 | 0,0281 | 17,9442 |
| 60 kDa heat shock protein | Hspd1 | 61 | 0,8400 | 0,0519 | 6,1725 | 0,9299 | 0,0552 | 5,9344 |
| Endoplasmin | Hsp90b1 | 92 | 0,5499 | 0,0213 | 3,8678 | 0,5962 | 0,0433 | 7,2669 |
| Cluster of Tubulin beta chain | Tubb2b | 50 | 0,9003 | 0,0239 | 2,6546 | 1,2400 | 0,1272 | 10,2574 |
| 78 kDa glucose-regulated | Hspa5 | 72 | 0,6427 | 0,0195 | 3,0346 | 0,6496 | 0,0601 | 9,2592 |
| Cluster of Actin, beta | Actb | 42 | 0,8368 | 0,0229 | 2,7310 | 1,3727 | 0,2099 | 15,2926 |
| Cluster of Heat shock cognate 71 kDa | Hspa8 | 71 | 0,5034 | 0,0087 | 1,7356 | 0,6927 | 0,0625 | 9,0214 |
| Cluster of ATP synthase subunit alpha, mitochondrial | Atp5f1a | 60 | 0,5266 | 0,0340 | 6,4512 | 0,8088 | 0,1187 | 14,6790 |
| Cluster of Putative gag-pol protein | gag-pol | 120 | 0,2595 | 0,0039 | 1,5052 | 0,3668 | 0,0520 | 14,1846 |
| Heat shock protein HSP 90-beta | Hsp90ab1 | 83 | 0,3727 | 0,0139 | 3,7362 | 0,4394 | 0,0272 | 6,1791 |
| Protein disulfide-isomerase A3 | Pdia3 | 57 | 0,6068 | 0,0324 | 5,3439 | 0,5279 | 0,0522 | 9,8854 |
| Stress-70 protein, mitochondrial | Hspa9 | 73 | 0,4153 | 0,0267 | 6,4208 | 0,4688 | 0,0722 | 15,4035 |
| ATP synthase subunit beta | Atp5f1b | 56 | 0,4823 | 0,0243 | 5,0418 | 0,6999 | 0,0812 | 11,5982 |
| Malate dehydrogenase, mitochondrial | Mdh2 | 36 | 0,7399 | 0,0328 | 4,4329 | 0,9812 | 0,0629 | 6,4087 |
| Cluster of Tubulin alpha-1B chain | Tuba1b | 50 | 0,5145 | 0,0149 | 2,8865 | 0,6828 | 0,0776 | 11,3663 |
| Neutral alpha-glucosidase AB | Ganab | 108 | 0,2087 | 0,0030 | 1,4480 | 0,3018 | 0,0143 | 4,7524 |
| Clathrin heavy chain 1 | Cltc | 192 | 0,1158 | 0,0025 | 2,1197 | 0,1657 | 0,0117 | 7,0477 |
| Protein disulfide-isomerase | P4hb | 57 | 0,5984 | 0,0236 | 3,9384 | 0,1565 | 0,0166 | 10,5804 |
| Hypoxia up-regulated protein | Hyou1 | 111 | 0,1975 | 0,0096 | 4,8581 | 0,2605 | 0,0089 | 3,4166 |
| Cluster of Ras-related protein | Rab1A | 23 | 0,9323 | 0,0620 | 6,6484 | 1,1712 | 0,0894 | 7,6352 |
| Aconitate hydratase, mitochondrial | Aco2 | 85 | 0,2262 | 0,0102 | 4,5169 | 0,3281 | 0,0188 | 5,7231 |
| Calreticulin | Calr | 48 | 0,4666 | 0,0148 | 3,1691 | 0,4440 | 0,0985 | 22,1861 |
| Elongation factor 1-alpha | Eef1a1 | 50 | 0,4168 | 0,0313 | 7,5114 | 0,4160 | 0,0332 | 7,9848 |
| Cluster of Keratin, type II cytoskeletal | Krt6a | 59 | 0,2380 | 0,1256 | 52,7663 | 0,5303 | 0,1853 | 34,9351 |
| Aspartate aminotransferase, mitochondrial | Got2 | 47 | 0,3637 | 0,0204 | 5,6078 | 0,5538 | 0,0429 | 7,7423 |
| Heat shock protein HSP 90-alpha | Hsp90aa1 | 85 | 0,2253 | 0,0194 | 8,6095 | 0,2582 | 0,0291 | 11,2660 |
| Protein disulfide-isomerase A4 | Pdia4 | 72 | 0,3673 | 0,0347 | 9,4342 | 0,0997 | 0,0097 | 9,7741 |
| Elongation factor 2 | Eef2 | 95 | 0,2046 | 0,0121 | 5,9165 | 0,1945 | 0,0201 | 10,3264 |
| Cluster of Reticulon-4 | Rtn4 | 128 | 0,1267 | 0,0120 | 9,4898 | 0,1885 | 0,0126 | 6,6638 |
| Voltage-dependent anion-selective channel | Vdac1 | 32 | 0,4862 | 0,0124 | 2,5550 | 0,7696 | 0,0989 | 12,8548 |
| Cluster of Ornithine aminotransferase | Oat | 48 | 0,3207 | 0,0381 | 11,8694 | 0,4888 | 0,0633 | 12,9479 |
| Cluster of ADP/ATP translocase | Slc25a5 | 33 | 0,5090 | 0,0413 | 8,1206 | 0,5952 | 0,0619 | 10,3970 |
| Cluster of Plectin | Plec | 500 | 0,0382 | 0,0032 | 8,4233 | 0,0295 | 0,0103 | 34,7965 |
| Calnexin | Canx | 67 | 0,2172 | 0,0155 | 7,1186 | 0,3322 | 0,0419 | 12,5982 |
| Cluster of Sodium/potassium-transporting ATPase subunit alpha | Atp1a1 | 113 | 0,1317 | 0,0022 | 1,6468 | 0,1890 | 0,0126 | 6,6814 |
| Dolichyl-diphosphooligosaccharide--protein glycosyltransferase subunit | Rpn1 | 68 | 0,2309 | 0,0135 | 5,8293 | 0,2663 | 0,0181 | 6,7842 |
| Vimentin | Vim | 54 | 0,3150 | 0,0093 | 2,9673 | 0,2654 | 0,0521 | 19,6351 |
| Transitional endoplasmic reticulum ATPase | Vcp | 89 | 0,1847 | 0,0098 | 5,2891 | 0,1529 | 0,0177 | 11,5872 |
| Integrin beta-1 | Itgb1 | 88 | 0,1593 | 0,0079 | 4,9374 | 0,2024 | 0,0130 | 6,4024 |
| Mitochondrial 10-formyltetrahydrofolate dehydrogenase | Aldh1l2 | 102 | 0,1400 | 0,0128 | 9,1152 | 0,1716 | 0,0190 | 11,0749 |
| Sarcoplasmic/endoplasmic reticulum calcium ATPase | Atp2a2 | 116 | 0,1130 | 0,0075 | 6,5905 | 0,1654 | 0,0162 | 9,8148 |
| UDP-glucose:glycoprotein glucosyltransferase | Uggt1 | 177 | 0,0973 | 0,0066 | 6,7631 | 0,0620 | 0,0061 | 9,8339 |
| Monofunctional C1-tetrahydrofolate synthase | Mthfd1l | 107 | 0,1276 | 0,0052 | 4,0560 | 0,1621 | 0,0116 | 7,1889 |
| NAD(P) transhydrogenase, mitochondrial | Nnt | 114 | 0,1125 | 0,0013 | 1,1794 | 0,1600 | 0,0061 | 3,8090 |
| Staphylococcal nuclease domain-containing | Snd1 | 102 | 0,1315 | 0,0069 | 5,2758 | 0,1531 | 0,0127 | 8,2757 |
| Alanine aminotransferase 2 | Gpt2 | 58 | 0,2344 | 0,0163 | 6,9428 | 0,2555 | 0,0333 | 13,0340 |
| T-complex protein 1 subunit | Cct4 | 58 | 0,2272 | 0,0195 | 8,5997 | 0,2582 | 0,0223 | 8,6518 |
| Heterogeneous nuclear ribonucleoprotein U | Hnrnpu | 88 | 0,1371 | 0,0023 | 1,6462 | 0,1915 | 0,0198 | 10,3605 |
| Chondroitin sulfate proteoglycan 4 | Cspg4 | 252 | 0,0495 | 0,0057 | 11,5054 | 0,0607 | 0,0151 | 24,8415 |
| Cluster of Keratin, type | Krt14 | 53 | 0,1748 | 0,1171 | 67,0103 | 0,3664 | 0,1000 | 27,2872 |
| Serpin H1 | Serpinh1 | 47 | 0,2981 | 0,0185 | 6,2015 | 0,2264 | 0,0059 | 2,5964 |
| Cluster of Integrin | Itga6 | 122 | 0,0932 | 0,0067 | 7,2393 | 0,1256 | 0,0200 | 15,9137 |
| Histidine kinase/HSP90-like ATPase domain-containing | Trap1 | 80 | 0,1409 | 0,0069 | 4,8745 | 0,1873 | 0,0289 | 15,4366 |
| Cytoskeleton-associated protein 4 | Ckap4 | 64 | 0,1816 | 0,0054 | 2,9478 | 0,2205 | 0,0091 | 4,1338 |
| DBH-like monooxygenase protein | Moxd1 | 70 | 0,1627 | 0,0099 | 6,0839 | 0,2022 | 0,0144 | 7,1004 |
| Glutamate dehydrogenase 1, mitochondrial | Glud1 | 61 | 0,1625 | 0,0089 | 5,4616 | 0,2703 | 0,0162 | 5,9998 |
| Protein disulfide-isomerase A6 | Pdia6 | 49 | 0,3127 | 0,0117 | 3,7508 | 0,1442 | 0,0518 | 35,9115 |
| Prohibitin-2 | Phb2 | 33 | 0,2959 | 0,0177 | 5,9867 | 0,4942 | 0,0362 | 7,3155 |
| Large ribosomal subunit protein | Rpl5 | 34 | 0,3117 | 0,0068 | 2,1792 | 0,4130 | 0,0493 | 11,9456 |
| Citrate synthase, mitochondrial | Cs | 52 | 0,2091 | 0,0131 | 6,2474 | 0,2721 | 0,0463 | 17,0064 |
| Cluster of Large ribosomal subunit protein | Rpl3 | 46 | 0,2338 | 0,0176 | 7,5115 | 0,3049 | 0,0351 | 11,5111 |
| Cluster of Aldehyde dehydrogenase | Aldh2 | 57 | 0,1745 | 0,0179 | 10,2661 | 0,2717 | 0,0440 | 16,1971 |
| Large ribosomal subunit protein | Rpl4 | 47 | 0,2215 | 0,0162 | 7,3277 | 0,3040 | 0,0219 | 7,2204 |
| Large ribosomal subunit protein | Rpl7 | 31 | 0,3288 | 0,0292 | 8,8834 | 0,4615 | 0,0585 | 12,6652 |
| Pyruvate dehydrogenase E1 component subunit alpha, somatic form | Pdha1 | 43 | 0,2425 | 0,0070 | 2,8727 | 0,3027 | 0,0398 | 13,1553 |
| Cluster of Ras-related protein Rab | Rab2a | 24 | 0,4099 | 0,0437 | 10,6554 | 0,5652 | 0,0360 | 6,3709 |
| Glyceraldehyde-3-phosphate dehydrogenase | Gapdh | 36 | 0,2850 | 0,0071 | 2,4947 | 0,3485 | 0,0455 | 13,0439 |
| Dehydrogenase/reductase SDR family | Dhrs1 | 34 | 0,2773 | 0,0192 | 6,9288 | 0,4224 | 0,0640 | 15,1516 |
| Cytochrome b-c1 complex subunit | Uqcrc2 | 48 | 0,1979 | 0,0170 | 8,5795 | 0,2899 | 0,0320 | 11,0497 |
| Env polyprotein | Mela | 74 | 0,1365 | 0,0085 | 6,2419 | 0,1617 | 0,0114 | 7,0673 |
| Delta-1-pyrroline-5-carboxylate synthase | Aldh18a1 | 87 | 0,1241 | 0,0132 | 10,6118 | 0,1212 | 0,0204 | 16,8449 |
| Glycerol-3-phosphate dehydrogenase | Gpd2 | 83 | 0,1261 | 0,0128 | 10,1662 | 0,1306 | 0,0264 | 20,1894 |
| Transferrin receptor protein 1 | Tfrc | 86 | 0,1142 | 0,0063 | 5,4819 | 0,1309 | 0,0395 | 30,1717 |
| T-complex protein 1 subunit | Cct8 | 60 | 0,1523 | 0,0094 | 6,1539 | 0,2113 | 0,0388 | 18,3607 |
| Dihydropyrimidinase-related protein 2 | Dpysl2 | 62 | 0,1419 | 0,0062 | 4,3831 | 0,2042 | 0,0225 | 11,0354 |
| Dihydrolipoyl dehydrogenase, mitochondrial | Dld | 54 | 0,1854 | 0,0115 | 6,1968 | 0,1895 | 0,0256 | 13,4849 |
| D-3-phosphoglycerate dehydrogenase | Phgdh | 57 | 0,1471 | 0,0070 | 4,7354 | 0,2278 | 0,0202 | 8,8754 |
| Isocitrate dehydrogenase [NADP], mitochondrial | Idh2 | 51 | 0,1592 | 0,0158 | 9,8919 | 0,2581 | 0,0294 | 11,3787 |
| T-complex protein 1 subunit | Cct3 | 61 | 0,1550 | 0,0276 | 17,7900 | 0,1714 | 0,0382 | 22,2850 |
| ATP synthase subunit gamma | Atp5f1c | 30 | 0,2657 | 0,0297 | 11,1874 | 0,4493 | 0,0646 | 14,3686 |
| Protease, serine 59 (Fragment) | Prss59 | 20 | 0,4453 | 0,2944 | 66,1004 | 0,6134 | 0,3823 | 62,3337 |
| NADH-cytochrome b5 reductase | Cyb5r3 | 35 | 0,2516 | 0,0054 | 2,1352 | 0,3337 | 0,0549 | 16,4422 |
| Solute carrier family 25 member | Slc25a3 | 40 | 0,2220 | 0,0156 | 7,0456 | 0,2843 | 0,0285 | 10,0077 |
| phosphoenolpyruvate carboxykinase (GTP= | Pck2 | 73 | 0,1184 | 0,0041 | 3,4506 | 0,1555 | 0,0257 | 16,5338 |
| Cluster of Neural cell adhesion molecule L1-like | Chl1 | 136 | 0,0668 | 0,0088 | 13,1617 | 0,0792 | 0,0093 | 11,7804 |
| Trifunctional enzyme subunit alpha | Hadha | 83 | 0,1082 | 0,0014 | 1,3152 | 0,1277 | 0,0104 | 8,1332 |
| Succinate dehydrogenase [ubiquinone] flavoprotein subunit | Sdha | 73 | 0,1311 | 0,0109 | 8,3241 | 0,1285 | 0,0235 | 18,3110 |
| Cluster of RNA helicase | Ddx39a | 49 | 0,1954 | 0,0055 | 2,8139 | 0,1967 | 0,0253 | 12,8873 |
| Coatomer subunit alpha | Copa | 139 | 0,0667 | 0,0126 | 18,8754 | 0,0646 | 0,0210 | 32,5403 |
| Cluster of Fructose-bisphosphate | Aldoa | 39 | 0,3372 | 0,0327 | 9,6940 | 0,0577 | 0,0164 | 28,3997 |
| Catenin (cadherin associated protein) | Ctnnd1 | 108 | 0,0758 | 0,0036 | 4,7664 | 0,0981 | 0,0118 | 12,0474 |
| T-complex protein 1 subunit | Cct2 | 57 | 0,1470 | 0,0090 | 6,1394 | 0,1796 | 0,0126 | 7,0251 |
| BOS complex subunit NOMO1 | Nomo1 | 133 | 0,0674 | 0,0098 | 14,5322 | 0,0685 | 0,0073 | 10,6861 |
| T-complex protein 1 subunit | Cct5 | 60 | 0,1415 | 0,0125 | 8,8151 | 0,1645 | 0,0069 | 4,2123 |
| T-complex protein 1 subunit | Cct7 | 60 | 0,1397 | 0,0181 | 12,9270 | 0,1539 | 0,0261 | 16,9653 |
| Uncharacterized protein | Vdac3 | 31 | 0,2349 | 0,0259 | 11,0115 | 0,3677 | 0,0701 | 19,0568 |
| Cluster of 10 kDa heat shock protein | Hspe1 | 11 | 0,9794 | 0,0140 | 1,4260 | 0,4875 | 0,1628 | 33,3892 |
| Cytochrome b-c1 complex subunit | Uqcrc1 | 53 | 0,1437 | 0,0043 | 2,9763 | 0,2035 | 0,0143 | 7,0095 |
| Probable ATP-dependent | Ddx5 | 69 | 0,1240 | 0,0061 | 4,9146 | 0,1260 | 0,0225 | 17,8461 |
| Cluster of Erythrocyte protein band 4.1-like | Epb41l2 | 110 | 0,0821 | 0,0154 | 18,7629 | 0,0708 | 0,0241 | 34,1070 |
| Cluster of procollagen-proline | P4ha1 | 61 | 0,1321 | 0,0081 | 6,1641 | 0,1613 | 0,0141 | 8,7338 |
| L1 cell adhesion molecule | L1cam | 140 | 0,0552 | 0,0081 | 14,7167 | 0,0741 | 0,0111 | 15,0169 |
| Cluster of Actinin | Actn4 | 108 | 0,1228 | 0,0107 | 8,7255 | 0,0081 | 0,0020 | 24,4685 |
| Cluster of Nucleolin | Ncl | 77 | 0,1678 | 0,0096 | 5,7160 | 0,0163 | 0,0100 | 60,9723 |
| procollagen-lysine 5-dioxygenase | Plod2 | 87 | 0,0975 | 0,0122 | 12,4923 | 0,0986 | 0,0074 | 7,5133 |
| Peroxisomal multifunctional enzyme type | Hsd17b4 | 79 | 0,1050 | 0,0095 | 9,0519 | 0,1138 | 0,0111 | 9,7148 |
| Cluster of ATP synthase subunit O, mitochondrial | Atp5po | 23 | 0,3464 | 0,0174 | 5,0228 | 0,4100 | 0,0331 | 8,0650 |
| Stress-induced-phosphoprotein 1 | Stip1 | 63 | 0,2027 | 0,0269 | 13,2636 | 0,0160 | 0,0076 | 47,8143 |
| Cluster of Alpha-enolase | Eno1 | 47 | 0,1782 | 0,0137 | 7,6685 | 0,1835 | 0,0233 | 12,6807 |
| Cluster of Envelope |  | 64 | 0,1361 | 0,0061 | 4,5084 | 0,1190 | 0,0140 | 11,8079 |
| Keratin 10 | Krt10 | 57 | 0,1135 | 0,0420 | 37,0007 | 0,2013 | 0,0453 | 22,5268 |
| Cluster of Integrin | Itgav | 112 | 0,0678 | 0,0045 | 6,6756 | 0,0845 | 0,0157 | 18,5504 |
| NAD-dependent malic enzyme | Me2 | 66 | 0,1217 | 0,0054 | 4,4675 | 0,1310 | 0,0033 | 2,4933 |
| Peptidyl-prolyl cis-trans | Fkbp9 | 63 | 0,1476 | 0,0160 | 10,8097 | 0,1030 | 0,0065 | 6,3563 |
| Isocitrate dehydrogenase [NAD] subunit | Idh3a | 42 | 0,1813 | 0,0105 | 5,7996 | 0,2185 | 0,0312 | 14,2611 |
| C-1-tetrahydrofolate synthase | Mthfd1 | 101 | 0,0795 | 0,0047 | 5,9620 | 0,0825 | 0,0100 | 12,1097 |
| Large ribosomal subunit protein | Rpl8 | 28 | 0,2721 | 0,0076 | 2,7840 | 0,3226 | 0,0669 | 20,7298 |
| Ribosome binding protein 1 | Rrbp1 | 159 | 0,0609 | 0,0083 | 13,5947 | 0,0298 | 0,0205 | 68,8419 |
| Long-chain specific acyl-CoA dehydrogenase | Acadl | 48 | 0,1406 | 0,0156 | 11,1244 | 0,2130 | 0,0281 | 13,2068 |
| Large ribosomal subunit protein | Rplp0 | 34 | 0,2291 | 0,0136 | 5,9290 | 0,2461 | 0,0330 | 13,4131 |
| Keratin, type I cytoskeletal | Krt42 | 50 | 0,1048 | 0,0716 | 68,2863 | 0,2412 | 0,0620 | 25,6861 |
| Cluster of Neural cell adhesion | Ncam1 | 93 | 0,0690 | 0,0035 | 5,0993 | 0,1117 | 0,0141 | 12,6422 |
| Cluster of Phosphoglycerate | Pgk1 | 45 | 0,1710 | 0,0035 | 2,0536 | 0,1780 | 0,0209 | 11,7204 |
| Large ribosomal subunit protein | Rpl7a | 30 | 0,2254 | 0,0147 | 6,5059 | 0,3312 | 0,0401 | 12,1112 |
| CD109 antigen | Cd109 | 162 | 0,0481 | 0,0039 | 8,1737 | 0,0489 | 0,0076 | 15,5332 |
| T-complex protein 1 subunit | Tcp1 | 60 | 0,1143 | 0,0086 | 7,5256 | 0,1563 | 0,0209 | 13,3577 |
| Elongation factor G, mitochondrial | Gfm1 | 84 | 0,0879 | 0,0106 | 12,0085 | 0,1019 | 0,0057 | 5,5709 |
| RNA helicase | Ddx3x | 73 | 0,0951 | 0,0150 | 15,8085 | 0,1239 | 0,0072 | 5,7792 |
| Cluster of T-complex protein 1 subunit zeta | Cct6a | 58 | 0,1181 | 0,0113 | 9,6106 | 0,1595 | 0,0145 | 9,0994 |
| Pyruvate dehydrogenase E1 component subunit beta | Pdhb | 39 | 0,1869 | 0,0158 | 8,4601 | 0,2098 | 0,0117 | 5,5539 |
| Cluster of Calumenin OS | Calu | 37 | 0,2058 | 0,0228 | 11,0574 | 0,2002 | 0,0230 | 11,5065 |
| Procollagen-lysine,2-oxoglutarate | Plod1 | 84 | 0,0793 | 0,0109 | 13,7125 | 0,1064 | 0,0141 | 13,2373 |
| Lon protease homolog, mitochondrial | Lonp1 | 107 | 0,0677 | 0,0052 | 7,6967 | 0,0722 | 0,0123 | 16,9688 |
| Electron transfer flavoprotein subunit | Etfb | 28 | 0,2389 | 0,0280 | 11,7354 | 0,3216 | 0,0686 | 21,3303 |
| Peroxiredoxin-4 | Prdx4 | 31 | 0,2580 | 0,0401 | 15,5551 | 0,2030 | 0,0023 | 1,1412 |
| Presequence protease, mitochondrial | Pitrm1 | 118 | 0,0625 | 0,0098 | 15,7408 | 0,0618 | 0,0161 | 26,1082 |
| Cluster of Catenin | Ctnna1 | 100 | 0,0794 | 0,0061 | 7,6958 | 0,0602 | 0,0157 | 26,0466 |
| Ras-related protein Rab | Rab7a | 23 | 0,2794 | 0,0208 | 7,4541 | 0,3972 | 0,0652 | 16,4147 |
| Succinyl-CoA:3-ketoacid-coenzyme | Oxct1 | 56 | 0,1178 | 0,0076 | 6,4505 | 0,1578 | 0,0230 | 14,5516 |
| Elongation factor Tu, mitochondrial | Tufm | 50 | 0,1431 | 0,0212 | 14,7953 | 0,1517 | 0,0206 | 13,5707 |
| Glucosidase 2 subunit beta | Prkcsh | 59 | 0,1507 | 0,0117 | 7,7532 | 0,0784 | 0,0262 | 33,4242 |
| Small ribosomal subunit protein | Rps3 | 27 | 0,2438 | 0,0056 | 2,2895 | 0,3056 | 0,0295 | 9,6698 |
| 2-oxoglutarate dehydrogenase complex | Ogdh | 117 | 0,0539 | 0,0040 | 7,3583 | 0,0786 | 0,0119 | 15,1744 |
| Leucine-rich repeat-containing | Lrrc59 | 35 | 0,1909 | 0,0147 | 7,7131 | 0,2325 | 0,0266 | 11,4258 |
| Transmembrane emp24 domain-containing | Tmed10 | 25 | 0,2567 | 0,0276 | 10,7709 | 0,3409 | 0,0297 | 8,7112 |
| Leucine-rich PPR motif-containing protein | Lrpprc | 158 | 0,0489 | 0,0039 | 8,0668 | 0,0385 | 0,0130 | 33,9094 |
| Cluster of Glutaminase kidney isoform | Gls | 74 | 0,0947 | 0,0089 | 9,4414 | 0,1000 | 0,0073 | 7,2588 |
| Cluster of Ras-related protein Rab | Rab5c | 23 | 0,2786 | 0,0105 | 3,7586 | 0,3710 | 0,0581 | 15,6681 |
| procollagen-proline 3-dioxygenase | P3h1 | 84 | 0,0786 | 0,0092 | 11,7268 | 0,0939 | 0,0107 | 11,3741 |
| Ras-related protein Rab | Rab6a | 24 | 0,2643 | 0,0222 | 8,4065 | 0,3559 | 0,0534 | 14,9998 |
| Vesicle-associated membrane protein-associated | Vapa | 28 | 0,2166 | 0,0128 | 5,9216 | 0,3212 | 0,0513 | 15,9791 |
| Adipocyte plasma membrane-associated | Apmap | 46 | 0,1339 | 0,0085 | 6,3829 | 0,1779 | 0,0210 | 11,8243 |
| Polyadenylate-binding protein 1 | Pabpc1 | 71 | 0,1445 | 0,0097 | 6,6977 | 0,0187 | 0,0026 | 13,7199 |
| Cluster of Catenin | Ctnnb1 | 85 | 0,0840 | 0,0146 | 17,3984 | 0,0746 | 0,0177 | 23,6917 |
| Proliferation-associated protein 2G4 | Pa2g4 | 44 | 0,1487 | 0,0285 | 19,1348 | 0,1707 | 0,0324 | 19,0035 |
| Large ribosomal subunit protein | Rpl6 | 34 | 0,1865 | 0,0281 | 15,0474 | 0,2237 | 0,0129 | 5,7614 |
| Elongation factor 1-gamma | Eef1g | 50 | 0,1280 | 0,0149 | 11,6320 | 0,1528 | 0,0267 | 17,5047 |
| Ubiquitin-ribosomal protein eS31 fusion protein | Rps27a | 18 | 0,3284 | 0,0250 | 7,6016 | 0,4567 | 0,0729 | 15,9588 |
| Integrin alpha-1 | Itga1 | 131 | 0,0483 | 0,0113 | 23,4128 | 0,0562 | 0,0079 | 13,9667 |
| Emerin | Emd | 29 | 0,1913 | 0,0196 | 10,2489 | 0,2954 | 0,0245 | 8,3060 |
| Cluster of Heat shock 105kDa/110kDa | Hsph1 | 92 | 0,0748 | 0,0140 | 18,7606 | 0,0649 | 0,0197 | 30,4173 |
| Dolichyl-diphosphooligosaccharide--protein glycosyltransferase | Rpn2 | 68 | 0,0868 | 0,0030 | 3,4926 | 0,1139 | 0,0141 | 12,3847 |
| Ribosomal protein 10 | RP23-436K3 | 25 | 0,2500 | 0,0252 | 10,0755 | 0,2773 | 0,0503 | 18,1489 |
| Large ribosomal subunit protein | Rpl13a | 23 | 0,2424 | 0,0280 | 11,5617 | 0,3752 | 0,0814 | 21,6944 |
| L-type lectin-like domain-containing | Lman1 | 61 | 0,0982 | 0,0060 | 6,1223 | 0,1295 | 0,0367 | 28,3586 |
| Cell surface glycoprotein MUC18 | Mcam | 72 | 0,0761 | 0,0050 | 6,6243 | 0,1206 | 0,0194 | 16,0581 |
| Ribosomal protein | Rpl10a | 25 | 0,2293 | 0,0100 | 4,3559 | 0,3164 | 0,0341 | 10,7763 |
| Nucleophosmin | Npm1 | 33 | 0,2610 | 0,0235 | 8,9932 | 0,0887 | 0,0192 | 21,6256 |
| Voltage-dependent anion channel | Vdac2 | 30 | 0,1799 | 0,0104 | 5,7826 | 0,2745 | 0,0464 | 16,9096 |
| Extended synaptotagmin-1 | Esyt1 | 122 | 0,0520 | 0,0033 | 6,2791 | 0,0507 | 0,0111 | 21,9721 |
| Bifunctional phosphoribosylaminoimidazole carboxylase/phosphoribosylaminoimidazole succinocarboxamide | Paics | 47 | 0,1239 | 0,0058 | 4,6620 | 0,1526 | 0,0276 | 18,1002 |
| Cluster of Rps16 protein | Rps16 | 16 | 0,3371 | 0,0558 | 16,5457 | 0,4976 | 0,0320 | 6,4337 |
| Large ribosomal subunit protein | Rpl18a | 21 | 0,2704 | 0,0483 | 17,8632 | 0,3558 | 0,0533 | 14,9812 |
| Irgm1 | Irgm1 | 47 | 0,1217 | 0,0116 | 9,5160 | 0,1545 | 0,0358 | 23,1620 |
| Electron transfer flavoprotein subunit alpha | Etfa | 35 | 0,1610 | 0,0205 | 12,7170 | 0,2067 | 0,0145 | 7,0154 |
| AHNAK nucleoprotein (desmoyokin | Ahnak | 604 | 0,0163 | 0,0019 | 11,6092 | 0,0006 | 0,0007 | 120,7865 |
| Cluster of Amino acid transporter heavy chain | Slc3a2 | 58 | 0,0986 | 0,0136 | 13,7852 | 0,1163 | 0,0229 | 19,7038 |
| Large ribosomal subunit protein | Rpl12 | 18 | 0,3189 | 0,0181 | 5,6724 | 0,3746 | 0,0169 | 4,5210 |
| Large ribosomal subunit protein | Rpl26 | 17 | 0,3276 | 0,0346 | 10,5601 | 0,4181 | 0,0939 | 22,4527 |
| Annexin A2 | Anxa2 | 39 | 0,1888 | 0,0178 | 9,4365 | 0,0945 | 0,0240 | 25,3639 |
| peptidylprolyl isomerase | Fkbp10 | 65 | 0,1330 | 0,0065 | 4,8729 | 0,0249 | 0,0071 | 28,6260 |
| Keratin 16 | Krt16 | 52 | 0,0662 | 0,0473 | 71,4631 | 0,1884 | 0,0815 | 43,2496 |
| Peptidyl-prolyl cis-trans | Ppib | 24 | 0,3239 | 0,0305 | 9,4094 | 0,1258 | 0,0300 | 23,8678 |
| Cluster of Guanine nucleotide binding protein (G protein | Gnb2 | 41 | 0,1340 | 0,0228 | 17,0053 | 0,1649 | 0,0206 | 12,4898 |
| Guanine nucleotide-binding protein G(i) subunit | Gnai2 | 40 | 0,1346 | 0,0069 | 5,1179 | 0,1737 | 0,0359 | 20,6630 |
| Ras-related protein Rab | Rab10 | 23 | 0,2346 | 0,0215 | 9,1856 | 0,2960 | 0,0302 | 10,2024 |
| hexokinase | Hk1 | 102 | 0,0503 | 0,0118 | 23,4361 | 0,0677 | 0,0064 | 9,4878 |
| Aspartate-beta-hydroxylase | Asph | 81 | 0,0653 | 0,0050 | 7,5969 | 0,0799 | 0,0161 | 20,2081 |
| Cluster of Heterogeneous nuclear | Hnrnph1 | 49 | 0,1220 | 0,0081 | 6,6719 | 0,1103 | 0,0149 | 13,5082 |
| Dolichyl-diphosphooligosaccharide--protein glycosyltransferase 48 kDa | Ddost | 49 | 0,1028 | 0,0074 | 7,1946 | 0,1455 | 0,0341 | 23,4133 |
| Large ribosomal subunit protein | Rpl30 | 13 | 0,3890 | 0,0343 | 8,8143 | 0,5456 | 0,0487 | 8,9343 |
| Cluster of Large ribosomal subunit protein | Rpl24 | 18 | 0,2525 | 0,0448 | 17,7225 | 0,4463 | 0,0623 | 13,9667 |
| polyribonucleotide nucleotidyltransferase | Pnpt1 | 86 | 0,0716 | 0,0027 | 3,7463 | 0,0583 | 0,0159 | 27,2391 |
| Histone H4 | Hist2h4 | 11 | 0,4091 | 0,0841 | 20,5537 | 0,7344 | 0,1886 | 25,6789 |
| Lanosterol 14-alpha demethylase | Cyp51a1 | 57 | 0,0915 | 0,0052 | 5,6385 | 0,1133 | 0,0102 | 9,0409 |
| Acetyl-CoA acetyltransferase, mitochondrial | Acat1 | 45 | 0,1182 | 0,0203 | 17,1765 | 0,1392 | 0,0220 | 15,7873 |
| Trifunctional enzyme subunit beta | Hadhb | 51 | 0,1108 | 0,0055 | 5,0084 | 0,1124 | 0,0090 | 8,0152 |
| CAAX prenyl protease | Zmpste24 | 55 | 0,0872 | 0,0063 | 7,1702 | 0,1295 | 0,0178 | 13,7420 |
| Cluster of Large ribosomal subunit protein | Rpl17 | 21 | 0,2485 | 0,0134 | 5,4004 | 0,2989 | 0,0388 | 12,9870 |
| Large ribosomal subunit protein | Rpl14 | 24 | 0,2159 | 0,0477 | 22,0761 | 0,2571 | 0,0440 | 17,0948 |
| Peptidyl-prolyl cis-trans | Ppia | 18 | 0,4342 | 0,0764 | 17,5886 | 0,1077 | 0,0676 | 62,7869 |
| Large ribosomal subunit protein | Rpl13 | 24 | 0,1852 | 0,0052 | 2,8114 | 0,3240 | 0,0919 | 28,3468 |
| Guanine nucleotide-binding protein G(I)/G(S)/G(T) subunit | Gnb1 | 37 | 0,1272 | 0,0133 | 10,4247 | 0,1904 | 0,0085 | 4,4672 |
| Small ribosomal subunit protein | Rack1 | 35 | 0,1416 | 0,0163 | 11,5008 | 0,1865 | 0,0200 | 10,7187 |
| Protein RCC2 | Rcc2 | 56 | 0,0947 | 0,0064 | 6,7884 | 0,1037 | 0,0133 | 12,7935 |
| protein disulfide-isomerase | Txndc5 | 39 | 0,2063 | 0,0153 | 7,4046 | 0,0267 | 0,0153 | 57,4398 |
| Serine/arginine-rich splicing | Srsf1 | 28 | 0,3055 | 0,0107 | 3,5071 | 0,0040 | 0,0079 | 200,0000 |
| ATP synthase subunit b | Atp5pb | 29 | 0,1566 | 0,0110 | 7,0381 | 0,2426 | 0,0331 | 13,6418 |
| Tissue factor | F3 | 33 | 0,1451 | 0,0160 | 11,0238 | 0,1917 | 0,0313 | 16,3270 |
| Isocitrate dehydrogenase [NAD] subunit | Idh3b | 42 | 0,1100 | 0,0091 | 8,3055 | 0,1590 | 0,0148 | 9,2950 |
| protein-synthesizing GTPase | Eif2s3x | 51 | 0,1155 | 0,0186 | 16,0663 | 0,0813 | 0,0275 | 33,8383 |
| Small ribosomal subunit protein | Rps9 | 23 | 0,2009 | 0,0146 | 7,2822 | 0,2762 | 0,0242 | 8,7640 |
| RAS oncogene family protein | Rab11b | 24 | 0,1816 | 0,0053 | 2,9336 | 0,2872 | 0,0432 | 15,0466 |
| Acyl-coenzyme A thioesterase | Acot9 | 51 | 0,1107 | 0,0162 | 14,5875 | 0,0869 | 0,0125 | 14,3948 |
| Desmoplakin | Dsp | 333 | 0,0065 | 0,0083 | 128,0181 | 0,0283 | 0,0221 | 78,1051 |
| Large ribosomal subunit protein | Rpl18 | 22 | 0,1869 | 0,0166 | 8,8844 | 0,3256 | 0,0491 | 15,0856 |
| Pyruvate kinase PKM | Pkm | 58 | 0,0896 | 0,0110 | 12,3327 | 0,0865 | 0,0131 | 15,1008 |
| Cluster of Moesin OS | Msn | 68 | 0,1144 | 0,0109 | 9,5295 | 0,0111 | 0,0043 | 39,1752 |
| Pyrroline-5-carboxylate reductase | Pycr2 | 34 | 0,1465 | 0,0187 | 12,7421 | 0,1536 | 0,0355 | 23,1135 |
| Iron-sulfur clusters transporter | Abcb7 | 83 | 0,0597 | 0,0088 | 14,6855 | 0,0647 | 0,0056 | 8,7106 |
| Small ribosomal subunit protein | Rps3a | 30 | 0,1540 | 0,0190 | 12,3354 | 0,1954 | 0,0188 | 9,6355 |
| Multifunctional procollagen lysine hydroxylase and | Plod3 | 85 | 0,0463 | 0,0105 | 22,7209 | 0,0834 | 0,0172 | 20,6185 |
| Large ribosomal subunit protein | Rpl9 | 22 | 0,1984 | 0,0192 | 9,6592 | 0,2942 | 0,0877 | 29,8126 |
| Succinate--CoA ligase [ADP/GDP-forming] subunit alpha | Suclg1 | 36 | 0,1406 | 0,0140 | 9,9674 | 0,1361 | 0,0325 | 23,8850 |
| Lysyl oxidase homolog 3 | Loxl3 | 84 | 0,0589 | 0,0081 | 13,8240 | 0,0612 | 0,0068 | 11,1662 |
| Far upstream element (FUSE) binding | Fubp1 | 69 | 0,1030 | 0,0103 | 10,0449 | 0,0206 | 0,0077 | 37,2681 |
| Ribosomal protein L15 | Rpl15 | 24 | 0,1710 | 0,0071 | 4,1760 | 0,2692 | 0,0525 | 19,4849 |
| Laminin B1 | Lamb1 | 202 | 0,0300 | 0,0053 | 17,5129 | 0,0149 | 0,0036 | 24,3076 |
| Nucleobindin-1 | Nucb1 |  | 0,1479 | 0,0244 | 16,4649 | 0,0000 | 0,0000 |  |
| Guanine nucleotide-binding protein G(s) subunit alpha isoforms | Gnas | 46 | 0,0966 | 0,0087 | 9,0321 | 0,1257 | 0,0206 | 16,3732 |
| RNA-splicing ligase RtcB | Rtcb | 55 | 0,0792 | 0,0085 | 10,7083 | 0,1087 | 0,0358 | 32,9321 |
| Cytochrome c1, heme protein | Cyc1 | 35 | 0,1246 | 0,0061 | 4,8714 | 0,1662 | 0,0276 | 16,5862 |
| Guanine nucleotide-binding protein G(i) subunit | Gnai3 | 41 | 0,1103 | 0,0190 | 17,2211 | 0,1357 | 0,0231 | 17,0022 |
| Dihydrolipoyllysine-residue acetyltransferase component of pyruvate dehydrogenase complex | Dlat | 68 | 0,0727 | 0,0061 | 8,4297 | 0,0674 | 0,0103 | 15,2897 |
| Cluster of NADPH--cytochrome | Por | 77 | 0,0623 | 0,0092 | 14,7626 | 0,0585 | 0,0301 | 51,4229 |
| Cluster of Calponin OS | Cnn3 | 31 | 0,1904 | 0,0159 | 8,3741 | 0,0897 | 0,0288 | 32,1053 |
| Isocitrate dehydrogenase [NAD] subunit gamma 1, mitochondrial | Idh3g | 43 | 0,1051 | 0,0170 | 16,1459 | 0,1183 | 0,0188 | 15,9162 |
| 3-mercaptopyruvate sulfurtransferase | Mpst | 33 | 0,1243 | 0,0040 | 3,2001 | 0,1793 | 0,0255 | 14,2384 |
| Cluster of Histone H2B (Fragment) OS=Mus | Hist1h2bj | 14 | 0,3296 | 0,0471 | 14,2732 | 0,3518 | 0,0761 | 21,6435 |
| Cartilage-associated protein | Crtap | 46 | 0,0892 | 0,0142 | 15,9068 | 0,1223 | 0,0140 | 11,4709 |
| EF-hand domain-containing | Slc25a13 | 74 | 0,0645 | 0,0093 | 14,4023 | 0,0589 | 0,0141 | 23,8969 |
| Dihydropyrimidinase-related protein 3 | Dpysl3 | 62 | 0,0718 | 0,0082 | 11,4731 | 0,0828 | 0,0130 | 15,7134 |
| Vesicle-trafficking protein SEC22b | Sec22b | 25 | 0,1712 | 0,0084 | 4,8849 | 0,2070 | 0,0219 | 10,6037 |
| Medium-chain specific acyl-CoA dehydrogenase | Acadm | 46 | 0,0935 | 0,0165 | 17,7082 | 0,1123 | 0,0186 | 16,5361 |
| Decr1 protein | Decr1 | 36 | 0,1190 | 0,0198 | 16,6511 | 0,1349 | 0,0222 | 16,4621 |
| Single-stranded DNA-binding | Ssbp1 | 17 | 0,2469 | 0,0499 | 20,2143 | 0,3168 | 0,1017 | 32,0988 |
| Glycine amidinotransferase, mitochondrial | Gatm | 48 | 0,0965 | 0,0225 | 23,3656 | 0,0897 | 0,0309 | 34,4467 |
| Mitochondrial 2-oxoglutarate/malate carrier protein | Slc25a11 | 34 | 0,1254 | 0,0113 | 8,9953 | 0,1388 | 0,0235 | 16,8990 |
| Succinate--CoA ligase [GDP-forming] subunit beta | Suclg2 | 47 | 0,0982 | 0,0121 | 12,3026 | 0,0914 | 0,0078 | 8,5546 |
| Protein LYRIC | Mtdh | 64 | 0,0643 | 0,0076 | 11,7788 | 0,0763 | 0,0093 | 12,2584 |
| 17beta-hydroxysteroid dehydrogenase type 10/short chain L-3-hydroxyacyl-CoA dehydrogenase | Hsd17b10 | 27 | 0,1526 | 0,0312 | 20,4580 | 0,1797 | 0,0476 | 26,5156 |
| Ero1l protein | Ero1a | 54 | 0,0776 | 0,0051 | 6,5895 | 0,0882 | 0,0103 | 11,7272 |
| RNA helicase | Ddx17 | 73 | 0,0644 | 0,0029 | 4,4697 | 0,0534 | 0,0075 | 13,9681 |
| Cytoplasmic dynein 1 heavy chain | Dync1h1 | 532 | 0,0089 | 0,0023 | 25,3426 | 0,0067 | 0,0028 | 42,4106 |
| Large ribosomal subunit protein | Rpl28 | 16 | 0,2355 | 0,0321 | 13,6257 | 0,3464 | 0,0930 | 26,8588 |
| ATP-citrate synthase | Acly | 121 | 0,0353 | 0,0026 | 7,2809 | 0,0377 | 0,0100 | 26,4368 |
| Membrane-associated progesterone receptor | Pgrmc2 | 23 | 0,1487 | 0,0154 | 10,3669 | 0,2587 | 0,0441 | 17,0361 |
| Large ribosomal subunit protein | Rpl34 | 13 | 0,2890 | 0,0209 | 7,2278 | 0,4175 | 0,0201 | 4,8138 |
| Far upstream element-binding | Khsrp | 77 | 0,0769 | 0,0081 | 10,4782 | 0,0236 | 0,0134 | 56,7154 |
| Heterogeneous nuclear ribonucleoprotein K | Hnrnpk | 49 | 0,1296 | 0,0234 | 18,0502 | 0,0181 | 0,0154 | 84,7412 |
| Cluster of Small ribosomal subunit protein | Rps2 | 31 | 0,1290 | 0,0217 | 16,8521 | 0,1540 | 0,0325 | 21,0900 |
| Large ribosomal subunit protein | Rpl11 | 19 | 0,1935 | 0,0129 | 6,6523 | 0,2908 | 0,0574 | 19,7390 |
| RuvB-like helicase | Ruvbl2 | 51 | 0,0822 | 0,0066 | 8,0277 | 0,0874 | 0,0181 | 20,7710 |
| Ribosomal protein L19 | Rpl19 | 23 | 0,1745 | 0,0276 | 15,7960 | 0,2060 | 0,0231 | 11,2267 |
| Translocon-associated protein subunit | Ssr4 | 19 | 0,1889 | 0,0301 | 15,9164 | 0,2828 | 0,0246 | 8,7106 |
| B-cell receptor-associated | Bcap31 | 28 | 0,1282 | 0,0105 | 8,1728 | 0,2020 | 0,0571 | 28,2847 |
| 2-amino-3-ketobutyrate coenzyme A ligase | Gcat | 45 | 0,0968 | 0,0104 | 10,7083 | 0,0879 | 0,0236 | 26,8339 |
| Junction plakoglobin | Jup | 82 | 0,0402 | 0,0260 | 64,6884 | 0,0640 | 0,0472 | 73,7353 |
| ADP-dependent glucokinase | Adpgk | 54 | 0,0650 | 0,0078 | 12,0451 | 0,0983 | 0,0158 | 16,0669 |
| Apoptosis-inducing factor, mitochondrion | Aifm1 | 66 | 0,0625 | 0,0134 | 21,4767 | 0,0656 | 0,0164 | 24,9228 |
| Cluster of Small ribosomal subunit protein | Rps11 | 18 | 0,2188 | 0,0128 | 5,8480 | 0,2502 | 0,0643 | 25,7180 |
| Cytosol aminopeptidase | Lap3 | 56 | 0,0840 | 0,0087 | 10,3190 | 0,0575 | 0,0221 | 38,5049 |
| Neutral cholesterol ester hydrolase | Nceh1 | 46 | 0,0743 | 0,0210 | 28,2670 | 0,1216 | 0,0242 | 19,8849 |
| Phosphatidylinositol-3-phosphatase SAC1 | Sacm1l | 67 | 0,0633 | 0,0188 | 29,6646 | 0,0604 | 0,0156 | 25,8540 |
| Heterogeneous nuclear ribonucleoprotein F | Hnrnpf | 46 | 0,0924 | 0,0151 | 16,3569 | 0,0897 | 0,0238 | 26,5506 |
| Cytochrome c oxidase subunit | Cox4i1 | 20 | 0,1545 | 0,0496 | 32,1378 | 0,3040 | 0,0770 | 25,3466 |
| Very-long-chain 3-oxoacyl-CoA reductase | Hsd17b12 | 35 | 0,1050 | 0,0129 | 12,3075 | 0,1296 | 0,0278 | 21,4643 |
| Protein disulfide-isomerase TMX3 | Tmx3 | 52 | 0,0704 | 0,0120 | 17,0929 | 0,0910 | 0,0222 | 24,3581 |
| ATP synthase subunit g | Atp5mg | 11 | 0,3498 | 0,0251 | 7,1746 | 0,3926 | 0,0399 | 10,1707 |
| Thioredoxin domain-containing protein | Erp44 | 47 | 0,1216 | 0,0176 | 14,4651 | 0,0260 | 0,0061 | 23,5822 |
| Small ribosomal subunit protein u | Rps18-ps5 | 18 | 0,1807 | 0,0119 | 6,5778 | 0,2924 | 0,0296 | 10,1322 |
| Gag protein | gag | 65 | 0,0528 | 0,0058 | 10,9545 | 0,0760 | 0,0159 | 20,9285 |
| Heterogeneous nuclear ribonucleoproteins A2 | Hnrnpa2b1 | 37 | 0,1615 | 0,0084 | 5,2034 | 0,0175 | 0,0180 | 102,8556 |
| Transmembrane emp24 domain-containing | Tmed4 | 26 | 0,1243 | 0,0298 | 23,9912 | 0,1928 | 0,0498 | 25,8144 |
| PKD domain-containing protein | Gpnmb | 64 | 0,0629 | 0,0027 | 4,2596 | 0,0573 | 0,0059 | 10,2688 |
| Small ribosomal subunit protein | Rps4x | 30 | 0,1273 | 0,0296 | 23,2397 | 0,1284 | 0,0177 | 13,7667 |
| Cluster of Eukaryotic translation elongation factor 1 delta (guanine nucleotide exchange protein | Eef1d | 73 | 0,0553 | 0,0090 | 16,2275 | 0,0492 | 0,0132 | 26,7951 |
| Nicalin | Ncln | 63 | 0,0543 | 0,0096 | 17,6837 | 0,0739 | 0,0216 | 29,2414 |
| Ras-related protein Rab | Rab18 | 23 | 0,1486 | 0,0078 | 5,2280 | 0,1998 | 0,0534 | 26,7371 |
| Uncharacterized protein (Fragment | Pmpcb | 54 | 0,0682 | 0,0074 | 10,7870 | 0,0750 | 0,0049 | 6,5487 |
| Endoplasmic reticulum-Golgi intermediate compartment | Ergic1 | 33 | 0,1086 | 0,0157 | 14,4395 | 0,1301 | 0,0111 | 8,5546 |
| Large ribosomal subunit protein | Rpl35a | 13 | 0,2440 | 0,0730 | 29,9029 | 0,3866 | 0,0699 | 18,0904 |
| Microtubule-associated protein 4 | Map4 | 118 | 0,0490 | 0,0019 | 3,9005 | 0,0052 | 0,0012 | 23,5822 |
| Cluster of AP complex subunit beta | Ap2b1 | 107 | 0,0331 | 0,0026 | 7,9004 | 0,0381 | 0,0111 | 29,1982 |
| CD44 antigen | Cd44 | 72 | 0,0449 | 0,0059 | 13,2372 | 0,0622 | 0,0083 | 13,3316 |
| Signal recognition particle receptor subunit | Srpra | 70 | 0,0538 | 0,0046 | 8,4928 | 0,0505 | 0,0120 | 23,8371 |
| TAP binding protein | Tapbp | 50 | 0,0650 | 0,0130 | 19,9808 | 0,0891 | 0,0111 | 12,4779 |
| DnaJ homolog subfamily | Dnaja2 | 46 | 0,0759 | 0,0101 | 13,3043 | 0,0841 | 0,0148 | 17,5451 |
| Mitochondrial carrier homolog 2 | Mtch2 | 33 | 0,1010 | 0,0075 | 7,4193 | 0,1300 | 0,0208 | 15,9716 |
| Golgi-resident adenosine 3',5'-bisphosphate | Bpnt2 | 39 | 0,0902 | 0,0138 | 15,2853 | 0,1032 | 0,0130 | 12,5888 |
| OCIA domain-containing protein | Ociad1 | 28 | 0,1198 | 0,0200 | 16,6829 | 0,1522 | 0,0306 | 20,1242 |
| Sphingosine-1-phosphate lyase | Sgpl1 | 64 | 0,0586 | 0,0063 | 10,6893 | 0,0534 | 0,0152 | 28,5100 |
| Enoyl reductase (ER) domain-containing | Vat1 | 43 | 0,0857 | 0,0090 | 10,5551 | 0,0824 | 0,0183 | 22,2599 |
| Dolichyl-diphosphooligosaccharide--protein glycosyltransferase subunit | Stt3a | 81 | 0,0419 | 0,0137 | 32,7935 | 0,0488 | 0,0131 | 26,8339 |
| Peroxiredoxin-1 | Prdx1 | 22 | 0,1816 | 0,0346 | 19,0242 | 0,1412 | 0,0146 | 10,3224 |
| PAT complex subunit CCDC47 | Ccdc47 | 56 | 0,0780 | 0,0068 | 8,6800 | 0,0413 | 0,0191 | 46,2216 |
| Suppression of tumorigenicity 13 | St13 | 41 | 0,1146 | 0,0138 | 12,0492 | 0,0462 | 0,0121 | 26,2951 |
| Cytochrome c, somatic | Cycs | 12 | 0,4350 | 0,0091 | 2,1028 | 0,0794 | 0,0700 | 88,1517 |
| Cluster of Histone H2A | H2aj | 14 | 0,2204 | 0,0241 | 10,9520 | 0,3325 | 0,0972 | 29,2414 |
| Enoyl-CoA delta isomerase | Eci1 | 32 | 0,1093 | 0,0083 | 7,6026 | 0,1159 | 0,0174 | 14,9862 |
| Succinate-CoA ligase subunit beta | Sucla2 | 51 | 0,0636 | 0,0108 | 16,9309 | 0,0866 | 0,0281 | 32,5054 |
| Cluster of Y-box-binding | Ybx1 | 36 | 0,1448 | 0,0245 | 16,9356 | 0,0250 | 0,0185 | 73,8033 |
| Short-chain specific acyl-CoA dehydrogenase | Acads | 45 | 0,0663 | 0,0091 | 13,7629 | 0,1004 | 0,0086 | 8,6053 |
| SPARC | Sparc | 38 | 0,0903 | 0,0099 | 10,9545 | 0,1025 | 0,0229 | 22,3379 |
| Large ribosomal subunit protein | Rpl23 | 15 | 0,2229 | 0,0262 | 11,7671 | 0,2662 | 0,0365 | 13,7199 |
| Eukaryotic initiation factor 4A | Eif4a1 | 46 | 0,0894 | 0,0119 | 13,2731 | 0,0569 | 0,0208 | 36,6349 |
| NADH-ubiquinone oxidoreductase 75 kDa subunit | Ndufs1 | 80 | 0,0481 | 0,0053 | 11,0267 | 0,0364 | 0,0195 | 53,6436 |
| NAD kinase 2, mitochondrial | Nadk2 | 51 | 0,0722 | 0,0170 | 23,5511 | 0,0638 | 0,0114 | 17,9199 |
| Golgi apparatus protein 1 | Glg1 | 132 | 0,0266 | 0,0051 | 19,3305 | 0,0255 | 0,0071 | 27,7052 |
| SEC63 | Sec63 | 88 | 0,0438 | 0,0027 | 6,2551 | 0,0321 | 0,0059 | 18,3257 |
| Poly(rC)-binding protein | Pcbp1 | 37 | 0,1208 | 0,0264 | 21,8747 | 0,0473 | 0,0235 | 49,5472 |
| Basigin | Bsg | 24 | 0,1210 | 0,0160 | 13,2031 | 0,1822 | 0,0274 | 15,0200 |
| Reticulon-3 | Rtn3 | 104 | 0,0296 | 0,0015 | 5,1580 | 0,0401 | 0,0055 | 13,6986 |
| Vesicular integral-membrane protein | Lman2 | 40 | 0,0871 | 0,0178 | 20,4380 | 0,0859 | 0,0303 | 35,3319 |
| Complement component 1 Q subcomponent-binding protein | C1qbp | 31 | 0,1408 | 0,0235 | 16,6555 | 0,0616 | 0,0176 | 28,5899 |
| Polyadenylate-binding protein | Pabpc4 | 69 | 0,0695 | 0,0073 | 10,4358 | 0,0172 | 0,0063 | 36,3893 |
| RuvB-like 1 | Ruvbl1 | 50 | 0,0734 | 0,0031 | 4,2550 | 0,0584 | 0,0195 | 33,4306 |
| Small ribosomal subunit protein | Rps8 | 24 | 0,1321 | 0,0199 | 15,0474 | 0,1610 | 0,0277 | 17,2095 |
| GOLD domain-containing protein | Tmed9 | 31 | 0,0914 | 0,0185 | 20,2932 | 0,1471 | 0,0401 | 27,2277 |
| Procollagen galactosyltransferase 1 | Colgalt1 | 71 | 0,0471 | 0,0062 | 13,1367 | 0,0513 | 0,0128 | 24,9655 |
| Signal recognition particle receptor subunit | Srprb | 30 | 0,1056 | 0,0073 | 6,9332 | 0,1210 | 0,0122 | 10,0945 |
| Small ribosomal subunit protein | Rps15a | 15 | 0,2057 | 0,0225 | 10,9520 | 0,2544 | 0,0609 | 23,9234 |
| Tripartite motif-containing 28 | Trim28 | 89 | 0,0402 | 0,0072 | 18,0355 | 0,0318 | 0,0104 | 32,7827 |
| Cluster of small monomeric GTPase | Rap1b | 21 | 0,1508 | 0,0154 | 10,2082 | 0,1755 | 0,0115 | 6,5619 |
| P-type Cu(+) transporter | Atp7a | 162 | 0,0205 | 0,0038 | 18,4607 | 0,0196 | 0,0076 | 38,5428 |
| Casein kinase II subunit | Csnk2a1 | 45 | 0,0725 | 0,0174 | 23,9833 | 0,0788 | 0,0265 | 33,6525 |
| Bifunctional methylenetetrahydrofolate dehydrogenase/cyclohydrolase | Mthfd2 | 38 | 0,0994 | 0,0112 | 11,3014 | 0,0655 | 0,0215 | 32,7663 |
| Serine/arginine repetitive matrix | Srrm2 | 296 | 0,0086 | 0,0046 | 53,6889 | 0,0159 | 0,0082 | 51,8702 |
| Transport and Golgi organization protein | Mia3 | 214 | 0,0156 | 0,0033 | 21,3573 | 0,0145 | 0,0080 | 55,6235 |
| Serine/arginine-rich splicing | Srsf7 | 27 | 0,1876 | 0,0257 | 13,6987 | 0,0167 | 0,0123 | 73,8033 |
| Thioredoxin-related transmembrane protein | Tmx1 | 31 | 0,0885 | 0,0288 | 32,4893 | 0,1446 | 0,0484 | 33,4527 |
| Ras GTPase-activating protein-binding | G3bp1 | 52 | 0,0621 | 0,0135 | 21,8199 | 0,0642 | 0,0167 | 26,0861 |
| Eukaryotic translation initiation factor 3 subunit | Eif3a | 162 | 0,0211 | 0,0058 | 27,4204 | 0,0176 | 0,0073 | 41,5356 |
| ATP synthase subunit d, mitochondrial (Fragment | Atp5pd | 16 | 0,2141 | 0,0318 | 14,8714 | 0,1832 | 0,0272 | 14,8429 |
| Mitochondrial-processing peptidase subunit | Pmpca | 58 | 0,0518 | 0,0051 | 9,9305 | 0,0621 | 0,0124 | 19,8798 |
| Prenylcysteine lyase domain-containing | Pcyox1 | 56 | 0,0564 | 0,0095 | 16,8556 | 0,0607 | 0,0124 | 20,4999 |
| Cluster of Gag polyprotein | Q1KYL8 | 61 | 0,0465 | 0,0172 | 37,1160 | 0,0658 | 0,0199 | 30,2164 |
| Interferon-induced transmembrane protein | Ifitm3 | 15 | 0,1548 | 0,0281 | 18,1418 | 0,3323 | 0,0661 | 19,8913 |
| Cluster of 130kDa Protein 4.1B MEF cell isoform | Epb41l3 | 98 | 0,0418 | 0,0029 | 6,8267 | 0,0173 | 0,0040 | 23,1452 |
| Coatomer subunit delta | Arcn1 | 57 | 0,0844 | 0,0130 | 15,3380 | 0,0083 | 0,0111 | 132,6393 |
| Heterogeneous nuclear ribonucleoprotein A | Hnrnpab |  | 0,1431 | 0,0194 | 13,5463 | 0,0000 | 0,0000 |  |
| H3 clustered histone 14 | H3c14 | 20 | 0,1373 | 0,0177 | 12,9216 | 0,2036 | 0,0358 | 17,5658 |
| CDGSH iron-sulfur domain-containing | Cisd1 | 12 | 0,2351 | 0,0053 | 2,2745 | 0,3307 | 0,0897 | 27,1355 |
| Small ribosomal subunit protein | Mrps34 | 26 | 0,1221 | 0,0164 | 13,4524 | 0,1213 | 0,0268 | 22,1251 |
| Prenylcysteine oxidase-like | Pcyox1l | 55 | 0,0560 | 0,0048 | 8,6499 | 0,0586 | 0,0161 | 27,5326 |
| Coatomer subunit beta' | Copb2 | 102 | 0,0326 | 0,0040 | 12,3164 | 0,0273 | 0,0088 | 32,1053 |
| Coatomer subunit beta | Copb1 | 108 | 0,0318 | 0,0045 | 14,0221 | 0,0252 | 0,0091 | 36,2466 |
| Thiosulfate sulfurtransferase | Tst | 33 | 0,0964 | 0,0163 | 16,9165 | 0,0973 | 0,0248 | 25,4742 |
| Atlastin GTPase 3 | Atl3 | 60 | 0,0581 | 0,0119 | 20,4380 | 0,0423 | 0,0127 | 29,9360 |
| Stomatin-like protein 2 | Stoml2 | 38 | 0,0881 | 0,0083 | 9,3815 | 0,0719 | 0,0235 | 32,7141 |
| Complex I assembly factor | Acad9 | 69 | 0,0471 | 0,0027 | 5,7818 | 0,0410 | 0,0075 | 18,3257 |
| 26S proteasome regulatory subunit | Psmc2 | 49 | 0,0715 | 0,0057 | 7,9004 | 0,0502 | 0,0145 | 28,9809 |
| Uncharacterized protein | Tomm70a | 68 | 0,0476 | 0,0073 | 15,4000 | 0,0430 | 0,0093 | 21,6256 |
| Succinate dehydrogenase [ubiquinone] iron-sulfur subunit, mitochondrial | Sdhb | 27 | 0,1264 | 0,0272 | 21,5150 | 0,0954 | 0,0137 | 14,3375 |
| K Homology domain-containing | Hdlbp | 142 | 0,0211 | 0,0028 | 13,3779 | 0,0215 | 0,0093 | 43,1896 |
| FAS-associated factor 2 | Faf2 | 52 | 0,0589 | 0,0101 | 17,1468 | 0,0564 | 0,0176 | 31,2301 |
| Isoleucine--tRNA ligase, mitochondrial | Iars2 | 113 | 0,0242 | 0,0054 | 22,2473 | 0,0321 | 0,0032 | 10,0945 |
| Vesicle-fusing ATPase | Nsf | 83 | 0,0390 | 0,0052 | 13,2372 | 0,0323 | 0,0161 | 49,8542 |
| Poly(RC) binding protein | Pcbp2 | 38 | 0,0835 | 0,0075 | 9,0124 | 0,0717 | 0,0306 | 42,6834 |
| Cluster of Myosin-9 | Myh9 | 227 | 0,0166 | 0,0053 | 32,2111 | 0,0079 | 0,0059 | 73,8080 |
| Cluster of Ras-related protein | Rras2 | 23 | 0,1228 | 0,0187 | 15,1925 | 0,1505 | 0,0584 | 38,7852 |
| PexRAP | Dhrs7b | 35 | 0,0829 | 0,0228 | 27,4500 | 0,0899 | 0,0189 | 21,0592 |
| Sideroflexin-1 | Sfxn1 | 36 | 0,0710 | 0,0149 | 20,9536 | 0,1054 | 0,0072 | 6,8019 |
| Myoferlin | Myof | 236 | 0,0124 | 0,0005 | 4,0193 | 0,0136 | 0,0012 | 8,9236 |
| Small ribosomal subunit protein | Rps19 | 16 | 0,1875 | 0,0238 | 12,7052 | 0,1742 | 0,1053 | 60,4311 |
| Splicing factor 3A subunit | Sf3a1 | 89 | 0,0346 | 0,0035 | 9,9665 | 0,0306 | 0,0131 | 42,6834 |
| Cluster of Tropomyosin | Tpm1 | 32 | 0,1441 | 0,0145 | 10,0647 | 0,0096 | 0,0120 | 124,2177 |
| Cluster of Fibrillin-2 | Fbn2 | 314 | 0,0150 | 0,0030 | 19,7650 | 0,0006 | 0,0013 | 200,0000 |
| 40S ribosomal protein SA | Rpsa | 33 | 0,0781 | 0,0130 | 16,6998 | 0,1143 | 0,0214 | 18,7224 |
| Valine--tRNA ligase | Vars1 | 140 | 0,0177 | 0,0024 | 13,3370 | 0,0266 | 0,0049 | 18,2399 |
| RAS-related C3 botulinum | Rac1 | 21 | 0,1348 | 0,0130 | 9,6226 | 0,1514 | 0,0294 | 19,4319 |
| Mitochondrial adenyl nucleotide antiporter | Slc25a24 | 53 | 0,0533 | 0,0182 | 34,0796 | 0,0603 | 0,0170 | 28,1282 |
| Sideroflexin-3 | Sfxn3 | 35 | 0,0783 | 0,0037 | 4,6620 | 0,0928 | 0,0236 | 25,4830 |
| Cluster of Synaptotagmin binding, cytoplasmic RNA interacting | Syncrip | 63 | 0,0638 | 0,0068 | 10,6720 | 0,0177 | 0,0079 | 44,3368 |
| GTP-binding nuclear protein | Ran | 24 | 0,1141 | 0,0110 | 9,6708 | 0,1318 | 0,0277 | 21,0311 |
| Sodium/potassium-transporting ATPase subunit beta | Atp1b3 | 32 | 0,0720 | 0,0199 | 27,6951 | 0,1307 | 0,0495 | 37,9103 |
| Anoctamin-6 | Ano6 | 107 | 0,0256 | 0,0056 | 21,8188 | 0,0302 | 0,0063 | 21,0137 |
| Cluster of Acyl-Coenzyme A dehydrogenase, short/branched | Acadsb | 48 | 0,0573 | 0,0082 | 14,3973 | 0,0639 | 0,0226 | 35,3837 |
| Mitochondrial import receptor subunit | Tomm40 | 38 | 0,0764 | 0,0099 | 12,9374 | 0,0736 | 0,0211 | 28,7038 |
| ATP synthase subunit e | Atp5me | 8 | 0,2162 | 0,1119 | 51,7635 | 0,6337 | 0,1194 | 18,8387 |
| RNA helicase | Dhx9 | 150 | 0,0222 | 0,0042 | 18,9070 | 0,0141 | 0,0053 | 37,7139 |
| Cluster of Ras-related protein | Rala | 24 | 0,0960 | 0,0300 | 31,2069 | 0,1638 | 0,0329 | 20,0956 |
| Enoyl-CoA hydratase, mitochondrial | Echs1 | 31 | 0,0939 | 0,0067 | 7,1186 | 0,0932 | 0,0241 | 25,8890 |
| Leucyl-cystinyl aminopeptidase | Lnpep | 118 | 0,0261 | 0,0076 | 29,2383 | 0,0213 | 0,0023 | 10,9622 |
| Transmembrane 9 superfamily member | Tm9sf2 | 75 | 0,0350 | 0,0089 | 25,2792 | 0,0422 | 0,0089 | 21,0311 |
| H-2D cell surface | H2-D1 | 38 | 0,0743 | 0,0084 | 11,3399 | 0,0717 | 0,0306 | 42,6834 |
| Thioredoxin-dependent peroxide reductase | Prdx3 | 28 | 0,1625 | 0,0284 | 17,5051 | 0,0000 | 0,0000 |  |
| Ribosomal protein S14 | rps14 | 16 | 0,1495 | 0,0289 | 19,3176 | 0,2203 | 0,0197 | 8,9586 |
| Importin subunit beta-1 | Kpnb1 | 97 | 0,0257 | 0,0050 | 19,5662 | 0,0347 | 0,0105 | 30,1471 |
| Large ribosomal subunit protein | Rpl37a | 10 | 0,2556 | 0,0337 | 13,1966 | 0,3316 | 0,1723 | 51,9619 |
| Cluster | H1-4 | 22 | 0,1276 | 0,0277 | 21,7069 | 0,1299 | 0,0337 | 25,8974 |
| V-type proton ATPase catalytic subunit A OS=Mus | Atp6v1a | 68 | 0,0392 | 0,0064 | 16,3349 | 0,0427 | 0,0196 | 45,7565 |
| Ceramide synthase 2 | Cers2 | 45 | 0,0609 | 0,0116 | 19,0395 | 0,0660 | 0,0358 | 54,1632 |
| Krt78 protein (Fragment | Krt78 | 55 | 0,0358 | 0,0117 | 32,6496 | 0,0747 | 0,0233 | 31,2518 |
| Guanine nucleotide-binding protein subunit | Gna13 | 44 | 0,0621 | 0,0043 | 6,9951 | 0,0663 | 0,0063 | 9,4821 |
| Heterogeneous nuclear ribonucleoprotein A1 | Hnrnpa1 | 34 | 0,1081 | 0,0065 | 5,9931 | 0,0380 | 0,0138 | 36,3730 |
| Small ribosomal subunit protein | Rps6 | 29 | 0,0882 | 0,0076 | 8,6424 | 0,1073 | 0,0206 | 19,2239 |
| Methylmalonyl-CoA mutase, mitochondrial | Mmut | 83 | 0,0318 | 0,0060 | 18,8135 | 0,0350 | 0,0058 | 16,5862 |
| Cytochrome b-c1 complex | Uqcrq | 10 | 0,2564 | 0,0121 | 4,7343 | 0,3095 | 0,0861 | 27,8115 |
| Cluster of Fibroblast growth factor | Fgfr3 | 88 | 0,0320 | 0,0076 | 23,6617 | 0,0311 | 0,0135 | 43,3639 |
| Gamma-aminobutyraldehyde dehydrogenase | Aldh9a1 | 54 | 0,0570 | 0,0112 | 19,7046 | 0,0362 | 0,0271 | 74,8500 |
| 5'-nucleotidase domain-containing protein | Nt5dc3 | 63 | 0,0474 | 0,0094 | 19,9025 | 0,0344 | 0,0167 | 48,5259 |
| Cluster of Transgelin-2 | Tagln2 | 22 | 0,1794 | 0,0218 | 12,1398 | 0,0328 | 0,0065 | 19,8798 |
| Cluster of RNA-binding | Fus | 53 | 0,0820 | 0,0098 | 11,9842 | 0,0000 | 0,0000 |  |
| Putative lipid scramblase CLPTM1 | Clptm1 | 75 | 0,0343 | 0,0063 | 18,4716 | 0,0395 | 0,0034 | 8,5424 |
| Cluster of Ribose-phosphate | Prps2 | 35 | 0,0855 | 0,0138 | 16,1604 | 0,0636 | 0,0139 | 21,9165 |
| DnaJ homolog subfamily | Dnaja1 | 45 | 0,0644 | 0,0113 | 17,5531 | 0,0515 | 0,0147 | 28,6258 |
| 3-ketoacyl-CoA thiolase A, peroxisomal | Acaa1a | 44 | 0,0663 | 0,0206 | 30,9908 | 0,0527 | 0,0200 | 37,8903 |
| 26S proteasome non-ATPase regulatory | Psmd1 | 107 | 0,0234 | 0,0040 | 16,9335 | 0,0282 | 0,0047 | 16,8116 |
| Adenylate kinase 2, mitochondrial | Ak2 | 26 | 0,1651 | 0,0272 | 16,4486 | 0,0000 | 0,0000 |  |
| Signal peptidase complex subunit | Spcs2 | 25 | 0,0923 | 0,0116 | 12,6124 | 0,1294 | 0,0167 | 12,8847 |
| Small ribosomal subunit protein | Rps20 | 13 | 0,1971 | 0,0445 | 22,5851 | 0,2152 | 0,0459 | 21,3129 |
| Cluster of Fumarate hydratase | Fh | 54 | 0,0475 | 0,0033 | 7,0056 | 0,0493 | 0,0150 | 30,3531 |
| Peroxisomal acyl-coenzyme | Acox1 | 75 | 0,0443 | 0,0111 | 25,1335 | 0,0163 | 0,0179 | 109,7246 |
| Cation-dependent mannose-6-phosphate | M6pr | 31 | 0,0741 | 0,0127 | 17,1468 | 0,1030 | 0,0233 | 22,6567 |
| Vesicle-associated membrane protein, associated protein | Vapb | 27 | 0,0960 | 0,0338 | 35,2475 | 0,1247 | 0,0217 | 17,4361 |
| Serine/arginine-rich splicing | Srsf3 | 19 | 0,1528 | 0,0198 | 12,9374 | 0,1125 | 0,0360 | 31,9503 |
| NADH-cytochrome b5 reductase | Cyb5r1 | 34 | 0,0728 | 0,0077 | 10,6381 | 0,0815 | 0,0335 | 41,0887 |
| Cluster of Kinectin 1 | Ktn1 | 143 | 0,0196 | 0,0072 | 36,9169 | 0,0137 | 0,0100 | 73,2048 |
| Ribosomal protein S5 (Fragment | Rps5 | 20 | 0,1111 | 0,0219 | 19,6945 | 0,1494 | 0,0251 | 16,8116 |
| Dephospho-CoA kinase domain-containing | Dcakd | 26 | 0,0784 | 0,0180 | 22,9666 | 0,1280 | 0,0072 | 5,6412 |
| Enoyl-[acyl-carrier-protein] reductase | Mecr | 40 | 0,0621 | 0,0091 | 14,5922 | 0,0660 | 0,0221 | 33,4900 |
| Acyl-Coenzyme A dehydrogenase, very long | Acadvl | 69 | 0,0346 | 0,0061 | 17,5213 | 0,0398 | 0,0067 | 16,8854 |
| 26S proteasome regulatory subunit | Psmc6 | 44 | 0,0586 | 0,0115 | 19,5935 | 0,0552 | 0,0202 | 36,5927 |
| Glutamyl-prolyl-tRNA synthetase | Eprs1 | 170 | 0,0151 | 0,0023 | 15,4967 | 0,0137 | 0,0051 | 36,9370 |
| ENH1 | Pdlim5 | 63 | 0,0475 | 0,0024 | 5,0717 | 0,0257 | 0,0180 | 69,9930 |
| Large ribosomal subunit protein | Rpl27a | 17 | 0,1253 | 0,0313 | 24,9898 | 0,1809 | 0,0090 | 4,9677 |
| Cytochrome P450 CYP2J9 | Cyp2j9 | 58 | 0,0412 | 0,0076 | 18,4195 | 0,0461 | 0,0125 | 27,1031 |
| Very-long-chain enoyl-Co | Tecr | 36 | 0,0589 | 0,0149 | 25,3411 | 0,0801 | 0,0293 | 36,5147 |
| Erlin-1 | Erlin1 | 39 | 0,0590 | 0,0055 | 9,3879 | 0,0693 | 0,0176 | 25,4162 |
| Cytochrome b-c1 complex subunit Rieske | Uqcrfs1 | 29 | 0,0858 | 0,0089 | 10,4117 | 0,0827 | 0,0279 | 33,7221 |
| Rho guanine nucleotide exchange | Arhgef2 | 110 | 0,0249 | 0,0035 | 13,9331 | 0,0169 | 0,0118 | 70,1420 |
| Protein canopy homolog 2 | Cnpy2 | 21 | 0,1719 | 0,0556 | 32,3508 | 0,0299 | 0,0257 | 86,0848 |
| Coronin | Coro1c | 53 | 0,0645 | 0,0133 | 20,5793 | 0,0135 | 0,0197 | 146,5962 |
| Nucleolar and coiled-body | Nolc1 | 73 | 0,0506 | 0,0143 | 28,3724 | 0,0054 | 0,0074 | 136,0264 |
| Cluster of Tpm3 protein | Tpm3 | 29 | 0,1294 | 0,0182 | 14,0874 | 0,0038 | 0,0076 | 200,0000 |
| Mitochondrial import inner membrane translocase | Timm50 | 40 | 0,0577 | 0,0037 | 6,3381 | 0,0673 | 0,0101 | 14,9409 |
| Protein-serine/threonine kinase | Pdk3 | 48 | 0,0444 | 0,0054 | 12,2161 | 0,0591 | 0,0194 | 32,7827 |
| Eukaryotic translation initiation factor 3 subunit | Eif3i | 36 | 0,0615 | 0,0095 | 15,4762 | 0,0737 | 0,0267 | 36,1729 |
| Methylmalonate-semialdehyde/malonate-semialdehyde dehydrogenase [acylating], mitochondrial | Aldh6a1 | 58 | 0,0414 | 0,0056 | 13,4255 | 0,0427 | 0,0113 | 26,4578 |
| Large ribosomal subunit protein | Rpl31 | 14 | 0,1768 | 0,0179 | 10,1411 | 0,1632 | 0,0551 | 33,7923 |
| Hydroxyacyl-coenzyme A dehydrogenase | Hadh | 34 | 0,0727 | 0,0129 | 17,7259 | 0,0659 | 0,0296 | 44,8858 |
| LIM zinc-binding domain-containing | Lima1 | 66 | 0,0530 | 0,0098 | 18,4856 | 0,0085 | 0,0060 | 70,7018 |
| Endoplasmic reticulum resident protein | Erp29 | 29 | 0,1319 | 0,0255 | 19,2996 | 0,0000 | 0,0000 |  |
| Dehydrogenase/reductase (SDR family) member | Dhrs7 | 41 | 0,0541 | 0,0096 | 17,8364 | 0,0645 | 0,0129 | 19,9853 |
| Transmembrane protein 43 | Tmem43 | 45 | 0,0475 | 0,0070 | 14,8008 | 0,0632 | 0,0186 | 29,5032 |
| Malectin | Mlec | 32 | 0,0642 | 0,0086 | 13,3686 | 0,0931 | 0,0244 | 26,1679 |
| Large ribosomal subunit protein | Rpl32 | 16 | 0,1281 | 0,0290 | 22,6786 | 0,1795 | 0,0618 | 34,4467 |
| Large ribosomal subunit protein | Rpl22 | 15 | 0,1938 | 0,0078 | 4,0193 | 0,0982 | 0,0137 | 13,9667 |
| Tyrosine-protein phosphatase non-receptor | Ptpn1 | 50 | 0,0445 | 0,0088 | 19,6945 | 0,0515 | 0,0074 | 14,3375 |
| Eif2s2 protein | Eif2s2 | 38 | 0,0721 | 0,0034 | 4,6620 | 0,0432 | 0,0294 | 67,9673 |
| AAA+ ATPase domain-containing | Psmc5 | 47 | 0,0532 | 0,0179 | 33,6127 | 0,0439 | 0,0259 | 58,9491 |
| J domain-containing protein | Dnajc3 | 57 | 0,0465 | 0,0075 | 16,0581 | 0,0326 | 0,0223 | 68,2450 |
| RNA-binding protein 3 | Rbm3 | 17 | 0,2106 | 0,0308 | 14,6044 | 0,0181 | 0,0225 | 124,2177 |
| Isovaleryl-CoA dehydrogenase, mitochondrial | Ivd | 46 | 0,0409 | 0,0146 | 35,5679 | 0,0687 | 0,0123 | 17,8988 |
| Small ribosomal subunit protein | Rps13 | 17 | 0,1204 | 0,0114 | 9,4812 | 0,1650 | 0,0156 | 9,4386 |
| Transmembrane 9 superfamily member | Tm9sf3 | 72 | 0,0237 | 0,0051 | 21,5150 | 0,0467 | 0,0059 | 12,5975 |
| Cluster of Polypyrimidine tract-binding | Ptbp1 | 56 | 0,0445 | 0,0072 | 16,2184 | 0,0354 | 0,0125 | 35,3972 |
| Large ribosomal subunit protein | Rpl23a | 18 | 0,1480 | 0,0235 | 15,9014 | 0,0981 | 0,0455 | 46,3847 |
| Erlin-2 | Erlin2 | 38 | 0,0587 | 0,0199 | 33,8545 | 0,0649 | 0,0045 | 6,9797 |
| Sulfatase-modifying factor enzyme domain-containing protein | Sumf2 | 34 | 0,0777 | 0,0126 | 16,2257 | 0,0464 | 0,0305 | 65,8077 |
| Protein disulfide isomerase Creld2 | Creld2 | 38 | 0,0970 | 0,0131 | 13,4696 | 0,0000 | 0,0000 |  |
| Secretory carrier-associated membrane | Gm45927 | 89 | 0,0222 | 0,0090 | 40,6102 | 0,0317 | 0,0049 | 15,3702 |
| Integrin alpha-2 | Itga2 | 130 | 0,0172 | 0,0031 | 17,8364 | 0,0176 | 0,0038 | 21,4643 |
| Acyl-Coenzyme A dehydrogenase family | Acad8 | 45 | 0,0476 | 0,0051 | 10,7508 | 0,0546 | 0,0158 | 28,9809 |
| Pyrroline-5-carboxylate reductase | Pycr1 | 32 | 0,0615 | 0,0289 | 46,9537 | 0,0839 | 0,0183 | 21,7496 |
| Eukaryotic translation initiation factor 3 subunit | Eif3c | 107 | 0,0218 | 0,0039 | 18,0777 | 0,0191 | 0,0097 | 50,7334 |
| RNA helicase | Eif4a3l2 | 47 | 0,0455 | 0,0067 | 14,8008 | 0,0501 | 0,0237 | 47,2968 |
| 3-hydroxyisobutyrate dehydrogenase, mitochondrial | Hibadh | 35 | 0,0977 | 0,0101 | 10,3669 | 0,0086 | 0,0100 | 115,8293 |
| Vesicle-associated membrane protein | Vamp3 | 11 | 0,1403 | 0,0421 | 30,0122 | 0,3261 | 0,1265 | 38,7788 |
| Cluster of Ig-like domain-containing | Pvr | 45 | 0,0402 | 0,0090 | 22,2690 | 0,0698 | 0,0354 | 50,6709 |
| Large ribosomal subunit protein | Rpl35 | 15 | 0,1314 | 0,0132 | 10,0832 | 0,1738 | 0,0135 | 7,7911 |
| Cytochrome c oxidase subunit | Cox6c | 8 | 0,2038 | 0,0440 | 21,5703 | 0,4129 | 0,0666 | 16,1272 |
| Magnesium transporter 1 | Magt1 | 42 | 0,0532 | 0,0069 | 12,9440 | 0,0518 | 0,0157 | 30,2916 |
| Cluster of Transforming protein | Rhoa | 22 | 0,0851 | 0,0170 | 20,0066 | 0,1275 | 0,0120 | 9,4386 |
| Transmembrane 9 superfamily member | Tm9sf4 | 75 | 0,0295 | 0,0116 | 39,4205 | 0,0297 | 0,0065 | 21,9165 |
| Membrane-associated progesterone receptor | Pgrmc1 | 22 | 0,0661 | 0,0147 | 22,2883 | 0,1593 | 0,0318 | 19,9869 |
| Histone H1.1 OS | H1-1 | 22 | 0,1046 | 0,0274 | 26,2438 | 0,0933 | 0,0134 | 14,3809 |
| Small ribosomal subunit protein | Rps23 | 16 | 0,1234 | 0,0234 | 18,9525 | 0,1615 | 0,0639 | 39,5436 |
| Peptidyl-tRNA hydrolase 2 | Ptrh2 | 20 | 0,0985 | 0,0267 | 27,0652 | 0,1266 | 0,0635 | 50,1389 |
| E3 UFM1-protein ligase | Ufl1 | 90 | 0,0248 | 0,0047 | 18,7423 | 0,0229 | 0,0159 | 69,4697 |
| Dihydrolipoyllysine-residue succinyltransferase component of 2-oxoglutarate dehydrogenase complex | Dlst | 49 | 0,0633 | 0,0136 | 21,5600 | 0,0151 | 0,0109 | 72,5047 |
| Chloride channel CLIC-like | Clcc1 | 61 | 0,0253 | 0,0039 | 15,4619 | 0,0545 | 0,0168 | 30,7441 |
| Large ribosomal subunit protein | Mrpl15 | 34 | 0,0577 | 0,0165 | 28,5161 | 0,0685 | 0,0253 | 36,9370 |
| V-type proton ATPase subunit a OS=Mus | Atp6v0a1 | 96 | 0,0240 | 0,0027 | 11,3375 | 0,0195 | 0,0071 | 36,6996 |
| DnaJ homolog subfamily | Dnajc10 | 91 | 0,0262 | 0,0078 | 29,8680 | 0,0182 | 0,0068 | 37,1190 |
| Eukaryotic translation initiation factor 5A (Fragment | Eif5a | 16 | 0,1974 | 0,0289 | 14,6237 | 0,0282 | 0,0208 | 73,8033 |
| Cell adhesion molecule 4 | Cadm4 | 43 | 0,0357 | 0,0074 | 20,6796 | 0,0698 | 0,0170 | 24,3076 |
| ATP-dependent RNA helicase | Ddx1 | 83 | 0,0236 | 0,0067 | 28,5161 | 0,0282 | 0,0047 | 16,7532 |
| ATP synthase subunit f | Atp5mf | 9 | 0,2102 | 0,0453 | 21,5406 | 0,2666 | 0,0899 | 33,7221 |
| Mitochondrial import inner membrane translocase | Timm44 | 51 | 0,0367 | 0,0073 | 20,0066 | 0,0474 | 0,0098 | 20,6271 |
| Large ribosomal subunit protein | Rpl27 | 16 | 0,1162 | 0,0441 | 37,9161 | 0,1571 | 0,0284 | 18,0904 |
| Small ribosomal subunit protein | Rps17 | 16 | 0,1382 | 0,0330 | 23,9030 | 0,1183 | 0,0311 | 26,2951 |
| Cluster of Protein transport protein Sec61 subunit alpha isoform | Sec61a1 | 52 | 0,0409 | 0,0141 | 34,4869 | 0,0393 | 0,0086 | 21,9446 |
| procollagen-proline 4-dioxygenase | P4ha2 | 61 | 0,0319 | 0,0106 | 33,2197 | 0,0366 | 0,0118 | 32,2560 |
| PCI domain-containing protein | Psmd11 | 47 | 0,0471 | 0,0189 | 40,1472 | 0,0380 | 0,0204 | 53,7298 |
| Small ribosomal subunit protein | Mrps22 | 41 | 0,0584 | 0,0064 | 11,0238 | 0,0373 | 0,0157 | 41,9931 |
| 3-hydroxyisobutyryl-CoA hydrolase | Hibch | 43 | 0,0734 | 0,0069 | 9,3560 | 0,0063 | 0,0075 | 119,1500 |
| 3-ketoacyl-CoA thiolase | Acaa2 | 42 | 0,0471 | 0,0097 | 20,5816 | 0,0498 | 0,0186 | 37,3571 |
| Endoplasmic reticulum transmembrane helix | Atp13a1 | 132 | 0,0156 | 0,0007 | 4,6620 | 0,0153 | 0,0034 | 22,4233 |
| Heme oxygenase 2 | Hmox2 | 36 | 0,0645 | 0,0180 | 27,9793 | 0,0427 | 0,0183 | 42,8994 |
| Superoxide dismutase [Mn], mitochondrial | Sod2 | 25 | 0,0920 | 0,0175 | 19,0790 | 0,0640 | 0,0135 | 21,0137 |
| Mitochondrial proton/calcium exchanger | Letm1 | 83 | 0,0278 | 0,0059 | 21,3479 | 0,0193 | 0,0041 | 21,0137 |
| Cluster of AAA+ ATPase domain-containing protein | Afg3l2 | 89 | 0,0240 | 0,0030 | 12,6768 | 0,0194 | 0,0047 | 24,4685 |
| Endoplasmic reticulum junction formation protein | Lnpk | 48 | 0,0376 | 0,0098 | 26,1244 | 0,0466 | 0,0150 | 32,2560 |
| RNA-binding protein FXR1 | Fxr1 | 64 | 0,0297 | 0,0082 | 27,4694 | 0,0359 | 0,0107 | 29,9281 |
| GrpE protein homolog 1 | Grpel1 | 24 | 0,0960 | 0,0405 | 42,2537 | 0,0601 | 0,0119 | 19,8798 |
| Peptidyl-prolyl cis-trans | Ppic | 23 | 0,1182 | 0,0234 | 19,8301 | 0,0306 | 0,0214 | 69,9176 |
| Hexokinase-2 | Hk2 | 103 | 0,0206 | 0,0092 | 44,6013 | 0,0145 | 0,0099 | 68,1972 |
| Eukaryotic translation initiation factor 2 subunit | Eif2s1 | 36 | 0,0682 | 0,0253 | 37,1809 | 0,0300 | 0,0207 | 69,0426 |
| Down syndrome cell adhesion molecule-like protein | Dscaml1 | 40 | 0,0664 | 0,0054 | 8,1612 | 0,0225 | 0,0166 | 73,8033 |
| Transmembrane p24 trafficking protein | Tmed7 | 25 | 0,0782 | 0,0240 | 30,7632 | 0,0751 | 0,0552 | 73,5618 |
| Splicing factor 3b, subunit | Sf3b3 | 136 | 0,0232 | 0,0036 | 15,4425 | 0,0000 | 0,0000 |  |
| High mobility group protein | Hmgb2 | 24 | 0,1319 | 0,0130 | 9,8382 | 0,0000 | 0,0000 |  |
| LRP chaperone MESD | Mesd | 25 | 0,1265 | 0,0111 | 8,8124 | 0,0000 | 0,0000 |  |
| peptidylprolyl isomerase | Fkbp8 | 38 | 0,0496 | 0,0092 | 18,4974 | 0,0522 | 0,0195 | 37,4650 |
| Eukaryotic translation initiation factor 3 subunit | Eif3d | 64 | 0,0323 | 0,0057 | 17,7039 | 0,0270 | 0,0066 | 24,4685 |
| Lipase maturation factor 2 | Lmf2 | 80 | 0,0224 | 0,0011 | 5,1030 | 0,0267 | 0,0068 | 25,3151 |
| NADH dehydrogenase [ubiquinone] 1 alpha subcomplex assembly | Ndufaf2 | 20 | 0,0941 | 0,0089 | 9,4117 | 0,1066 | 0,0273 | 25,6371 |
| Sortilin | Sort1 | 91 | 0,0207 | 0,0039 | 18,9950 | 0,0227 | 0,0083 | 36,6186 |
| Actin-related protein 2/3 complex subunit | Arpc1b | 41 | 0,0498 | 0,0104 | 20,9294 | 0,0400 | 0,0178 | 44,5307 |
| A disintegrin and metallopeptidase | Adam17 | 93 | 0,0182 | 0,0042 | 22,9869 | 0,0240 | 0,0078 | 32,2560 |
| TGS domain-containing protein | Mrpl39 | 35 | 0,0564 | 0,0134 | 23,7673 | 0,0530 | 0,0079 | 14,9862 |
| Cluster of Serine/threonine-protein phosphatase PP1-alpha catalytic subunit | Ppp1ca | 38 | 0,0490 | 0,0146 | 29,8294 | 0,0534 | 0,0149 | 27,8766 |
| Electron transfer flavoprotein-ubiquinone oxidoreductase | Etfdh | 68 | 0,0300 | 0,0058 | 19,4209 | 0,0259 | 0,0087 | 33,4900 |
| Thioredoxin domain-containing protein | Txndc12 | 19 | 0,1357 | 0,0266 | 19,5935 | 0,0463 | 0,0155 | 33,4900 |
| Serine/threonine-protein phosphatase | Pgam5 | 32 | 0,0741 | 0,0225 | 30,3145 | 0,0364 | 0,0131 | 36,0478 |
| Coatomer subunit gamma-1 | Copg1 | 98 | 0,0200 | 0,0027 | 13,6448 | 0,0172 | 0,0130 | 76,0086 |
| Pyrroline-5-carboxylate reductase | Pycr3 | 29 | 0,0707 | 0,0166 | 23,4574 | 0,0566 | 0,0385 | 67,9673 |
| Cluster of Cofilin-1 | Cfl1 | 19 | 0,1441 | 0,0252 | 17,5109 | 0,0321 | 0,0076 | 23,5822 |
| Lysosome-associated membrane glycoprotein | Lamp1 | 44 | 0,0331 | 0,0048 | 14,3308 | 0,0623 | 0,0151 | 24,2001 |
| Tudor and KH domain containing | Tdrkh | 62 | 0,0319 | 0,0147 | 46,2036 | 0,0263 | 0,0172 | 65,4464 |
| RRM domain-containing protein | Nono | 60 | 0,0343 | 0,0089 | 25,9501 | 0,0270 | 0,0120 | 44,3607 |
| AB hydrolase-1 domain-containing | Bphl | 34 | 0,0556 | 0,0081 | 14,5175 | 0,0539 | 0,0248 | 46,0917 |
| Cluster of ADP-ribosylation | Arf3 | 21 | 0,0858 | 0,0104 | 12,1826 | 0,0892 | 0,0599 | 67,2051 |
| Glucose-6-phosphate | G6pdx | 59 | 0,0387 | 0,0126 | 32,4361 | 0,0184 | 0,0086 | 46,5178 |
| Peptidyl-prolyl cis-trans | Fkbp4 | 52 | 0,0528 | 0,0099 | 18,7737 | 0,0087 | 0,0064 | 73,8033 |
| U1 small nuclear ribonucleoprotein | Snrpa | 32 | 0,0834 | 0,0185 | 22,2331 | 0,0166 | 0,0203 | 122,7714 |
| Calcyclin-binding protein | Cacybp | 27 | 0,1044 | 0,0104 | 9,9778 | 0,0100 | 0,0119 | 119,1500 |
| 40S ribosomal protein S25 | Rps25 | 10 | 0,1627 | 0,0515 | 31,6345 | 0,2275 | 0,0661 | 29,0568 |
| ATP-dependent Clp protease proteolytic subunit | Clpp | 30 | 0,0344 | 0,0100 | 28,8921 | 0,1054 | 0,0222 | 21,0311 |
| Alkylglycerone-phosphate synthase | Agps | 74 | 0,0221 | 0,0064 | 28,7111 | 0,0297 | 0,0027 | 8,9818 |
| Probable arginine--tRNA ligase | Rars2 | 65 | 0,0252 | 0,0170 | 67,4145 | 0,0322 | 0,0138 | 42,7549 |
| Mitochondrial amidoxime reducing component | Mtarc2 | 38 | 0,0496 | 0,0063 | 12,5908 | 0,0446 | 0,0103 | 23,1452 |
| ELAV-like protein 1 | Elavl1 | 36 | 0,0616 | 0,0107 | 17,3552 | 0,0315 | 0,0068 | 21,4643 |
| 40S ribosomal protein S12 | Rps12 | 16 | 0,1127 | 0,0298 | 26,4724 | 0,1138 | 0,0590 | 51,8893 |
| Fructose-bisphosphate aldolase | Aldob | 40 | 0,0263 | 0,0229 | 86,8738 | 0,0868 | 0,0370 | 42,6568 |
| Transformer 2 beta | Tra2b | 22 | 0,1276 | 0,0236 | 18,4752 | 0,0050 | 0,0101 | 200,0000 |
| Small nuclear ribonucleoprotein Sm | Snrpd1 | 13 | 0,1506 | 0,0280 | 18,5842 | 0,1133 | 0,0158 | 13,9667 |
| Polypeptide N-acetylgalactosaminyltransferase (Fragment | Galnt2 | 61 | 0,0279 | 0,0120 | 42,9980 | 0,0315 | 0,0083 | 26,2706 |
| Eukaryotic translation initiation factor 4, gamma | Eif4g1 | 146 | 0,0112 | 0,0013 | 11,7351 | 0,0144 | 0,0020 | 13,6986 |
| Steryl-sulfatase | Sts | 67 | 0,0255 | 0,0070 | 27,2788 | 0,0274 | 0,0073 | 26,7312 |
| Cluster of Plasma membrane calcium-transporting ATPase | Atp2b1 | 136 | 0,0133 | 0,0020 | 15,1198 | 0,0132 | 0,0033 | 25,1628 |
| Large ribosomal subunit protein | Rpl36a | 12 | 0,1280 | 0,0121 | 9,4467 | 0,1874 | 0,0532 | 28,3997 |
| ER membrane protein complex | Emc1 | 112 | 0,0174 | 0,0056 | 32,3033 | 0,0124 | 0,0041 | 32,8899 |
| Serrate RNA effector molecule | Srrt | 100 | 0,0187 | 0,0057 | 30,4340 | 0,0150 | 0,0061 | 40,6800 |
| Cluster of rRNA 2'-O-methyltransferase | Fbl | 34 | 0,0552 | 0,0169 | 30,6735 | 0,0450 | 0,0189 | 41,9931 |
| 26S proteasome non-ATPase regulatory | Psmd3 | 61 | 0,0348 | 0,0116 | 33,2470 | 0,0187 | 0,0102 | 54,7700 |
| Large ribosomal subunit protein | Mrpl43 | 18 | 0,1190 | 0,0107 | 9,0233 | 0,0563 | 0,0660 | 117,2710 |
| Splicing factor, proline- and glutamine | Sfpq | 75 | 0,0354 | 0,0082 | 23,2466 | 0,0041 | 0,0051 | 124,2177 |
| THO complex subunit 4 | Alyref | 27 | 0,1045 | 0,0024 | 2,2745 | 0,0000 | 0,0000 |  |
| Large ribosomal subunit protein | Rplp2 | 12 | 0,0717 | 0,0182 | 25,4399 | 0,2811 | 0,0991 | 35,2649 |
| Transmembrane protein 33 | Tmem33 | 28 | 0,0611 | 0,0104 | 16,9779 | 0,0623 | 0,0050 | 7,9985 |
| Torsin-1A-interacting protein | Tor1aip1 | 67 | 0,0230 | 0,0034 | 14,7897 | 0,0304 | 0,0147 | 48,3658 |
| NADH dehydrogenase [ubiquinone] 1 alpha subcomplex subunit | Ndufa10 | 41 | 0,0394 | 0,0092 | 23,2347 | 0,0435 | 0,0226 | 51,9687 |
| Reticulocalbin-1 | Rcn1 | 38 | 0,0474 | 0,0058 | 12,1826 | 0,0404 | 0,0173 | 42,8994 |
| Long-chain fatty acid transport protein | Slc27a4 | 72 | 0,0216 | 0,0070 | 32,5338 | 0,0277 | 0,0045 | 16,1478 |
| Surfeit locus protein 4 | Surf4 | 30 | 0,0486 | 0,0070 | 14,3308 | 0,0737 | 0,0120 | 16,3012 |
| Cytochrome P450 2J6 | Cyp2j6 | 58 | 0,0251 | 0,0036 | 14,3308 | 0,0373 | 0,0074 | 19,8798 |
| 26S proteasome regulatory subunit | Psmc1 | 49 | 0,0363 | 0,0126 | 34,7396 | 0,0325 | 0,0171 | 52,6534 |
| Armadillo repeat-containing protein | Armc10 | 33 | 0,0492 | 0,0045 | 9,0958 | 0,0540 | 0,0281 | 51,9687 |
| Reticulocalbin 3, EF-hand calcium binding domain | Rcn3 | 32 | 0,0587 | 0,0181 | 30,9258 | 0,0456 | 0,0190 | 41,6818 |
| Elongation factor Ts, mitochondrial | Tsfm | 36 | 0,0638 | 0,0120 | 18,8491 | 0,0176 | 0,0151 | 85,8524 |
| Lysine--tRNA ligase | Kars1 | 68 | 0,0326 | 0,0035 | 10,5881 | 0,0110 | 0,0109 | 99,6249 |
| Nucleolar protein 56 | Nop56 | 64 | 0,0330 | 0,0121 | 36,6411 | 0,0141 | 0,0122 | 86,1335 |
| Sec1 family domain-containing | Scfd1 | 72 | 0,0238 | 0,0043 | 18,0232 | 0,0202 | 0,0244 | 120,7865 |
| Cluster of Serine/arginine-rich splicing | Srsf6 | 39 | 0,0638 | 0,0066 | 10,4117 | 0,0120 | 0,0088 | 73,2090 |
| High mobility group box | Hmgb1 | 24 | 0,1135 | 0,0184 | 16,1832 | 0,0000 | 0,0000 |  |
| Receptor expression-enhancing protein | Reep5 | 21 | 0,0653 | 0,0030 | 4,6620 | 0,1094 | 0,0327 | 29,9281 |
| Translocation protein SEC62 (Fragment | Sec62 | 16 | 0,0801 | 0,0094 | 11,7799 | 0,1482 | 0,0383 | 25,8721 |
| Thioredoxin-related transmembrane protein | Tmx2 | 30 | 0,0540 | 0,0164 | 30,3039 | 0,0554 | 0,0230 | 41,4123 |
| Ceramide synthase 5 | Cers5 | 48 | 0,0269 | 0,0046 | 17,1016 | 0,0494 | 0,0128 | 25,8721 |
| Low density lipoprotein receptor | Ldlr | 89 | 0,0152 | 0,0065 | 42,7528 | 0,0254 | 0,0100 | 39,1551 |
| Serum paraoxonase/arylesterase 2 | Pon2 | 40 | 0,0386 | 0,0114 | 29,4936 | 0,0472 | 0,0074 | 15,7573 |
| Nucleoporin NDC1 | Ndc1 | 75 | 0,0239 | 0,0036 | 15,1198 | 0,0185 | 0,0061 | 32,8899 |
| DNA replication licensing factor | Mcm3 | 92 | 0,0187 | 0,0037 | 19,8150 | 0,0172 | 0,0024 | 13,9747 |
| Outer mitochondrial transmembrane helix | Atad1 | 41 | 0,0415 | 0,0272 | 65,4595 | 0,0385 | 0,0160 | 41,5758 |
| Mannosyl-oligosaccharide glucosidase | Mogs | 92 | 0,0223 | 0,0059 | 26,4226 | 0,0093 | 0,0103 | 111,0676 |
| Small nuclear ribonucleoprotein-associated | Snrpb | 24 | 0,0890 | 0,0033 | 3,7586 | 0,0324 | 0,0248 | 76,5298 |
| Cluster of ADP-ribosylation factor-like | Arl8b | 16 | 0,1172 | 0,0244 | 20,8222 | 0,0728 | 0,0626 | 86,0020 |
| 14-3-3 protein zeta | Ywhaz | 28 | 0,0797 | 0,0142 | 17,8644 | 0,0130 | 0,0157 | 120,7865 |
| Cluster of Proteasome subunit alpha | Psma7 | 28 | 0,0861 | 0,0176 | 20,3912 | 0,0136 | 0,0161 | 118,5050 |
| Tropomyosin alpha-4 chain | Tpm4 | 28 | 0,0946 | 0,0103 | 10,8656 | 0,0000 | 0,0000 |  |
| FAM3 metabolism regulating signaling molecule | Fam3c | 21 | 0,0650 | 0,0177 | 27,2525 | 0,0974 | 0,0214 | 21,9446 |
| Cluster of ATP-dependent 6-phosphofructokinase, muscle | Pfkm | 85 | 0,0211 | 0,0075 | 35,3880 | 0,0159 | 0,0067 | 42,3507 |
| t-SNARE coiled-coil homology domain-containing | Stx12 | 31 | 0,0526 | 0,0199 | 37,7334 | 0,0511 | 0,0071 | 13,9747 |
| Plexin-B2 | Plxnb2 | 207 | 0,0062 | 0,0026 | 41,3708 | 0,0097 | 0,0056 | 57,1193 |
| Signal recognition particle subunit | Srp54 | 56 | 0,0277 | 0,0084 | 30,2025 | 0,0318 | 0,0080 | 25,1628 |
| Cadherin 2 | Cdh2 | 94 | 0,0174 | 0,0025 | 14,4563 | 0,0159 | 0,0065 | 40,6800 |
| Delta-1-pyrroline-5-carboxylate dehydrogenase, mitochondrial | Aldh4a1 | 62 | 0,0235 | 0,0036 | 15,1461 | 0,0302 | 0,0111 | 36,6996 |
| Agenet-like domain-containing protein | Fxr2 | 71 | 0,0232 | 0,0122 | 52,4800 | 0,0233 | 0,0108 | 46,2982 |
| RRM domain-containing protein | Hnrnpm | 72 | 0,0261 | 0,0129 | 49,2776 | 0,0145 | 0,0104 | 71,5447 |
| Discs large MAGUK scaffold | Dlg1 | 101 | 0,0212 | 0,0019 | 9,0233 | 0,0063 | 0,0054 | 85,8524 |
| Amine oxidase (Fragment | Maoa | 59 | 0,0293 | 0,0117 | 39,9035 | 0,0215 | 0,0252 | 117,2710 |
| Uncharacterized protein | Mapre1 | 30 | 0,0714 | 0,0064 | 9,0233 | 0,0217 | 0,0251 | 115,5201 |
| Heterogeneous nuclear ribonucleoprotein D0 | Hnrnpd | 38 | 0,0653 | 0,0087 | 13,3370 | 0,0038 | 0,0075 | 200,0000 |
| Protein lin-7 homolog | Lin7c | 22 | 0,1169 | 0,0117 | 10,0282 | 0,0000 | 0,0000 |  |
| Ras-related protein Rab | Rab21 | 27 | 0,0502 | 0,0219 | 43,6039 | 0,0737 | 0,0211 | 28,5818 |
| Transmembrane emp24 domain trafficking | Tmed2 | 23 | 0,0628 | 0,0208 | 33,0818 | 0,0782 | 0,0220 | 28,1499 |
| Cytochrome c oxidase subunit | COX2 | 26 | 0,0528 | 0,0117 | 22,1213 | 0,0725 | 0,0114 | 15,7573 |
| Aspartate--tRNA ligase, cytoplasmic | Dars1 | 57 | 0,0316 | 0,0038 | 12,1826 | 0,0208 | 0,0076 | 36,3893 |
| CDK5 regulatory subunit-associated protein | Cdk5rap3 | 57 | 0,0301 | 0,0075 | 24,7343 | 0,0218 | 0,0072 | 32,7663 |
| Peptidyl-prolyl cis-trans | Fkbp11 | 22 | 0,0743 | 0,0175 | 23,5366 | 0,0638 | 0,0241 | 37,7696 |
| Large ribosomal subunit protein | Mrpl37 | 48 | 0,0371 | 0,0115 | 30,9697 | 0,0243 | 0,0088 | 36,0478 |
| Solute carrier family 12, member | Slc12a2 | 131 | 0,0124 | 0,0012 | 10,0188 | 0,0106 | 0,0035 | 32,8899 |
| Proteasome (prosome, macropain) 26S subunit | Psmc3 | 45 | 0,0319 | 0,0125 | 39,2935 | 0,0368 | 0,0137 | 37,1190 |
| ADP-ribosylation factor GTPase-activating | Arfgap1 | 45 | 0,0359 | 0,0137 | 38,2266 | 0,0290 | 0,0206 | 70,8821 |
| Putative divalent cation/proton | Tmem165 | 35 | 0,0440 | 0,0051 | 11,5581 | 0,0442 | 0,0146 | 33,1227 |
| 26S proteasome regulatory subunit | Psmc4 | 47 | 0,0292 | 0,0067 | 22,9094 | 0,0387 | 0,0163 | 42,2427 |
| Cluster of Cyclin-dependent | Cdk1 | 34 | 0,0428 | 0,0053 | 12,3979 | 0,0392 | 0,0288 | 73,4444 |
| Splicing factor 3b, subunit | Sf3b1 | 147 | 0,0121 | 0,0054 | 44,9018 | 0,0068 | 0,0064 | 93,7600 |
| Stalled ribosome sensor GCN1 | Gcn1 | 293 | 0,0067 | 0,0021 | 31,4014 | 0,0027 | 0,0025 | 94,3826 |
| Nucleoside diphosphate kinase (Fragment | Nme4 | 22 | 0,0929 | 0,0146 | 15,7570 | 0,0296 | 0,0342 | 115,5201 |
| Excitatory amino acid transporter | Slc1a3 | 60 | 0,0199 | 0,0054 | 27,1745 | 0,0364 | 0,0137 | 37,6247 |
| Protein transport protein Sec61 subunit beta | Sec61b | 10 | 0,1377 | 0,0516 | 37,5041 | 0,1853 | 0,1057 | 57,0315 |
| Chloride channel protein | Clcn5 | 83 | 0,0174 | 0,0088 | 50,8677 | 0,0197 | 0,0029 | 14,9888 |
| Transmembrane emp24 domain-containing | Tmed3 | 25 | 0,0616 | 0,0076 | 12,4202 | 0,0542 | 0,0244 | 44,9772 |
| Galectin | Lgals9 | 40 | 0,0405 | 0,0067 | 16,4508 | 0,0307 | 0,0149 | 48,6864 |
| Regulator of nonsense transcripts | Upf1 | 124 | 0,0131 | 0,0033 | 25,4212 | 0,0107 | 0,0046 | 43,0294 |
| RRM domain-containing protein | Hnrnpc | 37 | 0,0578 | 0,0084 | 14,5276 | 0,0359 | 0,0325 | 90,5329 |
| Propionyl-CoA carboxylase alpha chain | Pcca | 80 | 0,0212 | 0,0060 | 28,4275 | 0,0121 | 0,0146 | 120,3954 |
| Thymopoietin | Tmpo | 46 | 0,0390 | 0,0093 | 23,7314 | 0,0182 | 0,0231 | 126,7717 |
| Cold shock domain-containing | Csde1 | 89 | 0,0210 | 0,0042 | 20,0066 | 0,0084 | 0,0117 | 139,1106 |
| Eukaryotic translation initiation factor 3 subunit | Eif3b | 110 | 0,0205 | 0,0024 | 11,7211 | 0,0025 | 0,0030 | 119,1500 |
| Creatine kinase B-type | Ckb | 43 | 0,0520 | 0,0115 | 22,1059 | 0,0059 | 0,0069 | 117,2710 |
| Cluster of Tyrosine 3-monooxygenase/tryptophan 5-monooxygenase activation protein, beta | Ywhab | 28 | 0,0733 | 0,0098 | 13,3686 | 0,0000 | 0,0000 |  |
| Histocompatibility 13 (Fragment | H13 | 38 | 0,0381 | 0,0079 | 20,7264 | 0,0392 | 0,0088 | 22,4077 |
| Transmembrane protein 263 | Tmem263 | 12 | 0,0934 | 0,0295 | 31,5716 | 0,1782 | 0,0569 | 31,9503 |
| IKBKB interacting protein | Ikbip | 42 | 0,0286 | 0,0052 | 18,3076 | 0,0453 | 0,0105 | 23,0998 |
| ENTH domain-containing protein | Clint1 | 68 | 0,0213 | 0,0085 | 40,0741 | 0,0212 | 0,0042 | 19,8798 |
| Cell division control protein | Cdc42 | 21 | 0,0605 | 0,0223 | 36,9314 | 0,0830 | 0,0066 | 7,9985 |
| Rab9A | Rab9 | 23 | 0,0631 | 0,0137 | 21,6247 | 0,0641 | 0,0089 | 13,9667 |
| Alpha-globin | Hbat1 | 15 | 0,0808 | 0,0322 | 39,8573 | 0,1340 | 0,0595 | 44,3641 |
| Caveolae-associated protein 1 | Cavin1 | 44 | 0,0329 | 0,0064 | 19,4361 | 0,0304 | 0,0171 | 56,2847 |
| Dynamin-like 120 kDa protein | Opa1 | 113 | 0,0136 | 0,0028 | 20,6796 | 0,0113 | 0,0033 | 29,6498 |
| NADH dehydrogenase [ubiquinone] 1 beta subcomplex subunit | Ndufb4 | 15 | 0,1079 | 0,0376 | 34,8333 | 0,0756 | 0,0162 | 21,4643 |
| Large ribosomal subunit protein | Mrpl46 | 32 | 0,0508 | 0,0046 | 9,0958 | 0,0359 | 0,0238 | 66,3488 |
| CDGSH iron sulfur domain | Cisd2 | 12 | 0,1425 | 0,0221 | 15,5260 | 0,0857 | 0,0418 | 48,7495 |
| Medium-chain acyl-CoA ligase | Acsf2 | 68 | 0,0224 | 0,0089 | 39,9016 | 0,0181 | 0,0137 | 75,6735 |
| Mitochondrial import inner membrane translocase | Timm23 | 22 | 0,0660 | 0,0192 | 29,1168 | 0,0628 | 0,0455 | 72,4510 |
| Proteasome subunit alpha type | Psma6 | 27 | 0,0629 | 0,0181 | 28,8038 | 0,0353 | 0,0311 | 88,1517 |
| Coiled-coil-helix-coiled-coil-helix domain-containing | Chchd2 | 16 | 0,1333 | 0,0163 | 12,2161 | 0,0169 | 0,0201 | 119,1500 |
| Spectrin beta chain, non | Sptbn1 | 274 | 0,0081 | 0,0012 | 14,6502 | 0,0005 | 0,0010 | 200,0000 |
| Treacle ribosome biogenesis factor | Tcof1 | 139 | 0,0166 | 0,0046 | 27,6951 | 0,0000 | 0,0000 |  |
| Cluster of Alpha-2-macroglobulin receptor-associated | Lrpap1 | 44 | 0,0524 | 0,0091 | 17,3826 | 0,0000 | 0,0000 |  |
| TAR DNA-binding protein | Tardbp | 27 | 0,0476 | 0,0068 | 14,3511 | 0,0625 | 0,0207 | 33,1453 |
| Collagen alpha-1(VI | Col6a1 | 108 | 0,0143 | 0,0032 | 22,4277 | 0,0113 | 0,0027 | 23,5822 |
| Myeloid-associated differentiation marker | Myadm | 35 | 0,0368 | 0,0099 | 26,7861 | 0,0467 | 0,0070 | 14,9888 |
| NADH dehydrogenase [ubiquinone] 1 alpha subcomplex | Ndufa6 | 15 | 0,0965 | 0,0188 | 19,4361 | 0,0866 | 0,0351 | 40,5800 |
| Large neutral amino acids transporter small subunit | Slc7a5 | 56 | 0,0215 | 0,0067 | 30,9791 | 0,0314 | 0,0105 | 33,4900 |
| GTP:AMP phosphotransferase AK3 | Ak3 | 21 | 0,0659 | 0,0248 | 37,6769 | 0,0684 | 0,0235 | 34,4467 |
| Cluster of Solute carrier family 12, member | Slc12a4 | 121 | 0,0120 | 0,0015 | 12,3979 | 0,0103 | 0,0034 | 32,7663 |
| Synaptosomal-associated protein | Snap23 | 23 | 0,0635 | 0,0096 | 15,1461 | 0,0595 | 0,0316 | 53,0706 |
| Malate dehydrogenase, cytoplasmic | Mdh1 | 37 | 0,0461 | 0,0059 | 12,7534 | 0,0248 | 0,0124 | 50,1246 |
| Large ribosomal subunit protein | Mrps30 | 50 | 0,0294 | 0,0113 | 38,5725 | 0,0260 | 0,0105 | 40,5800 |
| Nestin | Nes | 207 | 0,0079 | 0,0019 | 24,0526 | 0,0047 | 0,0015 | 32,5529 |
| Arginine--tRNA ligase, cytoplasmic | Rars1 | 76 | 0,0201 | 0,0071 | 35,1838 | 0,0147 | 0,0065 | 44,3368 |
| Mrpl13 protein (Fragment | Mrpl13 | 20 | 0,0728 | 0,0090 | 12,3979 | 0,0662 | 0,0285 | 43,0294 |
| Endoplasmic reticulum metallopeptidase 1 | Ermp1 | 100 | 0,0153 | 0,0041 | 26,7521 | 0,0110 | 0,0080 | 73,1083 |
| ATP-dependent RNA helicase | Ddx46 | 118 | 0,0132 | 0,0030 | 22,9268 | 0,0099 | 0,0067 | 68,0558 |
| Dedicator of cytokinesis 7 | Dock7 | 238 | 0,0068 | 0,0041 | 60,2233 | 0,0039 | 0,0032 | 82,3271 |
| Mitochondrial Rho GTPase 2 | Rhot2 | 69 | 0,0236 | 0,0051 | 21,7776 | 0,0131 | 0,0113 | 86,1335 |
| SAM domain-containing protein | Ppfibp1 | 109 | 0,0140 | 0,0047 | 33,2881 | 0,0089 | 0,0107 | 120,3954 |
| Heterogeneous nuclear ribonucleoprotein A3 | Hnrnpa3 | 34 | 0,0649 | 0,0181 | 27,9138 | 0,0000 | 0,0000 | #DIV/0! |
| Cytochrome c oxidase subunit | Cox6b1 | 10 | 0,2238 | 0,0426 | 19,0346 | 0,0000 | 0,0000 | #DIV/0! |
| Cluster of Lysosome-associated membrane | Lamp2 | 46 | 0,0276 | 0,0102 | 36,9314 | 0,0324 | 0,0073 | 22,4077 |
| ATP synthase subunit epsilon | Atp5f1e | 6 | 0,2153 | 0,0617 | 28,6508 | 0,2403 | 0,0478 | 19,8798 |
| Small nuclear ribonucleoprotein Sm | Snrpd2 | 14 | 0,1099 | 0,0127 | 11,5581 | 0,0758 | 0,0286 | 37,8164 |
| Rho-related GTP-binding protein | Rhog | 21 | 0,0691 | 0,0150 | 21,6247 | 0,0525 | 0,0255 | 48,6242 |
| NADH:ubiquinone oxidoreductase subunit | Ndufa9 | 42 | 0,0301 | 0,0122 | 40,6310 | 0,0330 | 0,0109 | 32,8899 |
| NADH dehydrogenase [ubiquinone] iron-sulfur protein | Ndufs2 | 53 | 0,0259 | 0,0012 | 4,6620 | 0,0241 | 0,0071 | 29,6498 |
| Oxidoreductase NAD-binding domain-containing | Oxnad1 | 35 | 0,0390 | 0,0215 | 55,1540 | 0,0369 | 0,0134 | 36,3730 |
| Proteasome subunit alpha type | Psma1 | 30 | 0,0538 | 0,0125 | 23,2347 | 0,0298 | 0,0187 | 62,7759 |
| Mitochondrial Rho GTPase 1 | Rhot1 | 72 | 0,0178 | 0,0026 | 14,3511 | 0,0193 | 0,0063 | 32,8899 |
| SAP domain-containing protein | Sf3b2 | 98 | 0,0165 | 0,0025 | 15,2662 | 0,0079 | 0,0061 | 76,5298 |
| Enoyl-Coenzyme A delta | Eci2 | 42 | 0,0347 | 0,0043 | 12,3979 | 0,0257 | 0,0177 | 69,0426 |
| Cluster of Small ribosomal subunit protein | Dap3 | 42 | 0,0346 | 0,0075 | 21,6247 | 0,0249 | 0,0178 | 71,5447 |
| Aladin | Aaas | 59 | 0,0260 | 0,0068 | 26,2612 | 0,0168 | 0,0063 | 37,4650 |
| Sperm antigen with calponin homology and coiled-coil | Specc1 | 118 | 0,0146 | 0,0007 | 4,6620 | 0,0058 | 0,0046 | 78,9119 |
| Tumor protein D52-like | Tpd52l2 | 25 | 0,0649 | 0,0120 | 18,4730 | 0,0324 | 0,0283 | 87,5237 |
| Dehydrogenase/reductase SDR family | Dhrs13 | 41 | 0,0396 | 0,0036 | 9,0958 | 0,0182 | 0,0181 | 99,6249 |
| Small nuclear ribonucleoprotein Sm | Snrpd3 | 14 | 0,1524 | 0,0193 | 12,6768 | 0,0000 | 0,0000 |  |
| Spectrin alpha, non-erythrocytic | Sptan1 | 283 | 0,0075 | 0,0026 | 35,2975 | 0,0000 | 0,0000 |  |
| CCHC-type domain-containing | Cnbp | 19 | 0,1128 | 0,0121 | 10,7508 | 0,0000 | 0,0000 |  |
| Cluster of Equilibrative nucleoside transporter 1 variant delta | Slc29a1 | 39 | 0,0264 | 0,0129 | 48,8087 | 0,0455 | 0,0106 | 23,2860 |
| Neuroplastin | Nptn | 44 | 0,0309 | 0,0050 | 16,1832 | 0,0270 | 0,0098 | 36,3893 |
| Cell adhesion molecule 1 | Cadm1 | 43 | 0,0258 | 0,0027 | 10,5881 | 0,0380 | 0,0057 | 14,9888 |
| Vacuolar proton pump subunit | Atp6v1b2 | 59 | 0,0201 | 0,0047 | 23,2912 | 0,0239 | 0,0116 | 48,4739 |
| Inactive rhomboid protein 1 | Rhbdf1 | 97 | 0,0124 | 0,0035 | 28,6648 | 0,0148 | 0,0051 | 34,4467 |
| Large ribosomal subunit protein | Rpl38 | 8 | 0,1713 | 0,0080 | 4,6620 | 0,1377 | 0,0670 | 48,6242 |
| Bone marrow stromal antigen | Bst2 | 19 | 0,0320 | 0,0184 | 57,3864 | 0,1359 | 0,0312 | 22,9749 |
| Small ribosomal subunit protein | Rps7 | 22 | 0,0582 | 0,0192 | 33,0678 | 0,0566 | 0,0185 | 32,7663 |
| E3 ubiquitin-protein ligase | Amfr | 73 | 0,0154 | 0,0049 | 32,0486 | 0,0227 | 0,0113 | 49,8454 |
| D-beta-hydroxybutyrate dehydrogenase | Bdh1 | 38 | 0,0380 | 0,0126 | 33,0818 | 0,0248 | 0,0148 | 59,5449 |
| GPI transamidase component PIG | Pigs | 62 | 0,0262 | 0,0116 | 44,2065 | 0,0116 | 0,0023 | 19,8798 |
| Sel-1 suppressor of lin-12-like (C. elegans | Sel1l | 82 | 0,0185 | 0,0099 | 53,4193 | 0,0101 | 0,0042 | 41,4123 |
| Cluster of GTP-binding protein SAR1a | Sar1a | 22 | 0,0503 | 0,0230 | 45,7812 | 0,0742 | 0,0111 | 14,9888 |
| Hexose-6-phosphate dehydrogenase (glucose | H6pd | 90 | 0,0162 | 0,0023 | 14,3308 | 0,0105 | 0,0062 | 59,5449 |
| Pre-mRNA-processing-splicing | Prpf8 | 274 | 0,0047 | 0,0018 | 38,4258 | 0,0045 | 0,0031 | 69,4697 |
| Glutathione peroxidase | Gpx7 | 21 | 0,0767 | 0,0315 | 41,0548 | 0,0325 | 0,0257 | 78,9119 |
| Cytochrome P450 20A1 | Cyp20a1 | 52 | 0,0279 | 0,0123 | 43,9496 | 0,0186 | 0,0140 | 75,1943 |
| Psap protein | Psap | 61 | 0,0225 | 0,0010 | 4,6620 | 0,0174 | 0,0160 | 92,0207 |
| GDP-fucose protein | Pofut2 | 49 | 0,0281 | 0,0067 | 23,7423 | 0,0220 | 0,0152 | 69,0426 |
| Cluster of AP-2 complex subunit alpha | Ap2a2 | 104 | 0,0148 | 0,0071 | 47,8345 | 0,0076 | 0,0061 | 79,6432 |
| DnaJ homolog subfamily | Dnajb11 | 41 | 0,0438 | 0,0145 | 33,1017 | 0,0114 | 0,0083 | 73,2090 |
| Branched-chain-amino-acid | Bcat2 | 43 | 0,0336 | 0,0158 | 47,0711 | 0,0227 | 0,0074 | 32,5529 |
| Alpha | Alg2 | 47 | 0,0346 | 0,0035 | 10,0188 | 0,0142 | 0,0104 | 73,4444 |
| U5 small nuclear ribonucleoprotein 200 kDa | Snrnp200 | 246 | 0,0067 | 0,0025 | 36,8313 | 0,0026 | 0,0022 | 85,8524 |
| Utrophin | Utrn | 393 | 0,0039 | 0,0009 | 24,3214 | 0,0018 | 0,0027 | 146,5962 |
| Keratin 27 | Krt27 | 49 | 0,0000 | 0,0000 | #DIV/0! | 0,0195 | 0,0390 | 200,0000 |
| Protein SCO2 homolog, mitochondrial | Sco2 | 29 | 0,0384 | 0,0061 | 15,8082 | 0,0508 | 0,0071 | 13,9667 |
| Ceramide synthase 6 | Cers6 | 46 | 0,0223 | 0,0010 | 4,6620 | 0,0355 | 0,0053 | 14,9888 |
| 26S proteasome non-ATPase regulatory | Psmd2 | 100 | 0,0136 | 0,0035 | 25,4185 | 0,0097 | 0,0032 | 32,5529 |
| Carnitine O-palmitoyltransferase 2 | Cpt2 | 74 | 0,0162 | 0,0080 | 49,2512 | 0,0188 | 0,0103 | 54,6009 |
| Retinol dehydrogenase 11 | Rdh11 | 35 | 0,0342 | 0,0051 | 15,0531 | 0,0369 | 0,0134 | 36,3730 |
| Triosephosphate isomerase | Tpi1 | 27 | 0,0601 | 0,0156 | 25,9242 | 0,0226 | 0,0053 | 23,5822 |
| Thyroid hormone receptor-associated | Thrap3 | 108 | 0,0143 | 0,0042 | 29,2083 | 0,0062 | 0,0045 | 73,4444 |
| Apolipoprotein B (Fragment | Apob | 504 | 0,0017 | 0,0013 | 72,3643 | 0,0044 | 0,0047 | 105,7931 |
| Galectin-3-binding protein | Lgals3bp | 64 | 0,0226 | 0,0057 | 25,4539 | 0,0127 | 0,0092 | 72,1221 |
| Cluster of GTPase HRas | Hras | 21 | 0,0614 | 0,0294 | 47,8344 | 0,0482 | 0,0332 | 68,9691 |
| Acyl-CoA synthetase long-chain family | Acsl3 | 63 | 0,0244 | 0,0050 | 20,6796 | 0,0101 | 0,0086 | 85,8524 |
| Eukaryotic peptide chain release factor | Etf1 | 49 | 0,0367 | 0,0069 | 18,7069 | 0,0052 | 0,0061 | 117,2710 |
| 14-3-3 protein gamma | Ywhag | 28 | 0,0578 | 0,0137 | 23,7965 | 0,0000 | 0,0000 |  |
| 40S ribosomal protein S24 | Rps24 | 14 | 0,0789 | 0,0209 | 26,4746 | 0,0951 | 0,0130 | 13,7199 |
| Solute carrier family 2, facilitated glucose transporter member | Slc2a1 | 54 | 0,0205 | 0,0022 | 10,5881 | 0,0246 | 0,0034 | 13,7199 |
| Holocytochrome c-type synthase | Hccs | 31 | 0,0331 | 0,0089 | 26,7939 | 0,0475 | 0,0066 | 13,9667 |
| Reticulon | Rtn1 | 84 | 0,0112 | 0,0019 | 16,5913 | 0,0201 | 0,0067 | 33,1453 |
| Estradiol 17-beta-dehydrogenase | Hsd17b11 | 33 | 0,0362 | 0,0047 | 13,0306 | 0,0344 | 0,0074 | 21,4643 |
| Mitochondrial ribosomal protein L2 | Mrpl2 | 33 | 0,0362 | 0,0134 | 37,0290 | 0,0360 | 0,0131 | 36,3893 |
| 26S proteasome non-ATPase regulatory | Psmd13 | 43 | 0,0260 | 0,0046 | 17,8644 | 0,0306 | 0,0111 | 36,3532 |
| Carboxypeptidase D | Cpd | 152 | 0,0074 | 0,0024 | 32,0486 | 0,0088 | 0,0012 | 13,7199 |
| cathepsin X | Ctsz | 34 | 0,0279 | 0,0106 | 37,8956 | 0,0455 | 0,0151 | 33,1227 |
| Sorting nexin-1 | Snx1 | 59 | 0,0231 | 0,0037 | 16,1832 | 0,0141 | 0,0058 | 41,4123 |
| Eukaryotic translation initiation factor 3 subunit | Eif3l | 67 | 0,0163 | 0,0105 | 64,6675 | 0,0196 | 0,0071 | 36,3532 |
| U1 small nuclear ribonucleoprotein 70 k | Snrnp70 | 52 | 0,0281 | 0,0071 | 25,3943 | 0,0139 | 0,0028 | 19,8798 |
| Uncharacterized protein | Actr3 | 47 | 0,0274 | 0,0076 | 27,7133 | 0,0207 | 0,0067 | 32,5529 |
| Gelsolin | Gsn | 86 | 0,0167 | 0,0052 | 30,8393 | 0,0084 | 0,0017 | 19,8798 |
| Srp72 protein (Fragment | Srp72 | 63 | 0,0205 | 0,0060 | 29,3755 | 0,0141 | 0,0119 | 84,7412 |
| Persulfide dioxygenase ETHE1, mitochondrial | Ethe1 | 28 | 0,0425 | 0,0144 | 33,9470 | 0,0385 | 0,0266 | 69,0426 |
| Cluster of glycerol kinase | Gk | 60 | 0,0199 | 0,0026 | 13,0306 | 0,0194 | 0,0070 | 36,0478 |
| Receptor expression-enhancing protein | Reep4 | 30 | 0,0430 | 0,0074 | 17,1016 | 0,0325 | 0,0106 | 32,5529 |
| Receptor tyrosine-protein kinase | Erbb3 | 148 | 0,0097 | 0,0055 | 56,7636 | 0,0047 | 0,0037 | 79,3941 |
| Serine/threonine kinase receptor associated | Strap | 38 | 0,0337 | 0,0111 | 33,0678 | 0,0225 | 0,0250 | 111,0676 |
| Serine/arginine-rich splicing | Srsf5 | 31 | 0,0582 | 0,0154 | 26,4724 | 0,0046 | 0,0092 | 200,0000 |
| A kinase (PRKA) anchor protein | Akap12 | 181 | 0,0099 | 0,0018 | 18,1241 | 0,0006 | 0,0012 | 200,0000 |
| 14-3-3 protein | Ywhah | 28 | 0,0551 | 0,0162 | 29,4936 | 0,0000 | 0,0000 |  |
| Peptidyl-prolyl cis-trans | Fkbp2 | 15 | 0,1260 | 0,0183 | 14,5175 | 0,0000 | 0,0000 |  |
| Small ribosomal subunit protein | Rps28 | 8 | 0,2370 | 0,0513 | 21,6689 | 0,0000 | 0,0000 |  |
| Galectin-3 | Lgals3 | 28 | 0,0671 | 0,0117 | 17,4028 | 0,0000 | 0,0000 |  |
| Vacuolar protein sorting-associated | Vps45 | 65 | 0,0146 | 0,0032 | 21,6689 | 0,0221 | 0,0076 | 34,4467 |
| Small ribosomal subunit protein | Rps26 | 13 | 0,0859 | 0,0161 | 18,7423 | 0,0896 | 0,0323 | 36,0478 |
| Large ribosomal subunit protein | Mrpl22 | 24 | 0,0425 | 0,0099 | 23,4088 | 0,0552 | 0,0238 | 43,0294 |
| Vesicle-associated membrane protein | Vamp7 | 25 | 0,0345 | 0,0150 | 43,4972 | 0,0646 | 0,0185 | 28,6260 |
| PRA1 family protein 3 | Arl6ip5 | 22 | 0,0426 | 0,0141 | 33,2386 | 0,0670 | 0,0094 | 13,9667 |
| Cluster of Actin-related protein 2/3 complex | Arpc2 | 34 | 0,0330 | 0,0203 | 61,4498 | 0,0333 | 0,0072 | 21,4643 |
| Neurogenic locus notch homolog | Notch1 | 271 | 0,0038 | 0,0011 | 29,0216 | 0,0045 | 0,0022 | 48,6864 |
| Nicastrin | Ncstn | 78 | 0,0153 | 0,0023 | 15,0531 | 0,0125 | 0,0041 | 32,5529 |
| Membrane protein, palmitoylated 6 (MAGUK p55 subfamily | Pals2 | 61 | 0,0210 | 0,0055 | 26,1769 | 0,0136 | 0,0056 | 41,4123 |
| Developmentally-regulated GTP-binding | Drg1 | 41 | 0,0312 | 0,0105 | 33,7202 | 0,0211 | 0,0052 | 24,4685 |
| Cluster of S-adenosylhomocysteine hydrolase-like | Ahcyl1 | 59 | 0,0203 | 0,0031 | 15,0531 | 0,0171 | 0,0082 | 47,8143 |
| Mitochondrial dicarboxylate carrier | Slc25a10 | 32 | 0,0400 | 0,0095 | 23,8496 | 0,0260 | 0,0108 | 41,4123 |
| Secretory carrier-associated membrane | Scamp1 | 38 | 0,0337 | 0,0080 | 23,8496 | 0,0204 | 0,0156 | 76,5298 |
| Mitochondrial ribosomal protein L4 | Mrpl4 | 25 | 0,0446 | 0,0080 | 17,8644 | 0,0438 | 0,0320 | 73,1083 |
| Type I epidermal keratin |  | 11 | 0,0614 | 0,0546 | 88,9458 | 0,1657 | 0,0583 | 35,1841 |
| ATP-dependent zinc metalloprotease | Yme1l1 | 80 | 0,0173 | 0,0067 | 38,4003 | 0,0083 | 0,0061 | 73,4444 |
| RNA helicase | Ddx23 | 95 | 0,0135 | 0,0017 | 12,6634 | 0,0091 | 0,0022 | 24,4685 |
| Acyl-CoA synthetase long-chain family | Acsl4 | 74 | 0,0183 | 0,0067 | 36,6643 | 0,0086 | 0,0074 | 85,8524 |
| ATP-dependent RNA helicase | Dhx15 | 91 | 0,0132 | 0,0041 | 30,9791 | 0,0102 | 0,0081 | 79,8766 |
| Heterogeneous nuclear ribonucleoprotein L | Hnrnpl | 67 | 0,0177 | 0,0059 | 33,4195 | 0,0140 | 0,0094 | 67,2051 |
| Proteasome subunit alpha type | Psma4 | 28 | 0,0516 | 0,0131 | 25,4539 | 0,0187 | 0,0134 | 71,5447 |
| Cadherin-6 | Cdh6 | 88 | 0,0165 | 0,0012 | 7,1684 | 0,0054 | 0,0072 | 132,6393 |
| Cluster of MICOS complex | Immt | 76 | 0,0189 | 0,0083 | 43,7801 | 0,0076 | 0,0052 | 68,9748 |
| Caprin-1 dimerization domain-containing protein | Caprin1 | 38 | 0,0408 | 0,0125 | 30,5174 | 0,0096 | 0,0116 | 120,7865 |
| 116 kDa U5 small nuclear ribonucleoprotein | Eftud2 | 109 | 0,0117 | 0,0037 | 31,8009 | 0,0049 | 0,0060 | 122,7714 |
| Serpine1 mRNA binding protein | Serbp1 | 44 | 0,0389 | 0,0060 | 15,5260 | 0,0036 | 0,0072 | 200,0000 |
| Protein NipSnap homolog 1 | Nipsnap1 | 33 | 0,0284 | 0,0047 | 16,5913 | 0,0413 | 0,0111 | 26,8566 |
| Ectonucleoside triphosphate diphosphohydrolase 2 | Entpd2 | 54 | 0,0173 | 0,0058 | 33,2386 | 0,0242 | 0,0126 | 52,1469 |
| UDP-glucose 6-dehydrogenase | Ugdh | 55 | 0,0187 | 0,0009 | 4,6620 | 0,0213 | 0,0072 | 33,9910 |
| CD63 antigen (Fragment | Cd63 | 18 | 0,0568 | 0,0138 | 24,3059 | 0,0678 | 0,0160 | 23,5822 |
| Cathepsin D | Ctsd | 44 | 0,0293 | 0,0078 | 26,7861 | 0,0164 | 0,0033 | 19,8798 |
| BUB3 mitotic checkpoint protein | Bub3 | 37 | 0,0253 | 0,0084 | 33,2386 | 0,0345 | 0,0102 | 29,6498 |
| Erbb2 interacting protein | Erbin | 145 | 0,0065 | 0,0011 | 16,5913 | 0,0085 | 0,0041 | 48,6864 |
| dolichyl-phosphate beta-glucosyltransferase | Alg5 | 40 | 0,0217 | 0,0115 | 53,2891 | 0,0335 | 0,0188 | 56,2847 |
| 80K protein | Marcks | 30 | 0,0372 | 0,0066 | 17,8644 | 0,0349 | 0,0236 | 67,8405 |
| Cluster of Guanine nucleotide-binding protein subunit | Gna11 | 42 | 0,0286 | 0,0148 | 51,5897 | 0,0185 | 0,0142 | 76,5298 |
| DNA replication licensing factor | Mcm7 | 81 | 0,0149 | 0,0079 | 52,8045 | 0,0106 | 0,0096 | 90,5910 |
| Gem nuclear organelle associated | Gemin5 | 168 | 0,0076 | 0,0031 | 40,6310 | 0,0040 | 0,0029 | 73,4444 |
| Cluster of H-2 class I histocompatibility antigen | H2-K1 | 41 | 0,0248 | 0,0087 | 35,1838 | 0,0263 | 0,0182 | 69,0426 |
| Eukaryotic translation initiation factor 3 subunit | Eif3e | 52 | 0,0262 | 0,0071 | 27,2525 | 0,0101 | 0,0072 | 71,5447 |
| Embigin | Emb | 37 | 0,0300 | 0,0086 | 28,5697 | 0,0287 | 0,0108 | 37,8164 |
| Galectin-1 | Lgals1 | 15 | 0,1024 | 0,0097 | 9,4467 | 0,0227 | 0,0268 | 118,4137 |
| Kras protein | Kras | 21 | 0,0610 | 0,0240 | 39,3382 | 0,0343 | 0,0068 | 19,8798 |
| RNA polymerase II-associated | Rpap3 | 74 | 0,0221 | 0,0051 | 23,3062 | 0,0015 | 0,0030 | 200,0000 |
| Serine/arginine-rich splicing | Srsf2 | 25 | 0,0650 | 0,0059 | 9,0958 | 0,0057 | 0,0114 | 200,0000 |
| Armet protein | Manf | 19 | 0,0905 | 0,0168 | 18,6131 | 0,0000 | 0,0000 |  |
| Adenylosuccinate lyase | Adsl | 53 | 0,0177 | 0,0029 | 16,5913 | 0,0230 | 0,0054 | 23,5822 |
| Actin-related protein 2/3 complex | Arpc4 | 20 | 0,0474 | 0,0103 | 21,6689 | 0,0610 | 0,0144 | 23,5822 |
| Golgi integral membrane protein | Golim4 | 80 | 0,0107 | 0,0027 | 25,4399 | 0,0162 | 0,0059 | 36,3730 |
| Thioesterase superfamily member 2 | Acot13 | 15 | 0,0398 | 0,0111 | 27,8362 | 0,1130 | 0,0262 | 23,1452 |
| Secretory carrier-associated membrane | Scamp2 | 36 | 0,0260 | 0,0086 | 33,2386 | 0,0319 | 0,0212 | 66,3488 |
| Albumin | Alb | 69 | 0,0113 | 0,0052 | 45,4958 | 0,0224 | 0,0132 | 58,8450 |
| Uncharacterized protein | Tbl2 | 50 | 0,0205 | 0,0054 | 26,4226 | 0,0198 | 0,0074 | 37,4650 |
| Syntaxin-7 | Stx7 | 30 | 0,0347 | 0,0176 | 50,7556 | 0,0364 | 0,0215 | 58,9967 |
| Epoxide hydrolase | Ephx1 | 53 | 0,0209 | 0,0022 | 10,5881 | 0,0166 | 0,0056 | 33,4900 |
| Cluster of Large ribosomal subunit protein | Rpl29 | 18 | 0,0565 | 0,0256 | 45,3226 | 0,0551 | 0,0206 | 37,4650 |
| Adenylate kinase 4, mitochondrial | Ak4 | 25 | 0,0379 | 0,0082 | 21,6689 | 0,0469 | 0,0159 | 33,9910 |
| Large ribosomal subunit protein | Mrpl45 | 35 | 0,0268 | 0,0124 | 46,3583 | 0,0299 | 0,0214 | 71,5447 |
| S5 DRBM domain-containing | Mrps5 | 48 | 0,0211 | 0,0089 | 42,1564 | 0,0196 | 0,0117 | 59,5449 |
| Coronin | Coro1b | 54 | 0,0206 | 0,0064 | 30,7713 | 0,0179 | 0,0101 | 56,6454 |
| Protein THEM6 | Them6 | 24 | 0,0464 | 0,0073 | 15,8082 | 0,0353 | 0,0292 | 82,6563 |
| Extended synaptotagmin-2 | Esyt2 | 94 | 0,0136 | 0,0042 | 30,8747 | 0,0056 | 0,0069 | 122,7714 |
| RAB31, member RAS oncogene | Rab31 | 21 | 0,0655 | 0,0147 | 22,4561 | 0,0189 | 0,0256 | 136,0264 |
| G-rich RNA sequence binding | Grsf1 | 42 | 0,0345 | 0,0025 | 7,1684 | 0,0073 | 0,0091 | 124,2177 |
| AP-3 complex subunit beta | Ap3b1 | 123 | 0,0111 | 0,0018 | 16,1832 | 0,0027 | 0,0054 | 200,0000 |
| Proteasome subunit beta type | Psmb4 | 29 | 0,0500 | 0,0036 | 7,1684 | 0,0076 | 0,0153 | 200,0000 |
| Probable ATP-dependent | Ddx6 | 54 | 0,0270 | 0,0041 | 15,1461 | 0,0041 | 0,0082 | 200,0000 |
| RNA helicase | Mov10 | 121 | 0,0126 | 0,0045 | 36,0292 | 0,0012 | 0,0024 | 200,0000 |
| SAP domain-containing protein | Sarnp | 24 | 0,0682 | 0,0164 | 24,0526 | 0,0000 | 0,0000 |  |
| Peptidyl-prolyl cis-trans isomerase | Ppif | 22 | 0,0740 | 0,0087 | 11,7351 | 0,0000 | 0,0000 |  |
| UBC core domain-containing | Ube2j1 | 35 | 0,0269 | 0,0096 | 35,5679 | 0,0278 | 0,0091 | 32,5529 |
| Protein FAM162A | Fam162a | 18 | 0,0381 | 0,0018 | 4,6620 | 0,0840 | 0,0401 | 47,8194 |
| Lclat1 protein | Lclat1 | 44 | 0,0194 | 0,0076 | 39,4768 | 0,0277 | 0,0065 | 23,5822 |
| 40S ribosomal protein S27 | Rps27 | 9 | 0,1133 | 0,0265 | 23,4088 | 0,0924 | 0,0383 | 41,4123 |
| Lectin, mannose-binding 2-like (Fragment | Lman2l | 23 | 0,0408 | 0,0068 | 16,5913 | 0,0461 | 0,0174 | 37,8164 |
| Endoplasmic reticulum-Golgi intermediate compartment | Ergic3 | 43 | 0,0198 | 0,0039 | 19,6046 | 0,0264 | 0,0057 | 21,4643 |
| Matrin-3 | Matr3 | 95 | 0,0108 | 0,0005 | 4,6620 | 0,0093 | 0,0031 | 33,4900 |
| NADH dehydrogenase [ubiquinone] flavoprotein | Ndufv1 | 50 | 0,0207 | 0,0064 | 30,9137 | 0,0173 | 0,0042 | 24,4685 |
| Fibrinogen alpha chain | Fga | 87 | 0,0080 | 0,0059 | 73,8761 | 0,0184 | 0,0130 | 71,0475 |
| Transmembrane 9 superfamily member | Tm9sf1 | 55 | 0,0157 | 0,0066 | 42,4231 | 0,0190 | 0,0136 | 71,5447 |
| DnaJ heat shock protein family (Hsp | Dnajc19 | 12 | 0,0854 | 0,0226 | 26,4226 | 0,0810 | 0,0834 | 102,9430 |
| Dolichol-phosphate mannosyltransferase subunit | Dpm1 | 29 | 0,0323 | 0,0107 | 33,2386 | 0,0340 | 0,0272 | 80,0565 |
| Proteasome subunit alpha type | Psma3 | 28 | 0,0370 | 0,0112 | 30,2881 | 0,0278 | 0,0212 | 76,5298 |
| AP-2 complex subunit | Ap2m1 | 50 | 0,0187 | 0,0057 | 30,7641 | 0,0194 | 0,0146 | 75,1943 |
| F-actin-capping protein subunit | Capzb | 29 | 0,0383 | 0,0177 | 46,2754 | 0,0230 | 0,0169 | 73,4444 |
| Proteasome (Prosome, macropain) 26S subunit, non | Psmd7 | 37 | 0,0298 | 0,0079 | 26,4746 | 0,0186 | 0,0148 | 79,3941 |
| Serine hydrolase-like | Serhl | 36 | 0,0258 | 0,0111 | 42,9183 | 0,0251 | 0,0216 | 86,1335 |
| U2 small nuclear ribonucleoprotein auxiliary | U2af1 | 28 | 0,0399 | 0,0075 | 18,7423 | 0,0257 | 0,0051 | 19,8798 |
| Protein O-glucosyltransferase 2 | Poglut2 | 15 | 0,0739 | 0,0078 | 10,5881 | 0,0455 | 0,0359 | 78,9119 |
| Sorting nexin-2 | Snx2 | 58 | 0,0193 | 0,0036 | 18,7423 | 0,0106 | 0,0124 | 116,1446 |
| Acidic leucine-rich nuclear phosphoprotein 32 family member (Fragment | Anp32b | 28 | 0,0427 | 0,0064 | 15,0531 | 0,0181 | 0,0212 | 117,2710 |
| Amyloid-beta precursor protein | App | 87 | 0,0126 | 0,0053 | 41,8185 | 0,0071 | 0,0082 | 116,1446 |
| NOP2 nucleolar protein | Nop2 | 87 | 0,0110 | 0,0061 | 56,0154 | 0,0100 | 0,0116 | 115,5066 |
| Glycine--tRNA ligase | Gars1 | 82 | 0,0123 | 0,0062 | 50,5013 | 0,0092 | 0,0127 | 139,1106 |
| Delta-6 desaturase (Fragment | Fads2 | 51 | 0,0181 | 0,0104 | 57,4051 | 0,0175 | 0,0185 | 105,4173 |
| GPI transamidase component PIG | Pigt | 65 | 0,0193 | 0,0135 | 69,7876 | 0,0061 | 0,0083 | 136,0264 |
| Platelet-activating factor acetylhydrolase IB subunit beta | Pafah1b1 | 47 | 0,0215 | 0,0090 | 41,6996 | 0,0129 | 0,0149 | 115,8293 |
| Cluster of TMEM9 domain family, member | Tmem9b | 14 | 0,0610 | 0,0131 | 21,5150 | 0,0731 | 0,0160 | 21,9446 |
| Protein YIPF5 | Yipf5 | 28 | 0,0273 | 0,0047 | 17,1468 | 0,0430 | 0,0186 | 43,3969 |
| Gap junction gamma-3 | Gjc3 | 30 | 0,0227 | 0,0092 | 40,2863 | 0,0460 | 0,0152 | 33,0667 |
| Cytochrome b5 heme-binding domain-containing protein | Cyb5b | 16 | 0,0427 | 0,0173 | 40,5231 | 0,0886 | 0,0408 | 46,0651 |
| ER membrane protein complex subunit | Emc2 | 35 | 0,0220 | 0,0049 | 22,4277 | 0,0326 | 0,0178 | 54,7700 |
| Sigma non-opioid intracellular | Sigmar1 | 22 | 0,0502 | 0,0133 | 26,4746 | 0,0277 | 0,0065 | 23,5822 |
| NADH dehydrogenase [ubiquinone] 1 beta subcomplex | Ndufb9 | 22 | 0,0391 | 0,0099 | 25,4399 | 0,0474 | 0,0356 | 75,1190 |
| Trimethyllysine dioxygenase, mitochondrial | Tmlhe | 50 | 0,0172 | 0,0042 | 24,7343 | 0,0187 | 0,0126 | 67,2051 |
| 2',3'-cyclic-nucleotide | Cnp | 47 | 0,0217 | 0,0101 | 46,3530 | 0,0135 | 0,0116 | 85,8524 |
| Annexin A5 | Anxa5 | 36 | 0,0290 | 0,0141 | 48,7269 | 0,0220 | 0,0196 | 89,0902 |
| Neurolysin (metallopeptidase M3 family | Nln | 78 | 0,0131 | 0,0030 | 22,9666 | 0,0081 | 0,0070 | 85,8524 |
| Aspartate--tRNA ligase, mitochondrial | Dars2 | 74 | 0,0126 | 0,0039 | 31,1913 | 0,0106 | 0,0100 | 94,3826 |
| ATPase family AAA domain-containing | Atad3 | 67 | 0,0139 | 0,0060 | 43,2046 | 0,0121 | 0,0106 | 87,5237 |
| dolichyl-diphosphooligosaccharide--protein glycotransferase | Stt3b | 93 | 0,0100 | 0,0062 | 61,5061 | 0,0082 | 0,0096 | 117,2710 |
| Saccharopine dehydrogenase NADP binding domain-containing | Sccpdh | 35 | 0,0290 | 0,0123 | 42,3707 | 0,0186 | 0,0235 | 126,5704 |
| Cluster of Insulin-like growth factor 2 mRNA-binding protein | Igf2bp3 | 64 | 0,0148 | 0,0053 | 35,8485 | 0,0119 | 0,0139 | 117,2710 |
| Prolyl 3-hydroxylase 2 | P3h2 | 80 | 0,0127 | 0,0091 | 71,8862 | 0,0073 | 0,0104 | 142,6799 |
| Proteasome subunit beta type | Psmb6 | 25 | 0,0447 | 0,0084 | 18,7423 | 0,0197 | 0,0254 | 128,9647 |
| WD repeat-containing protein | Wdr1 | 66 | 0,0180 | 0,0059 | 32,7570 | 0,0055 | 0,0067 | 120,7865 |
| Large ribosomal subunit protein | Mrpl11 | 21 | 0,0616 | 0,0181 | 29,3755 | 0,0129 | 0,0153 | 119,1500 |
| DNA replication licensing factor | Mcm6 | 93 | 0,0129 | 0,0019 | 15,0531 | 0,0039 | 0,0047 | 120,7865 |
| Myb-binding protein 1A | Mybbp1a | 152 | 0,0078 | 0,0040 | 50,7986 | 0,0028 | 0,0056 | 200,0000 |
| Translationally-controlled tumor protein | Tpt1 | 19 | 0,0675 | 0,0085 | 12,6634 | 0,0117 | 0,0233 | 200,0000 |
| Golgi phosphoprotein 3 | Golph3 | 27 | 0,0507 | 0,0107 | 21,0747 | 0,0041 | 0,0082 | 200,0000 |
| Large ribosomal subunit protein | Mrpl58 | 23 | 0,0634 | 0,0151 | 23,8048 | 0,0000 | 0,0000 |  |
| RNA-binding protein EWS | Ewsr1 | 69 | 0,0210 | 0,0044 | 20,7264 | 0,0000 | 0,0000 |  |
| A-kinase anchor protein | Akap1 | 92 | 0,0056 | 0,0023 | 40,5573 | 0,0162 | 0,0036 | 22,4077 |
| CD9 antigen | Cd9 | 25 | 0,0306 | 0,0126 | 41,1418 | 0,0416 | 0,0239 | 57,4398 |
| Vesicle-associated membrane protein | Vamp4 | 16 | 0,0375 | 0,0109 | 29,1608 | 0,0747 | 0,0445 | 59,5528 |
| 3'(2'), 5'-bisphosphate | Bpnt1 | 35 | 0,0245 | 0,0098 | 39,9794 | 0,0247 | 0,0060 | 24,4685 |
| Scavenger receptor class | Scarb1 | 57 | 0,0119 | 0,0044 | 36,9462 | 0,0214 | 0,0050 | 23,5822 |
| Elongation factor 1-beta | Eef1b | 25 | 0,0413 | 0,0123 | 29,7036 | 0,0244 | 0,0058 | 23,5822 |
| Phosphatidylserine lipase ABHD16A | Abhd16a | 63 | 0,0137 | 0,0060 | 43,4972 | 0,0142 | 0,0089 | 62,7759 |
| NADH dehydrogenase [ubiquinone] 1 beta subcomplex | Ndufb7 | 16 | 0,0532 | 0,0104 | 19,6046 | 0,0574 | 0,0288 | 50,1246 |
| Asparagine--tRNA ligase, cytoplasmic | NARS1 | 64 | 0,0120 | 0,0021 | 17,1468 | 0,0155 | 0,0058 | 37,4650 |
| Small ribosomal subunit protein | Mrps7 | 28 | 0,0302 | 0,0146 | 48,3480 | 0,0295 | 0,0205 | 69,4697 |
| Protein sprouty homolog 4 | Spry4 | 33 | 0,0236 | 0,0105 | 44,4811 | 0,0316 | 0,0238 | 75,1190 |
| Antigen peptide transporter 2 | Tap2 | 77 | 0,0101 | 0,0026 | 25,5979 | 0,0115 | 0,0098 | 84,7412 |
| Importin subunit alpha-1 | Kpna2 | 58 | 0,0162 | 0,0058 | 35,5679 | 0,0115 | 0,0084 | 73,4444 |
| Retinoblastoma binding protein 7, chromatin remodeling | Rbbp7 | 44 | 0,0212 | 0,0063 | 29,8294 | 0,0164 | 0,0033 | 19,8798 |
| Desmoglein 1 beta | Dsg1b | 114 | 0,0045 | 0,0030 | 66,9551 | 0,0116 | 0,0087 | 75,0053 |
| Methionine--tRNA ligase, cytoplasmic | Mars1 | 102 | 0,0100 | 0,0046 | 46,0525 | 0,0047 | 0,0062 | 132,6393 |
| Methionine aminopeptidase 2 | Metap2 | 53 | 0,0209 | 0,0022 | 10,5881 | 0,0072 | 0,0085 | 118,5050 |
| La ribonucleoprotein 4 | Larp4 | 80 | 0,0149 | 0,0055 | 37,0290 | 0,0028 | 0,0055 | 200,0000 |
| Prostaglandin E synthase 3 | Ptges3 | 15 | 0,0850 | 0,0262 | 30,8747 | 0,0106 | 0,0213 | 200,0000 |
| NADH dehydrogenase [ubiquinone] 1 alpha subcomplex | Ndufa8 | 20 | 0,0646 | 0,0313 | 48,4562 | 0,0055 | 0,0111 | 200,0000 |
| Myeloid-derived growth factor | Mydgf | 18 | 0,0763 | 0,0175 | 22,9094 | 0,0000 | 0,0000 |  |
| Poly(U)-binding-splicing | Puf60 | 60 | 0,0224 | 0,0138 | 61,5284 | 0,0000 | 0,0000 |  |
| Torsin-1A-interacting protein | Tor1aip2 | 54 | 0,0110 | 0,0030 | 27,1745 | 0,0226 | 0,0053 | 23,5822 |
| Neurotrimin | Ntm | 35 | 0,0148 | 0,0102 | 69,0966 | 0,0405 | 0,0187 | 46,0651 |
| Anti-dectin-1 15E2 |  | 26 | 0,0234 | 0,0136 | 58,0964 | 0,0496 | 0,0350 | 70,4732 |
| Sphingomyelin phosphodiesterase 2 | Smpd2 | 47 | 0,0183 | 0,0045 | 24,7343 | 0,0153 | 0,0030 | 19,8798 |
| DNA replication licensing factor | Mcm5 | 82 | 0,0104 | 0,0054 | 52,2329 | 0,0088 | 0,0017 | 19,8798 |
| Pituitary tumor-transforming 1 interacting protein (Fragment | Pttg1ip | 13 | 0,0264 | 0,0012 | 4,6620 | 0,1364 | 0,0784 | 57,4437 |
| Small ribosomal subunit protein | Mrps27 | 48 | 0,0141 | 0,0052 | 36,6643 | 0,0223 | 0,0131 | 58,7329 |
| Syntaxin 5A (Fragment | Stx5a | 29 | 0,0295 | 0,0157 | 53,0995 | 0,0260 | 0,0102 | 39,1752 |
| Acetyl-coenzyme A synthetase | Acss1 | 75 | 0,0102 | 0,0017 | 17,1468 | 0,0115 | 0,0028 | 24,4685 |
| Small ribosomal subunit protein | Rps10 | 19 | 0,0359 | 0,0145 | 40,2863 | 0,0541 | 0,0264 | 48,7495 |
| Sorting nexin | Snx5 | 47 | 0,0145 | 0,0059 | 40,5231 | 0,0211 | 0,0079 | 37,4650 |
| Cluster of Metaxin | Mtx1 | 34 | 0,0200 | 0,0081 | 40,2863 | 0,0229 | 0,0175 | 76,5298 |
| Uncharacterized |  | 62 | 0,0140 | 0,0076 | 54,0834 | 0,0111 | 0,0088 | 79,3941 |
| V-type proton ATPase subunit d 1 OS=Mus | Atp6v0d1 | 40 | 0,0193 | 0,0043 | 22,4277 | 0,0216 | 0,0053 | 24,4685 |
| Pyridoxal-dependent decarboxylase domain | Pdxdc1 | 87 | 0,0078 | 0,0029 | 36,9462 | 0,0122 | 0,0046 | 37,8164 |
| Basic leucine zipper and | Bzw1 | 51 | 0,0202 | 0,0099 | 48,8087 | 0,0081 | 0,0056 | 69,4697 |
| Large ribosomal subunit protein | Mrpl28 | 30 | 0,0258 | 0,0064 | 24,6396 | 0,0288 | 0,0070 | 24,4685 |
| Large ribosomal subunit protein | Mrpl38 | 45 | 0,0189 | 0,0037 | 19,6046 | 0,0141 | 0,0121 | 85,8524 |
| 26S proteasome non-ATPase regulatory | Psmd6 | 46 | 0,0164 | 0,0103 | 62,7880 | 0,0169 | 0,0129 | 76,5298 |
| Sphingolipid delta(4)-desaturase | Degs1 | 38 | 0,0222 | 0,0106 | 47,6530 | 0,0175 | 0,0129 | 73,4444 |
| Cluster of 2'-5'-oligoadenylate synthase | Oas1a | 42 | 0,0203 | 0,0045 | 22,3465 | 0,0155 | 0,0179 | 115,5201 |
| ADP-ribosylation factor-like 6 interacting protein 1 (Fragment | Arl6ip1 | 16 | 0,0637 | 0,0146 | 22,9666 | 0,0258 | 0,0179 | 69,4697 |
| Very-long-chain (3R)-3-hydroxyacyl-Co | Hacd3 | 43 | 0,0220 | 0,0081 | 36,9333 | 0,0126 | 0,0150 | 119,1500 |
| Catalase | Cat | 60 | 0,0141 | 0,0068 | 48,3480 | 0,0100 | 0,0139 | 138,7323 |
| L-lactate dehydrogenase | Ldha | 35 | 0,0320 | 0,0131 | 40,8706 | 0,0072 | 0,0085 | 117,2710 |
| RNA binding motif protein, X-linked | Rbmxl1 | 42 | 0,0266 | 0,0050 | 18,7423 | 0,0060 | 0,0071 | 117,2710 |
| ATP synthase F1 subunit | Atp5f1d | 18 | 0,0522 | 0,0087 | 16,5913 | 0,0291 | 0,0208 | 71,5447 |
| GDP-fucose protein | Pofut1 | 40 | 0,0256 | 0,0069 | 26,7939 | 0,0103 | 0,0072 | 69,4697 |
| CCA tRNA nucleotidyltransferase 1 | Trnt1 | 50 | 0,0223 | 0,0035 | 15,8082 | 0,0051 | 0,0059 | 117,2710 |
| Stromal interaction molecule 1 | Stim1 | 78 | 0,0121 | 0,0023 | 18,9950 | 0,0057 | 0,0114 | 200,0000 |
| Microtubule-associated protein 2 | Map2 | 199 | 0,0055 | 0,0019 | 33,6302 | 0,0016 | 0,0032 | 200,0000 |
| Lupus La protein homolog | Ssb | 48 | 0,0251 | 0,0051 | 20,3912 | 0,0023 | 0,0046 | 200,0000 |
| Cluster of Interferon-induced protein with tetratricopeptide | Ifit1 | 54 | 0,0222 | 0,0083 | 37,4142 | 0,0026 | 0,0053 | 200,0000 |
| U2 small nuclear ribonucleoprotein | Snrpb2 | 25 | 0,0414 | 0,0125 | 30,2881 | 0,0089 | 0,0177 | 200,0000 |
| Peptidyl-prolyl cis-trans | Fkbp7 | 25 | 0,0515 | 0,0138 | 26,7861 | 0,0000 | 0,0000 |  |
| Transformer 2 alpha | Tra2a | 32 | 0,0402 | 0,0109 | 27,1847 | 0,0000 | 0,0000 |  |
| Superoxide dismutase [Cu-Zn | Sod1 | 16 | 0,0813 | 0,0393 | 48,2837 | 0,0000 | 0,0000 |  |
| Polymerase (DNA-directed), delta interacting protein | Poldip2 | 33 | 0,0384 | 0,0203 | 52,9611 | 0,0000 | 0,0000 |  |
| Far upstream element (FUSE) binding | Fubp3 | 61 | 0,0211 | 0,0074 | 34,9124 | 0,0000 | 0,0000 |  |
| Heterogeneous nuclear ribonucleoprotein D | Hnrnpdl | 46 | 0,0247 | 0,0167 | 67,7690 | 0,0000 | 0,0000 |  |
| Signal peptidase complex catalytic subunit | Sec11a | 28 | 0,0272 | 0,0109 | 39,9016 | 0,0269 | 0,0105 | 39,1752 |
| Type 2 phosphatidylinositol 4,5-bisphosphate 4-phosphatase | Pip4p2 | 28 | 0,0276 | 0,0068 | 24,6396 | 0,0275 | 0,0131 | 47,6065 |
| Oxygen-dependent coproporphyrinogen-III oxidase | Cpox | 50 | 0,0137 | 0,0006 | 4,6620 | 0,0173 | 0,0042 | 24,4685 |
| Vesicle transport protein GOT1B | Golt1b | 15 | 0,0401 | 0,0228 | 56,8332 | 0,0751 | 0,0487 | 64,8236 |
| NADH dehydrogenase [ubiquinone] 1 alpha subcomplex | Ndufa13 | 17 | 0,0405 | 0,0172 | 42,6186 | 0,0508 | 0,0124 | 24,4685 |
| Glutaryl-Coenzyme A dehydrogenase | Gcdh | 50 | 0,0121 | 0,0038 | 31,4263 | 0,0211 | 0,0111 | 52,6266 |
| Torsin-3A | Tor3a | 44 | 0,0156 | 0,0007 | 4,6620 | 0,0189 | 0,0078 | 41,4123 |
| Signal peptidase complex subunit | Spcs3 | 20 | 0,0383 | 0,0066 | 17,1468 | 0,0333 | 0,0245 | 73,4444 |
| Endophilin-B1 | Sh3glb1 | 44 | 0,0157 | 0,0066 | 41,7876 | 0,0200 | 0,0225 | 112,6555 |
| Perilipin-3 | Plin3 | 47 | 0,0183 | 0,0047 | 25,4399 | 0,0111 | 0,0080 | 71,5447 |
| 28S ribosomal protein S2 | Mrps2 | 32 | 0,0291 | 0,0087 | 29,8294 | 0,0141 | 0,0104 | 73,8033 |
| Haloacid dehalogenase-like hydrolase domain-containing | Hdhd3 | 28 | 0,0273 | 0,0047 | 17,1468 | 0,0221 | 0,0256 | 116,1446 |
| Constitutive coactivator of PPAR-gamma | FAM120A | 122 | 0,0063 | 0,0011 | 17,1468 | 0,0052 | 0,0045 | 85,8524 |
| Prolactin regulatory element-binding | Preb | 38 | 0,0156 | 0,0084 | 53,5801 | 0,0242 | 0,0199 | 82,3271 |
| Large ribosomal subunit protein | Mrpl17 | 20 | 0,0385 | 0,0162 | 42,0554 | 0,0317 | 0,0272 | 85,8524 |
| Sulfate transporter | Slc26a2 | 82 | 0,0103 | 0,0050 | 48,7128 | 0,0068 | 0,0058 | 85,1082 |
| ADP-ribosylation factor-like | Arl1 | 16 | 0,0540 | 0,0281 | 52,1208 | 0,0317 | 0,0371 | 117,2710 |
| Fragile X messenger ribonucleoprotein | Fmr1 | 71 | 0,0109 | 0,0028 | 25,5979 | 0,0056 | 0,0076 | 136,0264 |
| Cluster of C-terminal binding | Ctbp1 | 47 | 0,0202 | 0,0078 | 38,5528 | 0,0077 | 0,0094 | 120,7865 |
| MKIAA0051 protein (Fragment | Iqgap1 | 191 | 0,0049 | 0,0008 | 16,5913 | 0,0020 | 0,0024 | 118,5050 |
| Armadillo repeat-containing protein | Armc1 | 31 | 0,0251 | 0,0113 | 45,2539 | 0,0243 | 0,0095 | 39,1752 |
| Lipid droplet-regulating VLDL assembly | Aup1 | 32 | 0,0294 | 0,0051 | 17,4028 | 0,0114 | 0,0137 | 120,7865 |
| RNA helicase | Ddx21 | 94 | 0,0100 | 0,0037 | 36,5893 | 0,0048 | 0,0035 | 73,8033 |
| Proteasome subunit alpha type | Psma2 | 26 | 0,0426 | 0,0045 | 10,5881 | 0,0043 | 0,0085 | 200,0000 |
| V-type proton ATPase subunit E 1 OS=Mus | Atp6v1e1 | 26 | 0,0361 | 0,0167 | 46,3583 | 0,0128 | 0,0256 | 200,0000 |
| Cluster of Septin OS | Septin7 | 51 | 0,0200 | 0,0047 | 23,4088 | 0,0050 | 0,0058 | 117,2710 |
| Chromobox protein homolog 3 | Cbx3 | 21 | 0,0570 | 0,0086 | 15,0531 | 0,0000 | 0,0000 |  |
| 40S ribosomal |  | 9 | 0,1338 | 0,0414 | 30,9791 | 0,0000 | 0,0000 |  |
| Proteasome subunit beta type | Psmb5 | 29 | 0,0409 | 0,0137 | 33,4195 | 0,0000 | 0,0000 |  |
| Transcriptional activator protein Pur | Purb | 34 | 0,0354 | 0,0110 | 30,9791 | 0,0000 | 0,0000 |  |
| Coatomer subunit epsilon | Cope | 35 | 0,0340 | 0,0119 | 35,1224 | 0,0000 | 0,0000 |  |
| Cholinesterase | Bche | 68 | 0,0162 | 0,0128 | 78,7628 | 0,0021 | 0,0042 | 200,0000 |
| Ndufa4, mitochondrial complex associated | Ndufa4 | 6 | 0,0720 | 0,0313 | 43,4972 | 0,1970 | 0,0849 | 43,0874 |
| Acid sphingomyelinase-like phosphodiesterase | Smpdl3b | 52 | 0,0113 | 0,0056 | 49,8464 | 0,0172 | 0,0108 | 62,7759 |
| O-acyltransferase | Soat1 | 64 | 0,0106 | 0,0061 | 57,0313 | 0,0113 | 0,0022 | 19,8798 |
| RIKEN cDNA D630045J12 gene | D630045J12Rik | 194 | 0,0035 | 0,0002 | 4,6620 | 0,0034 | 0,0025 | 73,4444 |
| NHP2-like protein 1 | Snu13 | 14 | 0,0553 | 0,0142 | 25,5979 | 0,0374 | 0,0268 | 71,5447 |
| Protein NipSnap homolog 2 | Nipsnap2 | 33 | 0,0208 | 0,0010 | 4,6620 | 0,0202 | 0,0148 | 73,4444 |
| Small ribosomal subunit protein | Mrps16 | 15 | 0,0398 | 0,0111 | 27,8362 | 0,0587 | 0,0197 | 33,4900 |
| Large ribosomal subunit protein | Mrpl3 | 34 | 0,0223 | 0,0114 | 50,9529 | 0,0154 | 0,0110 | 71,5447 |
| Membrane insertase YidC/Oxa/ALB C-terminal domain-containing protein | Oxa1l | 48 | 0,0126 | 0,0040 | 31,4263 | 0,0173 | 0,0072 | 41,4123 |
| Cysteine and glycine-rich | Csrp1 | 21 | 0,0407 | 0,0091 | 22,3465 | 0,0215 | 0,0158 | 73,8033 |
| Transaldolase | Taldo1 | 42 | 0,0207 | 0,0087 | 42,0961 | 0,0091 | 0,0108 | 118,5050 |
| Transmembrane protein 126A | Tmem126a | 22 | 0,0311 | 0,0015 | 4,6620 | 0,0303 | 0,0222 | 73,4444 |
| Tripeptidyl-peptidase 1 | Tpp1 | 61 | 0,0099 | 0,0031 | 31,4263 | 0,0133 | 0,0116 | 87,5237 |
| Solute carrier family 2, facilitated glucose transporter member | Slc2a3 | 53 | 0,0131 | 0,0058 | 44,0380 | 0,0142 | 0,0056 | 39,1752 |
| Collagen alpha-1(II | Col2a1 | 142 | 0,0060 | 0,0012 | 19,6046 | 0,0029 | 0,0020 | 69,4697 |
| NAD(P)-dependent steroid dehydrogenase-like | Nsdhl | 41 | 0,0211 | 0,0092 | 43,4972 | 0,0089 | 0,0107 | 120,7865 |
| GPI anchor attachment protein | Gpaa1 | 61 | 0,0126 | 0,0028 | 22,4277 | 0,0088 | 0,0133 | 150,2277 |
| Heat shock 70 kDa | Hspa13 | 52 | 0,0148 | 0,0061 | 41,5070 | 0,0095 | 0,0122 | 128,9647 |
| Caseinolytic mitochondrial matrix peptidase chaperone | Clpx | 67 | 0,0128 | 0,0029 | 22,3465 | 0,0067 | 0,0050 | 73,8033 |
| ATP binding cassette subfamily G member 2 (Junior blood | Abcg2 | 73 | 0,0094 | 0,0004 | 4,6620 | 0,0094 | 0,0075 | 79,3941 |
| Receptor expression-enhancing protein | Reep6 | 22 | 0,0427 | 0,0074 | 17,4028 | 0,0154 | 0,0183 | 118,4137 |
| Nucleolar protein 58 | Nop58 | 60 | 0,0157 | 0,0030 | 18,9950 | 0,0050 | 0,0058 | 115,8293 |
| 28S ribosomal protein S9 | Mrps9 | 46 | 0,0204 | 0,0034 | 16,5913 | 0,0055 | 0,0065 | 117,2710 |
| Fascin | Fscn1 | 55 | 0,0186 | 0,0045 | 24,3059 | 0,0020 | 0,0040 | 200,0000 |
| LYR motif-containing protein | Lyrm4 | 11 | 0,0931 | 0,0246 | 26,4226 | 0,0101 | 0,0201 | 200,0000 |
| RNA helicase (Fragment | Rigi | 106 | 0,0063 | 0,0059 | 94,4328 | 0,0060 | 0,0051 | 85,8524 |
| Cluster of Long-chain-fatty-acid--Co | Acsl5 | 76 | 0,0135 | 0,0006 | 4,6620 | 0,0019 | 0,0038 | 200,0000 |
| ATP-dependent (S)-NAD(P)H-hydrate | Naxd | 35 | 0,0317 | 0,0034 | 10,5881 | 0,0000 | 0,0000 |  |
| Small ribosomal subunit protein | Mrps23 | 20 | 0,0564 | 0,0277 | 49,1656 | 0,0000 | 0,0000 |  |
| Glyoxalase domain-containing protein | Glod4 | 33 | 0,0341 | 0,0141 | 41,4117 | 0,0000 | 0,0000 |  |
| RRM domain-containing protein | Eif4b | 69 | 0,0162 | 0,0052 | 32,2654 | 0,0000 | 0,0000 |  |
| Carnitine deficiency-associated gene expressed in | Cdv3 | 24 | 0,0465 | 0,0083 | 17,8644 | 0,0000 | 0,0000 |  |
| Cleavage and polyadenylation specificity factor | Nudt21 | 26 | 0,0429 | 0,0132 | 30,7713 | 0,0000 | 0,0000 |  |
| 14-3-3 protein | Ywhae | 29 | 0,0267 | 0,0068 | 25,5979 | 0,0055 | 0,0110 | 200,0000 |
| Prelamin-A/C | Lmna | 74 | 0,0139 | 0,0093 | 66,9551 | 0,0027 | 0,0053 | 200,0000 |
| L-lactate dehydrogenase (Fragment | Ldhb | 21 | 0,0288 | 0,0091 | 31,4263 | 0,0358 | 0,0140 | 39,1752 |
| Protein YIPF3 | Yipf3 | 38 | 0,0137 | 0,0057 | 41,9291 | 0,0242 | 0,0121 | 50,1246 |
| Uncharacterized protein | Slc38a2 | 15 | 0,0335 | 0,0204 | 60,8011 | 0,0608 | 0,0249 | 40,9946 |
| Translocon-associated protein subunit | Ssr1 | 36 | 0,0120 | 0,0051 | 42,4231 | 0,0299 | 0,0122 | 40,9082 |
| 7-dehydrocholesterol reductase | Dhcr7 | 54 | 0,0111 | 0,0032 | 29,1608 | 0,0133 | 0,0027 | 19,8798 |
| Lysosome membrane protein 2 | Scarb2 | 54 | 0,0110 | 0,0030 | 27,1745 | 0,0133 | 0,0027 | 19,8798 |
| Large ribosomal subunit protein | Mrpl21 | 23 | 0,0184 | 0,0063 | 34,4368 | 0,0454 | 0,0341 | 75,1190 |
| TraB domain containing (Fragment | Trabd | 26 | 0,0130 | 0,0101 | 77,7930 | 0,0469 | 0,0111 | 23,5822 |
| Acylglycerol kinase, mitochondrial | Agk | 47 | 0,0109 | 0,0040 | 36,9002 | 0,0176 | 0,0122 | 69,4697 |
| 26S proteasome non-ATPase regulatory | Psmd14 | 35 | 0,0173 | 0,0054 | 31,4263 | 0,0190 | 0,0140 | 73,4444 |
| Splicing factor 3a, subunit | Sf3a3 | 59 | 0,0116 | 0,0047 | 40,2863 | 0,0083 | 0,0108 | 128,9647 |
| Transmembrane emp24 protein transport domain | Tmed5 | 26 | 0,0227 | 0,0115 | 50,4960 | 0,0256 | 0,0188 | 73,4444 |
| Alpha/beta hydrolase domain-containing protein | Abhd17b | 32 | 0,0214 | 0,0010 | 4,6620 | 0,0158 | 0,0186 | 117,2710 |
| Enoyl-CoA hydratase domain-containing protein | Echdc2 | 32 | 0,0215 | 0,0093 | 43,3459 | 0,0179 | 0,0165 | 92,3935 |
| Hydroxymethylglutaryl-CoA lyase, mitochondrial | Hmgcl | 34 | 0,0202 | 0,0009 | 4,6620 | 0,0179 | 0,0042 | 23,5822 |
| Mitochondrial pyruvate carrier 1 | Mpc1 | 12 | 0,0491 | 0,0421 | 85,5966 | 0,0601 | 0,0119 | 19,8798 |
| Cluster of Kinesin-1 heavy chain | Kif5b | 110 | 0,0070 | 0,0017 | 24,6396 | 0,0038 | 0,0044 | 115,8630 |
| Cluster of Envelope polyprotein |  | 40 | 0,0171 | 0,0008 | 4,6620 | 0,0135 | 0,0161 | 119,1500 |
| Derlin | Derl1 | 29 | 0,0236 | 0,0011 | 4,6620 | 0,0205 | 0,0168 | 82,1495 |
| Vacuole membrane protein 1 | Vmp1 | 8 | 0,0866 | 0,0376 | 43,3400 | 0,0763 | 0,0180 | 23,5822 |
| Large ribosomal subunit protein | Mrpl27 | 16 | 0,0418 | 0,0273 | 65,4460 | 0,0297 | 0,0394 | 132,6393 |
| Ras GTPase-activating protein | Rasa3 | 96 | 0,0089 | 0,0019 | 21,5150 | 0,0028 | 0,0034 | 119,1500 |
| P21-activated protein kinase-interacting protein 1 (Fragment | Pak1ip1 | 41 | 0,0189 | 0,0046 | 24,6396 | 0,0101 | 0,0070 | 69,4697 |
| KH domain-containing, RNA-binding, signal transduction-associated | Khdrbs1 | 48 | 0,0105 | 0,0088 | 83,6400 | 0,0173 | 0,0072 | 41,4123 |
| Delta(14)-sterol reductase | Lbr | 71 | 0,0107 | 0,0043 | 40,0364 | 0,0054 | 0,0064 | 118,5050 |
| 3-beta-hydroxysteroid-Delta(8),Delta | Ebp | 26 | 0,0194 | 0,0167 | 86,2288 | 0,0305 | 0,0243 | 79,6432 |
| Acyl-CoA thioesterase 2 | Acot2 | 50 | 0,0172 | 0,0044 | 25,4399 | 0,0051 | 0,0059 | 117,2710 |
| RAB3 GTPase activating protein subunit | Rab3gap2 | 153 | 0,0056 | 0,0029 | 52,1818 | 0,0017 | 0,0019 | 117,2710 |
| Basement membrane-specific heparan sulfate proteoglycan core | Hspg2 | 398 | 0,0024 | 0,0008 | 35,5679 | 0,0004 | 0,0007 | 200,0000 |
| DNA replication licensing factor | Mcm2 | 102 | 0,0057 | 0,0048 | 84,5011 | 0,0062 | 0,0053 | 85,8524 |
| Striatin | Strn | 86 | 0,0049 | 0,0037 | 76,4632 | 0,0117 | 0,0056 | 47,8143 |
| Dihydroorotate dehydrogenase (quinone), mitochondrial | Dhodh | 43 | 0,0219 | 0,0042 | 18,9950 | 0,0026 | 0,0052 | 200,0000 |
| PPIase cyclophilin-type domain-containing | Ppid | 41 | 0,0208 | 0,0109 | 52,2329 | 0,0075 | 0,0093 | 124,2177 |
| Ubiquitin carboxyl-terminal hydrolase | Usp14 | 52 | 0,0181 | 0,0034 | 18,9950 | 0,0021 | 0,0043 | 200,0000 |
| Cluster of Phenylalanine--tRNA ligase beta subunit | Farsb | 66 | 0,0127 | 0,0085 | 66,8862 | 0,0038 | 0,0045 | 117,2710 |
| Solute carrier family 25 (Mitochondrial carnitine/acylcarnitine translocase), member | Slc25a20 | 33 | 0,0208 | 0,0147 | 70,6595 | 0,0149 | 0,0192 | 128,9647 |
| Nucleosome assembly protein 1-like | Nap1l1 | 45 | 0,0209 | 0,0040 | 18,9950 | 0,0025 | 0,0049 | 200,0000 |
| Mitogen-activated protein kinase | Mapk1 | 41 | 0,0228 | 0,0070 | 30,7641 | 0,0035 | 0,0070 | 200,0000 |
| Peptidyl-prolyl cis-trans isomerase | Pin4 | 14 | 0,0733 | 0,0205 | 27,9909 | 0,0000 | 0,0000 | #DIV/0! |
| Parkinson disease protein 7 | Park7 | 20 | 0,0515 | 0,0149 | 29,0216 | 0,0000 | 0,0000 | #DIV/0! |
| Delta(3,5)-Delta(2,4)-dienoyl-CoA isomerase | Ech1 | 36 | 0,0286 | 0,0083 | 29,0396 | 0,0000 | 0,0000 | #DIV/0! |
| Cluster of PDZ domain-containing protein | Htra2 | 48 | 0,0215 | 0,0086 | 39,8818 | 0,0000 | 0,0000 | #DIV/0! |
| inorganic diphosphatase | Ppa2 | 38 | 0,0268 | 0,0062 | 22,9666 | 0,0000 | 0,0000 | #DIV/0! |
| Collagen, type IX, alpha | Col9a1 | 92 | 0,0111 | 0,0053 | 47,7016 | 0,0000 | 0,0000 | #DIV/0! |
| Prdx2 protein | Prdx2 | 22 | 0,0275 | 0,0086 | 31,4263 | 0,0277 | 0,0065 | 23,5822 |
| ER membrane protein complex | Emc8 | 23 | 0,0226 | 0,0095 | 41,9291 | 0,0313 | 0,0062 | 19,8798 |
| Translocon-associated protein subunit | Ssr3 | 21 | 0,0204 | 0,0082 | 39,9794 | 0,0437 | 0,0219 | 50,1246 |
| Rab3 GTPase-activating protein catalytic subunit | Rab3gap1 | 111 | 0,0053 | 0,0027 | 50,4960 | 0,0047 | 0,0034 | 71,5447 |
| Mitochondrial carrier homolog 1 | Mtch1 | 42 | 0,0163 | 0,0068 | 41,9640 | 0,0098 | 0,0068 | 69,4697 |
| CD276 antigen | Cd276 | 34 | 0,0176 | 0,0049 | 27,8362 | 0,0154 | 0,0110 | 71,5447 |
| Vesicle transport through interaction with t-SNAREs 1B (Fragment | Vti1b | 21 | 0,0284 | 0,0077 | 27,1745 | 0,0249 | 0,0178 | 71,5447 |
| F-actin-capping protein subunit | Capza2 | 33 | 0,0206 | 0,0077 | 37,5128 | 0,0137 | 0,0101 | 73,8033 |
| Adaptor protein complex AP-1, sigma 1 (Fragment | Ap1s1 | 22 | 0,0311 | 0,0015 | 4,6620 | 0,0188 | 0,0130 | 69,4697 |
| Mesoderm specific transcript | Mest | 40 | 0,0129 | 0,0051 | 39,8818 | 0,0192 | 0,0092 | 47,6065 |
| Integrin alpha-8 | Itga8 | 118 | 0,0050 | 0,0027 | 53,5801 | 0,0053 | 0,0046 | 86,0848 |
| Mitochondrial chaperone BCS1 | Bcs1l | 47 | 0,0107 | 0,0065 | 60,8011 | 0,0153 | 0,0030 | 19,8798 |
| Epidermal growth factor receptor kinase | Eps8 | 92 | 0,0065 | 0,0036 | 55,8350 | 0,0066 | 0,0016 | 23,5822 |
| Aldehyde dehydrogenase | Aldh3a2 | 59 | 0,0101 | 0,0028 | 27,8362 | 0,0089 | 0,0063 | 71,5447 |
| Reticulophagy regulator 1 | Retreg1 | 53 | 0,0115 | 0,0067 | 58,0964 | 0,0105 | 0,0089 | 85,1082 |
| Uncharacterized protein | Dkc1 | 57 | 0,0105 | 0,0060 | 56,8332 | 0,0107 | 0,0025 | 23,5822 |
| Cytochrome c oxidase assembly protein | Cox15 | 46 | 0,0110 | 0,0039 | 35,1838 | 0,0153 | 0,0107 | 69,9176 |
| Acyl-CoA (8-3 | Fads1 | 52 | 0,0097 | 0,0059 | 60,8011 | 0,0139 | 0,0028 | 19,8798 |
| Fibrinogen C-terminal domain-containing | Fgb | 55 | 0,0079 | 0,0060 | 76,0175 | 0,0178 | 0,0147 | 82,5327 |
| MIT domain-containing protein | Spart | 73 | 0,0092 | 0,0082 | 88,6912 | 0,0062 | 0,0046 | 73,8033 |
| Adaptor-related protein complex 2, sigma 1 subunit | Ap2s1 | 7 | 0,0617 | 0,0268 | 43,4972 | 0,1180 | 0,0819 | 69,4697 |
| Acyl-CoA thioesterase 7 | Acot7 | 43 | 0,0160 | 0,0068 | 42,6186 | 0,0085 | 0,0102 | 120,7865 |
| Proteasome (prosome, macropain) 26S subunit, non | Psmd12 | 51 | 0,0133 | 0,0049 | 36,9462 | 0,0071 | 0,0086 | 120,7865 |
| von Willebrand factor A domain-containing | Vwa8 | 213 | 0,0032 | 0,0001 | 4,6620 | 0,0017 | 0,0021 | 120,7865 |
| DZF domain-containing protein | Ilf2 | 43 | 0,0137 | 0,0068 | 49,8464 | 0,0122 | 0,0087 | 71,5447 |
| Syntaxin-binding protein 3 | Stxbp3 | 68 | 0,0113 | 0,0019 | 17,1468 | 0,0044 | 0,0051 | 115,8293 |
| RAB34, member RAS oncogene | Rab34 | 29 | 0,0176 | 0,0065 | 36,9002 | 0,0265 | 0,0126 | 47,6065 |
| AP-1 complex subunit mu | Ap1m1 | 49 | 0,0157 | 0,0068 | 43,3954 | 0,0052 | 0,0061 | 117,2710 |
| 24-hydroxycholesterol 7-alpha | Cyp39a1 | 54 | 0,0142 | 0,0058 | 41,1418 | 0,0056 | 0,0065 | 115,8293 |
| Nuclear pore complex protein | Nup155 | 150 | 0,0034 | 0,0030 | 87,0951 | 0,0043 | 0,0055 | 126,5704 |
| NADPH:adrenodoxin oxidoreductase, mitochondrial | Fdxr | 57 | 0,0121 | 0,0088 | 72,5986 | 0,0073 | 0,0085 | 115,8630 |
| Serine palmitoyltransferase 1 | Sptlc1 | 53 | 0,0127 | 0,0083 | 65,6220 | 0,0078 | 0,0054 | 69,4697 |
| Cluster of Collagen alpha-1(IV) chain | Col4a1 | 161 | 0,0022 | 0,0025 | 115,5199 | 0,0073 | 0,0066 | 89,9869 |
| 26S proteasome non-ATPase regulatory | Psmd8 | 35 | 0,0172 | 0,0095 | 55,0822 | 0,0164 | 0,0151 | 92,3935 |
| Striatin, calmodulin binding protein | Strn3 | 78 | 0,0055 | 0,0043 | 78,3979 | 0,0083 | 0,0105 | 126,5704 |
| Small ribosomal subunit protein | Ptcd3 | 78 | 0,0098 | 0,0041 | 41,5070 | 0,0028 | 0,0057 | 200,0000 |
| YTH domain-containing family | Ythdc1 | 62 | 0,0138 | 0,0030 | 21,5150 | 0,0018 | 0,0036 | 200,0000 |
| NAC-A/B domain-containing | Naca | 23 | 0,0412 | 0,0089 | 21,6689 | 0,0000 | 0,0000 |  |
| Peroxiredoxin-5 | Prdx5 | 23 | 0,0412 | 0,0089 | 21,6689 | 0,0000 | 0,0000 |  |
| Eukaryotic translation initiation factor 1A, X-chromosomal | Eif1ax | 16 | 0,0583 | 0,0179 | 30,7641 | 0,0000 | 0,0000 |  |
| Splicing factor 1 | Sf1 | 62 | 0,0151 | 0,0046 | 30,7641 | 0,0000 | 0,0000 |  |
| SPRY domain-containing protein | Spryd4 | 23 | 0,0407 | 0,0135 | 33,2386 | 0,0000 | 0,0000 |  |
| HIV Tat-specific factor | Htatsf1 | 86 | 0,0109 | 0,0033 | 30,7641 | 0,0000 | 0,0000 |  |
| 26S proteasome non-ATPase regulatory | Psmd4 | 41 | 0,0230 | 0,0044 | 18,9950 | 0,0000 | 0,0000 |  |
| ATP-dependent RNA helicase | Ddx42 | 102 | 0,0092 | 0,0042 | 45,4954 | 0,0000 | 0,0000 |  |
| Protein canopy homolog 3 | Cnpy3 | 31 | 0,0305 | 0,0143 | 47,0619 | 0,0000 | 0,0000 |  |
| Protein canopy homolog 4 | Cnpy4 | 28 | 0,0334 | 0,0151 | 45,2421 | 0,0000 | 0,0000 |  |
| Mitochondrial import inner membrane translocase | Pam16 | 14 | 0,0308 | 0,0134 | 43,4972 | 0,0538 | 0,0211 | 39,1752 |
| protein disulfide-isomerase | Creld1 | 46 | 0,0092 | 0,0032 | 34,4368 | 0,0157 | 0,0031 | 19,8798 |
| Collagen, type IV, alpha | Col4a2 | 167 | 0,0021 | 0,0001 | 4,6620 | 0,0057 | 0,0031 | 53,8332 |
| 3-dehydrosphinganine reductase | Kdsr | 34 | 0,0152 | 0,0061 | 39,8818 | 0,0179 | 0,0042 | 23,5822 |
| BCL2-antagonist/killer 1 | Bak1 | 16 | 0,0319 | 0,0118 | 36,9002 | 0,0381 | 0,0090 | 23,5822 |
| Casein kinase II subunit | Csnk2b | 19 | 0,0317 | 0,0174 | 55,0822 | 0,0217 | 0,0151 | 69,4697 |
| MICOS complex subunit Mic26 | Apoo | 23 | 0,0226 | 0,0095 | 41,9291 | 0,0244 | 0,0173 | 70,7018 |
| Erythrocyte membrane protein band 4.1 like | Epb41l1 | 98 | 0,0061 | 0,0017 | 27,8362 | 0,0042 | 0,0029 | 69,4697 |
| Lysophospholipase D GDPD1 | Gdpd1 | 36 | 0,0143 | 0,0057 | 39,8818 | 0,0154 | 0,0131 | 85,1082 |
| ELMO/CED-12 domain containing | Elmod2 | 22 | 0,0267 | 0,0133 | 49,8464 | 0,0188 | 0,0130 | 69,4697 |
| m-AAA protease-interacting protein | Maip1 | 33 | 0,0104 | 0,0005 | 4,6620 | 0,0267 | 0,0089 | 33,4900 |
| ABC-type antigen peptide | Tap1 | 79 | 0,0086 | 0,0031 | 36,6643 | 0,0038 | 0,0044 | 115,8293 |
| Large ribosomal subunit protein | Mrpl1 | 38 | 0,0136 | 0,0055 | 40,5573 | 0,0161 | 0,0038 | 23,5822 |
| Eukaryotic translation initiation factor | Eif4e | 15 | 0,0398 | 0,0111 | 27,8362 | 0,0254 | 0,0301 | 118,5050 |
| Sorting nexin 6 | Snx6 | 34 | 0,0202 | 0,0009 | 4,6620 | 0,0065 | 0,0130 | 200,0000 |
| Translocase of outer mitochondrial membrane 20 homolog | Tomm20 | 16 | 0,0375 | 0,0109 | 29,1608 | 0,0258 | 0,0179 | 69,4697 |
| Golgi SNAP receptor complex | Gosr2 | 20 | 0,0215 | 0,0169 | 78,5698 | 0,0360 | 0,0072 | 19,8798 |
| Syntaxin-4 | Stx4 | 34 | 0,0202 | 0,0009 | 4,6620 | 0,0079 | 0,0095 | 119,1500 |
| Sulfhydryl oxidase 2 | Qsox2 | 78 | 0,0088 | 0,0004 | 4,6620 | 0,0028 | 0,0057 | 200,0000 |
| Glycylpeptide N-tetradecanoyltransferase 1 | Nmt1 | 57 | 0,0076 | 0,0032 | 42,4231 | 0,0132 | 0,0052 | 39,1752 |
| Tricarboxylate transport protein, mitochondrial | Slc25a1 | 34 | 0,0149 | 0,0128 | 85,9337 | 0,0165 | 0,0117 | 70,7018 |
| Small nuclear ribonucleoprotein E | Snrpe | 11 | 0,0623 | 0,0029 | 4,6620 | 0,0230 | 0,0270 | 117,2710 |
| Monoacylglycerol lipase ABHD6 | Abhd6 | 38 | 0,0178 | 0,0124 | 69,5992 | 0,0067 | 0,0078 | 117,2710 |
| Alpha-crystallin B chain | Cryab | 20 | 0,0257 | 0,0171 | 66,5362 | 0,0289 | 0,0199 | 68,9748 |
| Protein RER1 | Rer1 | 23 | 0,0257 | 0,0130 | 50,4960 | 0,0179 | 0,0125 | 69,4697 |
| FUN14 domain-containing protein | Fundc2 | 17 | 0,0298 | 0,0199 | 66,8664 | 0,0308 | 0,0220 | 71,5447 |
| Acetyl-CoA carboxylase 1 | Acaca | 265 | 0,0025 | 0,0024 | 94,4328 | 0,0010 | 0,0011 | 117,2710 |
| NADH dehydrogenase [ubiquinone] 1 beta subcomplex | Ndufb10 | 21 | 0,0330 | 0,0143 | 43,3400 | 0,0121 | 0,0142 | 117,2710 |
| Transmembrane protein 87A | Tmem87a | 64 | 0,0067 | 0,0027 | 39,9794 | 0,0113 | 0,0022 | 19,8798 |
| Apoptosis inhibitor 5 | Api5 | 57 | 0,0135 | 0,0059 | 43,3954 | 0,0025 | 0,0050 | 200,0000 |
| CUGBP, Elav-like family | Celf1 | 52 | 0,0148 | 0,0033 | 22,4277 | 0,0031 | 0,0061 | 200,0000 |
| Collagen alpha-1(XI) | Col11a1 | 181 | 0,0042 | 0,0018 | 42,0554 | 0,0008 | 0,0016 | 200,0000 |
| Proteasome subunit beta | Psmb2 | 23 | 0,0263 | 0,0083 | 31,4263 | 0,0203 | 0,0149 | 73,2090 |
| PITH domain-containing protein | Txnl1 | 32 | 0,0240 | 0,0100 | 41,8240 | 0,0045 | 0,0089 | 200,0000 |
| RRM domain-containing protein | Dazap1 | 43 | 0,0200 | 0,0049 | 24,7343 | 0,0000 | 0,0000 |  |
| Src substrate cortactin | Cttn | 61 | 0,0140 | 0,0057 | 40,4609 | 0,0000 | 0,0000 |  |
| Protein NipSnap homolog 3B | Nipsnap3b | 28 | 0,0303 | 0,0153 | 50,3385 | 0,0000 | 0,0000 |  |
| Chromobox protein homolog 5 | Cbx5 | 22 | 0,0388 | 0,0087 | 22,3465 | 0,0000 | 0,0000 |  |
| Clathrin light chain | Clta | 23 | 0,0302 | 0,0223 | 73,8761 | 0,0134 | 0,0166 | 124,2177 |
| Transcriptional activator protein Pur | Pura | 35 | 0,0244 | 0,0052 | 21,5150 | 0,0000 | 0,0000 |  |
| Chromatin modifying protein 6 | Chmp6 | 23 | 0,0149 | 0,0007 | 4,6620 | 0,0327 | 0,0128 | 39,1752 |
| V-type proton ATPase subunit | Atp6ap1 | 51 | 0,0084 | 0,0036 | 42,4231 | 0,0120 | 0,0028 | 23,5822 |
| Sodium- and chloride-dependent taurine transporter | Slc6a6 | 70 | 0,0061 | 0,0024 | 39,9794 | 0,0087 | 0,0021 | 23,5822 |
| Beta-2-microglobulin | B2m | 14 | 0,0371 | 0,0156 | 41,9291 | 0,0295 | 0,0205 | 69,4697 |
| Pre-B-cell leukemia transcription factor-interacting | Pbxip1 | 81 | 0,0053 | 0,0023 | 43,4972 | 0,0075 | 0,0018 | 23,5822 |
| pre-rRNA 2'-O-ribose | Ftsj3 | 96 | 0,0044 | 0,0015 | 34,4368 | 0,0064 | 0,0015 | 23,5822 |
| Renin receptor | Atp6ap2 | 39 | 0,0133 | 0,0056 | 41,9291 | 0,0116 | 0,0085 | 73,8033 |
| Splicing factor 3B subunit | Sf3b6 | 15 | 0,0404 | 0,0233 | 57,6586 | 0,0169 | 0,0198 | 117,2710 |
| L-2-hydroxyglutarate dehydrogenase | L2hgdh | 51 | 0,0101 | 0,0041 | 40,5573 | 0,0078 | 0,0106 | 136,0264 |
| Thioredoxin domain-containing protein | Txndc15 | 38 | 0,0134 | 0,0047 | 35,1838 | 0,0131 | 0,0091 | 69,1296 |
| Growth hormone-inducible transmembrane | Ghitm | 37 | 0,0161 | 0,0044 | 27,1745 | 0,0068 | 0,0080 | 117,2710 |
| RNA-binding protein Luc7-like | Luc7l2 | 45 | 0,0096 | 0,0041 | 42,4231 | 0,0136 | 0,0032 | 23,5822 |
| 28S ribosomal protein S11 | Mrps11 | 20 | 0,0256 | 0,0096 | 37,6668 | 0,0182 | 0,0220 | 120,7865 |
| Transmembrane protein 70 | Tmem70 | 28 | 0,0154 | 0,0067 | 43,4972 | 0,0218 | 0,0051 | 23,5822 |
| Xaa-Pro aminopeptidase 3 | Xpnpep3 | 57 | 0,0075 | 0,0030 | 39,9794 | 0,0089 | 0,0104 | 117,2710 |
| Solute carrier family 7 (cationic amino acid transporter, y+ system), member 1 (Fragment | Slc7a1 | 58 | 0,0117 | 0,0044 | 37,5128 | 0,0019 | 0,0038 | 200,0000 |
| dynamin GTPase | Dnm2 | 98 | 0,0061 | 0,0045 | 74,0247 | 0,0023 | 0,0045 | 200,0000 |
| Mrpl20 protein | Mrpl20 | 18 | 0,0331 | 0,0090 | 27,1745 | 0,0150 | 0,0179 | 119,1500 |
| Membrane associated ring-CH-type | Marchf5 | 27 | 0,0257 | 0,0113 | 44,0380 | 0,0041 | 0,0082 | 200,0000 |
| Endoplasmic reticulum protein SC65 | P3h4 | 51 | 0,0117 | 0,0033 | 27,8362 | 0,0056 | 0,0112 | 200,0000 |
| AP-1 complex subunit gamma | Ap1g1 | 91 | 0,0075 | 0,0055 | 72,7980 | 0,0012 | 0,0024 | 200,0000 |
| Dynactin subunit 2 | Dctn2 | 44 | 0,0118 | 0,0083 | 70,2257 | 0,0095 | 0,0110 | 115,8630 |
| Glutathione hydrolase 7 | Ggt7 | 70 | 0,0076 | 0,0088 | 116,8579 | 0,0060 | 0,0069 | 115,8630 |
| Small ribosomal subunit protein m | Mrps25 | 19 | 0,0272 | 0,0110 | 40,5573 | 0,0217 | 0,0151 | 69,4697 |
| Calcium/calmodulin-dependent serine protein kinase (MAGUK family) | Cask | 99 | 0,0077 | 0,0013 | 17,1468 | 0,0000 | 0,0000 |  |
| Cell growth-regulating nucleolar | Lyar | 44 | 0,0178 | 0,0105 | 59,0930 | 0,0000 | 0,0000 |  |
| Volume-regulated anion channel subunit | Lrrc8c | 92 | 0,0083 | 0,0033 | 40,0364 | 0,0000 | 0,0000 |  |
| Pyruvate dehydrogenase protein X component | Pdhx | 54 | 0,0110 | 0,0080 | 72,3366 | 0,0047 | 0,0055 | 117,2710 |
| Transmembrane protein 11 | Tmem11 | 20 | 0,0207 | 0,0200 | 96,6815 | 0,0294 | 0,0404 | 137,4807 |
| AU RNA binding protein/enoyl-coenzyme | Auh | 33 | 0,0156 | 0,0062 | 39,8818 | 0,0137 | 0,0101 | 73,8033 |
| Tppp3 protein (Fragment | Tppp3 | 17 | 0,0450 | 0,0185 | 41,1418 | 0,0000 | 0,0000 |  |
| EF-hand domain-containing | Rcn2 | 41 | 0,0150 | 0,0148 | 98,8221 | 0,0054 | 0,0108 | 200,0000 |
| Transcription elongation factor | Tcea1 | 35 | 0,0218 | 0,0087 | 40,0364 | 0,0000 | 0,0000 |  |
| Annexin | Anxa6 | 75 | 0,0068 | 0,0024 | 35,1838 | 0,0067 | 0,0046 | 69,1296 |
| Small integral membrane protein | Smim8 | 11 | 0,0387 | 0,0392 | 101,2376 | 0,0539 | 0,0443 | 82,1495 |
| ABC transporter domain-containing | Abcf2 | 72 | 0,0094 | 0,0034 | 36,6643 | 0,0015 | 0,0031 | 200,0000 |
| Mitochondrial ribosomal protein L23 | Mrpl23 | 20 | 0,0339 | 0,0191 | 56,3756 | 0,0071 | 0,0143 | 200,0000 |
| Small ribosomal subunit protein | Rps29 | 7 | 0,1092 | 0,0449 | 41,1418 | 0,0000 | 0,0000 |  |
| Transmembrane protein 245 | Tmem245 | 97 | 0,0071 | 0,0030 | 41,9640 | 0,0011 | 0,0023 | 200,0000 |
| Septin | Septin11 | 49 | 0,0139 | 0,0097 | 69,9543 | 0,0000 | 0,0000 |  |
| Transmembrane protein 65 | Tmem65 | 25 | 0,0172 | 0,0073 | 42,4231 | 0,0165 | 0,0115 | 69,4697 |
| Induced myeloid leukemia cell differentiation protein Mcl | Mcl1 | 35 | 0,0123 | 0,0052 | 42,4231 | 0,0118 | 0,0082 | 69,4697 |
| Protein odr-4 homolog | Odr4 | 38 | 0,0088 | 0,0069 | 78,5634 | 0,0161 | 0,0038 | 23,5822 |
| Titin | Ttn | 3906 | 0,0001 | 0,0001 | 96,6815 | 0,0002 | 0,0000 | 23,5822 |
| Uncharacterized protein | Mavs | 34 | 0,0124 | 0,0043 | 34,4368 | 0,0137 | 0,0101 | 73,2090 |
| Reticulophagy regulator 3 | Retreg3 | 52 | 0,0066 | 0,0003 | 4,6620 | 0,0101 | 0,0072 | 71,5447 |
| Interferon-gamma-inducible GTPase Ifgga | Iigp1 | 48 | 0,0106 | 0,0037 | 35,1838 | 0,0053 | 0,0062 | 117,2710 |
| Tyrosine-protein kinase receptor | Axl | 98 | 0,0053 | 0,0021 | 40,5573 | 0,0026 | 0,0030 | 117,2710 |
| Motile sperm domain containing | Mospd2 | 60 | 0,0042 | 0,0028 | 66,9175 | 0,0120 | 0,0024 | 19,8798 |
| Small nuclear ribonucleoprotein polypeptide | Snrpa1 | 18 | 0,0284 | 0,0105 | 36,9002 | 0,0171 | 0,0213 | 124,2177 |
| 40S ribosomal protein S15 | Rps15 | 14 | 0,0426 | 0,0116 | 27,1745 | 0,0079 | 0,0158 | 200,0000 |
| Synaptojanin-2-binding protein | Synj2bp | 16 | 0,0263 | 0,0197 | 75,0971 | 0,0228 | 0,0275 | 120,7865 |
| Endoplasmic reticulum-Golgi intermediate compartment | Ergic2 | 42 | 0,0100 | 0,0075 | 75,3732 | 0,0094 | 0,0128 | 136,0264 |
| Dystroglycan 1 | Dag1 | 97 | 0,0009 | 0,0018 | 200,0000 | 0,0102 | 0,0038 | 37,4650 |
| Nuclear protein localization protein | Nploc4 | 68 | 0,0062 | 0,0046 | 75,3732 | 0,0056 | 0,0066 | 118,5050 |
| Hydroxysteroid dehydrogenase like 1 | Hsdl1 | 34 | 0,0149 | 0,0053 | 35,1838 | 0,0079 | 0,0095 | 119,1500 |
| Phosphatidylethanolamine binding protein 1 | Pebp1 | 23 | 0,0149 | 0,0007 | 4,6620 | 0,0282 | 0,0290 | 102,8556 |
| Lysophosphatidylcholine acyltransferase 1 | Lpcat1 | 60 | 0,0100 | 0,0028 | 27,8362 | 0,0024 | 0,0048 | 200,0000 |
| WD40 repeat-containing protein | Smu1 | 58 | 0,0088 | 0,0031 | 35,1838 | 0,0044 | 0,0051 | 117,2710 |
| FERM, ARHGEF and pleckstrin domain-containing | Farp1 | 119 | 0,0036 | 0,0037 | 102,7085 | 0,0036 | 0,0051 | 139,9501 |
| Myosin regulatory light chain | Myl12b | 20 | 0,0212 | 0,0163 | 76,6816 | 0,0206 | 0,0143 | 69,4697 |
| Small ribosomal subunit protein | Mrps17 | 13 | 0,0463 | 0,0255 | 55,0822 | 0,0085 | 0,0170 | 200,0000 |
| Transmembrane emp24 domain containing | Tmed1 | 25 | 0,0242 | 0,0076 | 31,4263 | 0,0044 | 0,0089 | 200,0000 |
| Large ribosomal subunit protein | Mrpl9 | 30 | 0,0140 | 0,0105 | 75,0971 | 0,0121 | 0,0147 | 120,7865 |
| UPF0598 protein |  | 24 | 0,0252 | 0,0079 | 31,4263 | 0,0046 | 0,0092 | 200,0000 |
| FAD-dependent oxidoreductase domain-containing | Foxred1 | 54 | 0,0080 | 0,0034 | 42,4231 | 0,0071 | 0,0084 | 118,5050 |
| Glutathione S-transferase (Fragment | Gsta3 | 23 | 0,0114 | 0,0076 | 66,7067 | 0,0365 | 0,0295 | 80,8613 |
| NADH dehydrogenase [ubiquinone] iron-sulfur protein | Ndufs3 | 30 | 0,0202 | 0,0117 | 57,6586 | 0,0037 | 0,0074 | 200,0000 |
| Sntb2 protein | Sntb2 | 57 | 0,0046 | 0,0059 | 128,5592 | 0,0117 | 0,0086 | 73,4444 |
| Atlastin GTPase 2 | Atl2 | 47 | 0,0091 | 0,0069 | 76,0432 | 0,0089 | 0,0103 | 115,8630 |
| NADH dehydrogenase [ubiquinone] iron-sulfur protein | Ndufs6 | 13 | 0,0527 | 0,0025 | 4,6620 | 0,0000 | 0,0000 |  |
| Cystatin-C | Cst3 | 16 | 0,0426 | 0,0172 | 40,2863 | 0,0000 | 0,0000 |  |
| Synaptotagmin-4 | Syt4 | 48 | 0,0143 | 0,0007 | 4,6620 | 0,0000 | 0,0000 |  |
| Annexin | Anxa1 | 39 | 0,0175 | 0,0074 | 41,9640 | 0,0000 | 0,0000 |  |
| Endoplasmic reticulum lectin 1 | Erlec1 | 55 | 0,0125 | 0,0054 | 42,9258 | 0,0000 | 0,0000 |  |
| Small ribosomal subunit protein | Mrps28 | 21 | 0,0328 | 0,0142 | 43,3459 | 0,0000 | 0,0000 |  |
| Histone deacetylase 2 | Hdac2 | 55 | 0,0061 | 0,0048 | 78,5634 | 0,0108 | 0,0089 | 82,1495 |
| Cytochrome b-c1 complex | Uqcrb | 14 | 0,0483 | 0,0177 | 36,6643 | 0,0000 | 0,0000 | #DIV/0! |
| Protein-L-isoaspartate O | Pcmt1 | 30 | 0,0226 | 0,0128 | 56,3756 | 0,0000 | 0,0000 | #DIV/0! |
| Alpha-soluble NSF attachment | Napa | 33 | 0,0180 | 0,0096 | 53,5801 | 0,0043 | 0,0086 | 200,0000 |
| Vitamin K epoxide reductase complex subunit 1-like protein | Vkorc1l1 | 20 | 0,0255 | 0,0094 | 36,9002 | 0,0135 | 0,0161 | 119,1500 |
| S-adenosyl-L-methionine-dependent tRNA 4-demethylwyosine | Tyw1 | 82 | 0,0073 | 0,0020 | 27,8362 | 0,0014 | 0,0027 | 200,0000 |
| Cluster of Talin-1 | Tln1 | 270 | 0,0016 | 0,0017 | 102,0472 | 0,0017 | 0,0013 | 73,2090 |
| Choline transporter-like protein | Slc44a1 | 59 | 0,0045 | 0,0057 | 127,2653 | 0,0113 | 0,0083 | 73,4444 |
| Large ribosomal subunit protein | Mrpl47 | 30 | 0,0198 | 0,0143 | 72,4765 | 0,0053 | 0,0106 | 200,0000 |
| Actin-related protein 2/3 complex subunit | Arpc5 | 10 | 0,0511 | 0,0188 | 36,9002 | 0,0253 | 0,0297 | 117,2710 |
| Poly(A) binding protein, nuclear | Pabpn1 | 18 | 0,0378 | 0,0142 | 37,5128 | 0,0000 | 0,0000 |  |
| Lysophosphatidylserine lipase ABHD12 | Abhd12 | 45 | 0,0132 | 0,0096 | 72,3366 | 0,0025 | 0,0049 | 200,0000 |
| Septin 9 | Septin9 | 64 | 0,0082 | 0,0072 | 88,6326 | 0,0040 | 0,0046 | 117,2710 |
| Thymidylate kinase | Dtymk | 24 | 0,0284 | 0,0199 | 69,9543 | 0,0000 | 0,0000 |  |
| Clathrin light chain | Cltb | 23 | 0,0298 | 0,0323 | 108,2341 | 0,0000 | 0,0000 |  |
| peptidylprolyl isomerase | Fkbp3 | 25 | 0,0281 | 0,0347 | 123,5654 | 0,0000 | 0,0000 |  |
| Polypyrimidine tract binding protein | Ptbp3 | 60 | 0,0099 | 0,0053 | 53,5801 | 0,0033 | 0,0066 | 200,0000 |
| Prostaglandin E synthase 2 | Ptges2 | 43 | 0,0157 | 0,0111 | 70,6476 | 0,0000 | 0,0000 |  |
| Large ribosomal subunit protein | Mrpl24 | 25 | 0,0137 | 0,0006 | 4,6620 | 0,0146 | 0,0176 | 120,7865 |
| Integrin alpha-4 | Itga4 | 117 | 0,0036 | 0,0013 | 34,4368 | 0,0023 | 0,0028 | 119,1500 |
| Complement component (3b/4b) receptor 1-like (Fragment) | Cr1l | 29 | 0,0087 | 0,0058 | 66,8664 | 0,0194 | 0,0137 | 70,7018 |
| Translocase of inner mitochondrial membrane 17b (Fragment) | Timm17b | 16 | 0,0320 | 0,0120 | 37,6668 | 0,0069 | 0,0139 | 200,0000 |
| Voltage-gated monoatomic cation | Tmem109 | 26 | 0,0166 | 0,0070 | 42,4231 | 0,0110 | 0,0219 | 200,0000 |
| KH domain-containing RNA-binding | Qki | 38 | 0,0111 | 0,0038 | 34,4368 | 0,0067 | 0,0078 | 117,2710 |
| Large ribosomal subunit protein m | Mrpl44 | 33 | 0,0128 | 0,0044 | 34,4368 | 0,0067 | 0,0134 | 200,0000 |
| Signal recognition particle 14 kDa | Srp14 | 13 | 0,0335 | 0,0255 | 76,0175 | 0,0208 | 0,0248 | 119,1500 |
| Perilipin-2 | Plin2 | 47 | 0,0107 | 0,0065 | 60,8011 | 0,0024 | 0,0047 | 200,0000 |
| Protein arginine N-methyltransferase | Prmt1 | 29 | 0,0146 | 0,0050 | 34,4368 | 0,0087 | 0,0102 | 117,2710 |
| Signal recognition particle subunit | Srp68 | 66 | 0,0064 | 0,0065 | 101,2376 | 0,0038 | 0,0045 | 117,2710 |
| Serine/threonine-protein phosphatase 2A 65 kDa regulatory subunit A alpha isoform OS=Mus | Ppp2r1a | 65 | 0,0065 | 0,0022 | 34,4368 | 0,0039 | 0,0046 | 117,2710 |
| Dimethyladenosine transferase 1, mitochondrial | Tfb1m | 39 | 0,0133 | 0,0054 | 40,5573 | 0,0028 | 0,0057 | 200,0000 |
| Ran GTPase-activating protein | Rangap1 | 64 | 0,0068 | 0,0071 | 103,6888 | 0,0042 | 0,0050 | 119,1500 |
| Phenylalanine--tRNA ligase alpha | Farsa | 58 | 0,0087 | 0,0053 | 60,8011 | 0,0027 | 0,0055 | 200,0000 |
| DnaJ homolog subfamily | Dnajb12 | 42 | 0,0103 | 0,0082 | 78,8489 | 0,0053 | 0,0106 | 200,0000 |
| Cluster of calcium/calmodulin-dependent protein kinase | Camk2d | 60 | 0,0070 | 0,0053 | 75,0971 | 0,0037 | 0,0074 | 200,0000 |
| Rho GDP-dissociation inhibitor | Arhgdia | 23 | 0,0263 | 0,0083 | 31,4263 | 0,0000 | 0,0000 |  |
| Proteasome subunit beta type | Psmb1 | 26 | 0,0227 | 0,0115 | 50,4960 | 0,0000 | 0,0000 |  |
| Bcl-2-like protein | Bcl2l13 | 47 | 0,0089 | 0,0067 | 75,3732 | 0,0047 | 0,0094 | 200,0000 |
| Stromal cell-derived factor 2-like protein | Sdf2l1 | 24 | 0,0248 | 0,0068 | 27,1745 | 0,0000 | 0,0000 |  |
| Collagen, type IX, alpha | Col9a2 | 65 | 0,0092 | 0,0026 | 27,8362 | 0,0000 | 0,0000 |  |
| RRM domain-containing protein | Slirp | 13 | 0,0459 | 0,0125 | 27,1745 | 0,0000 | 0,0000 |  |
| Eukaryotic translation initiation factor 3 subunit | Eif3h | 40 | 0,0149 | 0,0042 | 27,8362 | 0,0000 | 0,0000 |  |
| Interleukin enhancer binding factor | Ilf3 | 97 | 0,0043 | 0,0041 | 96,3646 | 0,0023 | 0,0046 | 200,0000 |
| RIKEN cDNA 9530068E07 gene | 9530068E07Rik | 27 | 0,0126 | 0,0103 | 82,4273 | 0,0135 | 0,0163 | 120,7865 |
| BCL2-associated transcription factor | Bclaf1 | 86 | 0,0060 | 0,0053 | 87,8155 | 0,0013 | 0,0026 | 200,0000 |
| Transmembrane protein 230 | Tmem230 | 13 | 0,0327 | 0,0332 | 101,5644 | 0,0208 | 0,0248 | 119,1500 |
| Tumor necrosis factor receptor superfamily | Ngfr | 45 | 0,0114 | 0,0043 | 37,6668 | 0,0032 | 0,0063 | 200,0000 |
| Lysophosphatidic acid phosphatase type | Acp6 | 48 | 0,0125 | 0,0036 | 29,1608 | 0,0000 | 0,0000 |  |
| Derlin | Derl2 | 19 | 0,0131 | 0,0163 | 124,8768 | 0,0259 | 0,0334 | 128,9647 |
| MICOS complex subunit Mic19 | Chchd3 | 26 | 0,0195 | 0,0162 | 82,9791 | 0,0043 | 0,0085 | 200,0000 |
| Reticulon 4 interacting protein | Rtn4ip1 | 43 | 0,0078 | 0,0061 | 78,5634 | 0,0105 | 0,0077 | 73,8033 |
| Cytochrome c oxidase subunit 5A, mitochondrial | Cox5a | 16 | 0,0273 | 0,0209 | 76,4675 | 0,0212 | 0,0251 | 118,4137 |
| Golgi reassembly stacking protein | Gorasp2 | 37 | 0,0142 | 0,0095 | 66,7067 | 0,0043 | 0,0086 | 200,0000 |
| Large ribosomal subunit protein | Mrpl41 | 15 | 0,0403 | 0,0127 | 31,4263 | 0,0000 | 0,0000 |  |
| CCR4-NOT transcription complex | Cnot1 | 266 | 0,0010 | 0,0006 | 66,8664 | 0,0018 | 0,0024 | 132,6393 |
| ATP-dependent RNA helicase | Ddx19a | 54 | 0,0110 | 0,0059 | 53,5801 | 0,0000 | 0,0000 |  |
| SURF1-like protein (Fragment | Surf1 | 27 | 0,0188 | 0,0126 | 66,8664 | 0,0041 | 0,0082 | 200,0000 |
| Fibrinogen C-terminal domain-containing | Fgg | 49 | 0,0053 | 0,0067 | 126,3917 | 0,0139 | 0,0164 | 118,4137 |
| Anoctamin-10 | Ano10 | 76 | 0,0067 | 0,0078 | 116,0239 | 0,0015 | 0,0029 | 200,0000 |
| Protein OS-9 | Os9 | 76 | 0,0067 | 0,0024 | 35,1838 | 0,0021 | 0,0042 | 200,0000 |
| Twinfilin-1 | Twf1 | 40 | 0,0147 | 0,0073 | 49,8464 | 0,0000 | 0,0000 |  |
| Phospholipid/glycerol acyltransferase domain-containing | Agpat5 | 42 | 0,0103 | 0,0045 | 43,4972 | 0,0060 | 0,0071 | 117,2710 |
| Plastin 3 (T-isoform) | Pls3 | 72 | 0,0084 | 0,0047 | 55,8350 | 0,0000 | 0,0000 |  |
| Golgi autoantigen, golgin subfamily | Golga2 | 115 | 0,0052 | 0,0038 | 73,1220 | 0,0000 | 0,0000 |  |
| Hepatoma-derived growth factor | Hdgf | 26 | 0,0226 | 0,0158 | 70,0586 | 0,0000 | 0,0000 |  |
| Large ribosomal subunit protein | Mrpl14 | 16 | 0,0314 | 0,0191 | 60,8011 | 0,0069 | 0,0139 | 200,0000 |
| V-type proton ATPase subunit | Tcirg1 | 93 | 0,0037 | 0,0031 | 84,7863 | 0,0039 | 0,0047 | 120,7865 |
| Cluster of Importin-5 | Ipo5 | 124 | 0,0035 | 0,0027 | 76,0175 | 0,0022 | 0,0026 | 119,1500 |
| Cell cycle control protein | Tmem30a | 37 | 0,0114 | 0,0087 | 76,4632 | 0,0092 | 0,0109 | 118,4137 |
| Metalloproteinase inhibitor 3 | Timp3 | 24 | 0,0245 | 0,0122 | 49,8464 | 0,0000 | 0,0000 |  |
| Hyaluronan mediated motility receptor | Hmmr | 92 | 0,0047 | 0,0020 | 43,4972 | 0,0012 | 0,0024 | 200,0000 |
| DnaJ homolog subfamily | Dnajc25 | 42 | 0,0103 | 0,0043 | 42,4231 | 0,0026 | 0,0053 | 200,0000 |
| Golgin subfamily A member | Golga5 | 82 | 0,0021 | 0,0024 | 115,7943 | 0,0064 | 0,0046 | 71,5447 |
| N(alpha)-acetyltransferase 15, NatA auxiliary | Naa15 | 101 | 0,0025 | 0,0017 | 66,9175 | 0,0038 | 0,0045 | 118,5050 |
| Uncharacterized protein | Ptpmt1 | 29 | 0,0119 | 0,0101 | 84,7863 | 0,0093 | 0,0111 | 119,1500 |
| Peroxiredoxin like 2A (Fragment) | Prxl2a | 23 | 0,0186 | 0,0074 | 39,9794 | 0,0062 | 0,0124 | 200,0000 |
| Septin-2 | Septin2 | 37 | 0,0116 | 0,0091 | 78,5698 | 0,0030 | 0,0060 | 200,0000 |
| GPI-anchor transamidase | Pigk | 41 | 0,0086 | 0,0071 | 82,7762 | 0,0054 | 0,0108 | 200,0000 |
| Cytosolic 5'-nucleotidase 3A | Nt5c3a | 37 | 0,0119 | 0,0092 | 77,3875 | 0,0030 | 0,0060 | 200,0000 |
| Vacuolar protein sorting-associated protein | Vps29 | 14 | 0,0306 | 0,0122 | 39,9794 | 0,0141 | 0,0282 | 200,0000 |
| Lysozyme f3 | Lyz1 | 17 | 0,0157 | 0,0200 | 128,0524 | 0,0214 | 0,0259 | 120,7865 |
| FAD-linked sulfhydryl oxidase | Gfer | 26 | 0,0221 | 0,0078 | 35,1838 | 0,0000 | 0,0000 |  |
| SET nuclear oncogene (Fragment) | Set | 25 | 0,0203 | 0,0072 | 35,1838 | 0,0000 | 0,0000 |  |
| NADH dehydrogenase [ubiquinone] 1 alpha subcomplex | Ndufa2 | 11 | 0,0462 | 0,0163 | 35,1838 | 0,0000 | 0,0000 |  |
| Prpsap2 protein | Prpsap2 | 31 | 0,0138 | 0,0055 | 39,9794 | 0,0046 | 0,0092 | 200,0000 |
| NPC intracellular cholesterol transporter | Npc1 | 143 | 0,0036 | 0,0013 | 36,9002 | 0,0000 | 0,0000 |  |
| Disintegrin and metalloproteinase domain-containing | Adam10 | 84 | 0,0051 | 0,0020 | 39,9794 | 0,0013 | 0,0026 | 200,0000 |
| Nucleolar protein 16 | Nop16 | 22 | 0,0193 | 0,0148 | 76,6816 | 0,0050 | 0,0101 | 200,0000 |
| Pre-mRNA-processing factor | Prpf19 | 55 | 0,0093 | 0,0035 | 37,6668 | 0,0000 | 0,0000 |  |
| Sorting nexin-3 | Snx3 | 19 | 0,0265 | 0,0161 | 60,8011 | 0,0000 | 0,0000 |  |
| NIF3-like protein 1 | Nif3l1 | 36 | 0,0120 | 0,0052 | 43,4972 | 0,0040 | 0,0079 | 200,0000 |
| Transcription factor BTF3 | Btf3 | 18 | 0,0284 | 0,0105 | 36,9002 | 0,0000 | 0,0000 |  |
| Transcription factor SOX-10 | Sox10 | 50 | 0,0086 | 0,0067 | 78,5698 | 0,0022 | 0,0044 | 200,0000 |
| Phosphatidate cytidylyltransferase, mitochondrial | Tamm41 | 38 | 0,0092 | 0,0073 | 80,2392 | 0,0067 | 0,0078 | 117,2710 |
| Ubiquitin-fold modifier-conjugating | Ufc1 | 11 | 0,0470 | 0,0191 | 40,5573 | 0,0000 | 0,0000 |  |
| Transketolase | Tkt | 68 | 0,0037 | 0,0048 | 129,7019 | 0,0066 | 0,0049 | 73,8033 |
| Collagen triple helix repeat | Cthrc1 | 26 | 0,0196 | 0,0072 | 36,9002 | 0,0000 | 0,0000 |  |
| Syntaxin 6 | Stx6 | 23 | 0,0107 | 0,0134 | 125,7098 | 0,0172 | 0,0234 | 136,0264 |
| U6 snRNA-associated Sm-like protein | Lsm6 | 9 | 0,0573 | 0,0229 | 39,8818 | 0,0000 | 0,0000 |  |
| Small ribosomal subunit protein | Mrps31 | 44 | 0,0117 | 0,0047 | 39,8818 | 0,0000 | 0,0000 |  |
| MIA SH3 domain ER export | Mia2 | 157 | 0,0027 | 0,0020 | 75,5484 | 0,0007 | 0,0014 | 200,0000 |
| Thioredoxin 2 | Txn2 | 16 | 0,0326 | 0,0229 | 70,2257 | 0,0000 | 0,0000 |  |
| Discoidin domain-containing receptor | Ddr2 | 96 | 0,0036 | 0,0030 | 84,1799 | 0,0000 | 0,0000 |  |
| Exonuclease domain-containing protein | Rexo2 | 27 | 0,0185 | 0,0154 | 82,9870 | 0,0000 | 0,0000 |  |
| Isoleucine--tRNA ligase, cytoplasmic | Iars1 | 144 | 0,0029 | 0,0022 | 76,4632 | 0,0010 | 0,0020 | 200,0000 |
| Transforming growth factor beta regulated | Tbrg4 | 42 | 0,0080 | 0,0115 | 143,7033 | 0,0064 | 0,0077 | 119,1500 |
| Phospholipase A-2-activating | Plaa | 87 | 0,0020 | 0,0040 | 200,0000 | 0,0065 | 0,0046 | 70,7018 |
| Pre-rRNA-processing protein | Tsr1 | 92 | 0,0047 | 0,0037 | 78,5698 | 0,0015 | 0,0031 | 200,0000 |
| Translational activator of cytochrome c | Taco1 | 32 | 0,0157 | 0,0131 | 83,7233 | 0,0000 | 0,0000 |  |
| Large ribosomal subunit protein | Mrpl12 | 22 | 0,0234 | 0,0204 | 87,0951 | 0,0000 | 0,0000 |  |
| receptor protein-tyrosine kinase | Insrr | 79 | 0,0030 | 0,0061 | 200,0000 | 0,0042 | 0,0084 | 200,0000 |
| Protein FMC1 homolog | Fmc1 | 13 | 0,0331 | 0,0141 | 42,4231 | 0,0085 | 0,0170 | 200,0000 |
| NADH dehydrogenase [ubiquinone] iron-sulfur protein | Ndufs4 | 20 | 0,0215 | 0,0091 | 42,4231 | 0,0000 | 0,0000 |  |
| Stromal cell derived factor | Sdf2 | 24 | 0,0178 | 0,0071 | 39,9794 | 0,0000 | 0,0000 |  |
| Low density lipoprotein receptor-related | Lrp1 | 505 | 0,0008 | 0,0003 | 39,9794 | 0,0000 | 0,0000 |  |
| Eukaryotic translation initiation factor 3 subunit | Eif3m | 43 | 0,0100 | 0,0040 | 39,9794 | 0,0000 | 0,0000 |  |
| Glutathione reductase (Fragment | Gsr | 53 | 0,0081 | 0,0035 | 43,4972 | 0,0000 | 0,0000 |  |
| B-cell receptor-associated | Bcap29 | 28 | 0,0092 | 0,0061 | 66,9551 | 0,0079 | 0,0158 | 200,0000 |
| Beta-galactosidase | Glb1 | 73 | 0,0058 | 0,0020 | 34,4368 | 0,0000 | 0,0000 |  |
| Amyloid beta (A4) precursor-like protein | Aplp2 | 71 | 0,0047 | 0,0037 | 78,5634 | 0,0020 | 0,0040 | 200,0000 |
| Inosine-5'-monophosphate dehydrogenase | Impdh2 | 56 | 0,0075 | 0,0026 | 34,4368 | 0,0000 | 0,0000 |  |
| RAN binding protein 1 | Ranbp1 | 18 | 0,0241 | 0,0190 | 78,8489 | 0,0000 | 0,0000 |  |
| Calcium load-activated calcium channel | Tmco1 | 22 | 0,0157 | 0,0131 | 83,3784 | 0,0050 | 0,0101 | 200,0000 |
| ER membrane protein complex | Emc3 | 30 | 0,0141 | 0,0049 | 34,4368 | 0,0000 | 0,0000 |  |
| Small ribosomal subunit protein | Mrps6 | 14 | 0,0307 | 0,0241 | 78,5698 | 0,0000 | 0,0000 |  |
| Sorting nexin-27 | Snx27 | 61 | 0,0055 | 0,0043 | 78,3332 | 0,0018 | 0,0036 | 200,0000 |
| UbiE1 | Tmt1a | 28 | 0,0151 | 0,0115 | 76,4632 | 0,0000 | 0,0000 |  |
| PIH1 domain-containing protein | Pih1d1 | 32 | 0,0132 | 0,0045 | 34,4368 | 0,0000 | 0,0000 |  |
| Cytoplasmic FMR1-interacting protein | Cyfip1 | 145 | 0,0024 | 0,0020 | 83,3784 | 0,0015 | 0,0031 | 200,0000 |
| Adrenodoxin, mitochondrial | Fdx1 | 20 | 0,0213 | 0,0161 | 75,5484 | 0,0000 | 0,0000 |  |
| ATP-binding cassette sub-family | Abce1 | 67 | 0,0065 | 0,0050 | 76,4675 | 0,0000 | 0,0000 |  |
| PRA1 family protein 2 | Praf2 | 19 | 0,0222 | 0,0170 | 76,4632 | 0,0000 | 0,0000 |  |
| Tripeptidyl-peptidase 2 | Tpp2 | 97 | 0,0044 | 0,0035 | 78,3979 | 0,0000 | 0,0000 |  |
| AP2-associated protein kinase | Aak1 | 103 | 0,0043 | 0,0043 | 101,0730 | 0,0000 | 0,0000 |  |
| Beta-hexosaminidase subunit alpha | Hexa | 61 | 0,0069 | 0,0024 | 34,4368 | 0,0000 | 0,0000 |  |
| Activated RNA polymerase II transcriptional coactivator | Sub1 | 14 | 0,0301 | 0,0230 | 76,4632 | 0,0000 | 0,0000 |  |
| SWI/SNF-related matrix-associated actin-dependent regulator of chromatin subfamily A containing | Smarcad1 | 116 | 0,0022 | 0,0015 | 66,8664 | 0,0019 | 0,0038 | 200,0000 |
| BET1 homolog | Bet1 | 13 | 0,0259 | 0,0299 | 115,7215 | 0,0085 | 0,0170 | 200,0000 |
| Integrin-linked kinase-associated serine/threonine | Ilkap | 43 | 0,0099 | 0,0075 | 75,5484 | 0,0000 | 0,0000 |  |
| 3-keto-steroid reductase/17-beta-hydroxysteroid dehydrogenase | Hsd17b7 | 37 | 0,0095 | 0,0078 | 81,9888 | 0,0030 | 0,0060 | 200,0000 |
| Polypeptide N-acetylgalactosaminyltransferase | Galnt12 | 67 | 0,0013 | 0,0027 | 200,0000 | 0,0084 | 0,0059 | 70,7018 |
| Protein ABHD11 | Abhd11 | 34 | 0,0126 | 0,0098 | 78,3979 | 0,0000 | 0,0000 |  |
| Ataxin 2-like (Fragment | Atxn2l | 92 | 0,0046 | 0,0016 | 34,4368 | 0,0000 | 0,0000 |  |
| CDP-diacylglycerol--glycerol-3-phosphate | Pgs1 | 62 | 0,0054 | 0,0042 | 77,7930 | 0,0023 | 0,0046 | 200,0000 |
| 55 kDa erythrocyte membrane | Mpp1 | 51 | 0,0085 | 0,0037 | 43,4972 | 0,0000 | 0,0000 |  |
| Beta-glucuronidase | Gusb | 74 | 0,0058 | 0,0023 | 39,9794 | 0,0000 | 0,0000 |  |
| Zinc finger CCCH domain-containing protein | Zc3h15 | 48 | 0,0092 | 0,0071 | 77,3875 | 0,0000 | 0,0000 |  |
| Replication protein A subunit | Rpa1 | 71 | 0,0062 | 0,0075 | 119,7322 | 0,0000 | 0,0000 |  |
| Protein scribble homolog | Scrib | 174 | 0,0020 | 0,0017 | 83,3784 | 0,0000 | 0,0000 |  |
| Protein FAM136A | Fam136a | 16 | 0,0213 | 0,0175 | 82,1774 | 0,0000 | 0,0000 |  |
| Fumarylacetoacetate hydrolase domain containing | Fahd2a | 35 | 0,0098 | 0,0083 | 84,7863 | 0,0000 | 0,0000 |  |
| Plakophilin-1 | Pkp1 | 81 | 0,0010 | 0,0020 | 200,0000 | 0,0053 | 0,0074 | 139,9501 |
| Ubiquitin-associated protein 2 | Ubap2 | 118 | 0,0029 | 0,0025 | 84,1799 | 0,0000 | 0,0000 |  |
| NADH dehydrogenase [ubiquinone] iron-sulfur protein | Ndufs5 | 11 | 0,0304 | 0,0238 | 78,3332 | 0,0000 | 0,0000 |  |
| U5 small nuclear ribonucleoprotein 40 kDa | Snrnp40 | 39 | 0,0090 | 0,0074 | 81,9888 | 0,0000 | 0,0000 |  |
| Zinc binding alcohol dehydrogenase, domain | Ptgr3 | 41 | 0,0081 | 0,0064 | 78,5634 | 0,0000 | 0,0000 |  |
| Synaptophysin-like protein 1 | Sypl1 | 29 | 0,0116 | 0,0090 | 77,7930 | 0,0000 | 0,0000 |  |
| Ubiquitin-conjugating enzyme E2I | Ube2i | 12 | 0,0283 | 0,0233 | 82,1774 | 0,0000 | 0,0000 |  |
| Sarcosine dehydrogenase (Fragment | Sardh | 100 | 0,0026 | 0,0033 | 126,3917 | 0,0014 | 0,0029 | 200,0000 |
| U6 snRNA-associated Sm-like protein | Lsm4 | 15 | 0,0223 | 0,0175 | 78,3332 | 0,0000 | 0,0000 |  |
| Proteasome subunit beta | Psmb3 | 23 | 0,0145 | 0,0114 | 78,5634 | 0,0000 | 0,0000 |  |
| Profilin | Pfn1 | 15 | 0,0225 | 0,0175 | 77,7930 | 0,0000 | 0,0000 |  |
| Aflatoxin B1 aldehyde reductase member | Akr7a2 | 41 | 0,0039 | 0,0078 | 200,0000 | 0,0062 | 0,0072 | 117,2710 |
| Cluster of Retinoic acid early transcript | Raet1e | 28 | 0,0029 | 0,0057 | 200,0000 | 0,0130 | 0,0157 | 120,7865 |
| Guanine nucleotide-binding protein subunit gamma | Gng12 | 7 | 0,0000 | 0,0000 | #DIV/0! | 0,0772 | 0,0920 | 119,1500 |
| MKIAA0291 protein (Fragment | mKIAA0291 | 110 | 0,0031 | 0,0035 | 115,7215 | 0,0000 | 0,0000 |  |
| Monocarboxylate transporter 1 | Slc16a1 | 53 | 0,0065 | 0,0094 | 144,7194 | 0,0000 | 0,0000 |  |
| Laminin, gamma 1 | Lamc1 | 177 | 0,0019 | 0,0028 | 144,7194 | 0,0000 | 0,0000 |  |
| Solute carrier family 35, member | Slc35e1 | 30 | 0,0053 | 0,0107 | 200,0000 | 0,0074 | 0,0148 | 200,0000 |
| Thioredoxin | Txn | 12 | 0,0283 | 0,0233 | 82,4273 | 0,0000 | 0,0000 |  |
| DIP2 disco-interacting protein 2 homolog B (Drosophila | Dip2b | 147 | 0,0023 | 0,0019 | 82,4273 | 0,0000 | 0,0000 |  |
| Large proline-rich protein | Bag6 | 115 | 0,0030 | 0,0024 | 82,1774 | 0,0000 | 0,0000 |  |
| Eukaryotic translation initiation factor | Eif2a | 64 | 0,0052 | 0,0041 | 78,5634 | 0,0000 | 0,0000 |  |
| Neuropilin-1 | Nrp1 | 103 | 0,0026 | 0,0033 | 128,0524 | 0,0015 | 0,0031 | 200,0000 |
| Lymphocyte antigen 75 | Ly75 | 197 | 0,0017 | 0,0025 | 143,7033 | 0,0000 | 0,0000 |  |
| Anion exchange protein | Slc4a7 | 127 | 0,0028 | 0,0023 | 81,9888 | 0,0000 | 0,0000 |  |
| Eukaryotic translation initiation factor 3 subunit | Eif3f | 38 | 0,0069 | 0,0088 | 128,5592 | 0,0038 | 0,0075 | 200,0000 |
| receptor protein-tyrosine kinase | Ddr1 | 101 | 0,0018 | 0,0035 | 200,0000 | 0,0000 | 0,0000 |  |
| Splicing factor U2AF 65 kDa | U2af2 | 54 | 0,0047 | 0,0062 | 130,6420 | 0,0000 | 0,0000 |  |
| Nucleoprotein TPR | Tpr | 274 | 0,0009 | 0,0011 | 124,5174 | 0,0000 | 0,0000 |  |
| MKIAA0666 protein (Fragment | Daam1 | 125 | 0,0020 | 0,0025 | 124,5174 | 0,0000 | 0,0000 |  |
| N(G),N(G)-dimethylarginine | Ddah1 | 31 | 0,0084 | 0,0108 | 128,5592 | 0,0000 | 0,0000 |  |
| Nucleotide exchange factor SIL1 | Sil1 | 52 | 0,0051 | 0,0065 | 128,9627 | 0,0000 | 0,0000 |  |
| leucine--tRNA ligase (Fragment | Lars1 | 138 | 0,0019 | 0,0024 | 126,7742 | 0,0000 | 0,0000 |  |
| Large ribosomal subunit protein | Mrpl19 | 34 | 0,0052 | 0,0103 | 200,0000 | 0,0033 | 0,0065 | 200,0000 |
| NADH dehydrogenase [ubiquinone] 1 beta subcomplex | Ndufb3 | 12 | 0,0074 | 0,0148 | 200,0000 | 0,0185 | 0,0369 | 200,0000 |
| Adhesion G protein-coupled | Adgra3 | 145 | 0,0006 | 0,0011 | 200,0000 | 0,0015 | 0,0031 | 200,0000 |
| HnRNP-associated with lethal yellow | Raly | 23 | 0,0113 | 0,0143 | 126,3917 | 0,0000 | 0,0000 |  |
| Alpha-centractin | Actr1a | 43 | 0,0058 | 0,0072 | 124,8768 | 0,0000 | 0,0000 |  |
| ATP-dependent RNA helicase SUPV3L1, mitochondrial | Supv3l1 | 87 | 0,0018 | 0,0037 | 200,0000 | 0,0013 | 0,0025 | 200,0000 |
| 72 kDa type IV | Mmp2 | 74 | 0,0022 | 0,0043 | 200,0000 | 0,0015 | 0,0030 | 200,0000 |
| NSFL1 cofactor p47 (Fragment | Nsfl1c | 41 | 0,0060 | 0,0075 | 125,7098 | 0,0000 | 0,0000 |  |
| Serine palmitoyltransferase 2 | Sptlc2 | 63 | 0,0025 | 0,0051 | 200,0000 | 0,0000 | 0,0000 |  |
| ATP-binding cassette sub-family | Abcd3 | 75 | 0,0021 | 0,0043 | 200,0000 | 0,0000 | 0,0000 |  |
| BCL2-associated athanogene 3 | Bag3 | 62 | 0,0026 | 0,0052 | 200,0000 | 0,0000 | 0,0000 |  |
| Progranulin | Grn | 63 | 0,0028 | 0,0056 | 200,0000 | 0,0000 | 0,0000 |  |
| Cytochrome b-c1 complex subunit | Uqcrh | 10 | 0,0178 | 0,0356 | 200,0000 | 0,0000 | 0,0000 |  |
| Betaine--homocysteine S-methyltransferase | Bhmt | 45 | 0,0000 | 0,0000 | #DIV/0! | 0,0063 | 0,0127 | 200,0000 |
| Integrin alpha-5 | Itga5 | 115 | 0,0015 | 0,0031 | 200,0000 | 0,0000 | 0,0000 |  |
| ER membrane protein complex | Emc4 | 20 | 0,0088 | 0,0176 | 200,0000 | 0,0000 | 0,0000 |  |
| Cytosolic fatty-acid binding proteins domain-containing | Fabp5 | 14 | 0,0000 | 0,0000 | #DIV/0! | 0,0158 | 0,0317 | 200,0000 |
| Pescadillo homolog | Pes1 | 68 | 0,0026 | 0,0052 | 200,0000 | 0,0000 | 0,0000 |  |
| Acid phosphatase 2, lysosomal | Acp2 | 19 | 0,0084 | 0,0169 | 200,0000 | 0,0000 | 0,0000 |  |
