# Supplementary material for: Engineering of Glioblastoma‐Derived Biomimetic Vesicles and Their Structural and Molecular Features
Source: Adv Healthc Mater. 2026 May 8;15(23):e03775. doi: 10.1002/adhm.202503775 (PMC13280188; doi:10.1002/adhm.202503775)
Supplement: Supplementary file 3 — Supporting File 3: adhm71222‐sup‐0003‐TableS2.docx. [file ADHM-15-0-s003.docx]

**Supporting Table 2.**
Identification of the top 20 most abundant proteins in the BV by gene name and subcellular localization.

| **Gene name** | **Protein** | **MW(kDa)** | **Subcellular Localization** | **Mean RPA%** | **STD** |
| --- | --- | --- | --- | --- | --- |
| Actb | Cluster of Actin, beta | 42 | Cytoskeleton | 1,373 | 0,210 |
| Tubb2b | Cluster of Tubulin beta chain | 50 | Cytoskeleton | 1,240 | 0,127 |
| Rab1A | Cluster of Ras-related protein Rab-1A | 23 | Membrane | 1,171 | 0,089 |
| Mdh2 | Malate dehydrogenase | 36 | Mitochondria | 0,981 | 0,063 |
| Hspd1 | 60 kDa heat shock protein | 61 | Mitochondria | 0,930 | 0,055 |
| Atp5f1a | Cluster of ATP synthase subunit alpha | 60 | Mitochondria | 0,809 | 0,119 |
| Vdac1 | Voltage-dependent anion-selective channel protein 1 | 32 | Membrane | 0,770 | 0,099 |
| Hist2h4 | Histone H4 | 11 | Nucleus | 0,734 | 0,189 |
| Atp5f1b | ATP synthase subunit beta | 56 | Mitochondria | 0,700 | 0,081 |
| Hspa8 | Cluster of Heat shock cognate 71 kDa | 71 | Chaperone | 0,693 | 0,062 |
| Tuba1 | Cluster of Tubulin alpha-1B chain | 50 | Cytoskeleton | 0,683 | 0,078 |
| Hspa5 | 78 kDa glucose-regulated protein | 72 | Membrane | 0,650 | 0,060 |
| Atp5me | ATP synthase subunit e, mitochondrial | 8 | Mitochondria | 0,634 | 0,119 |
| Prss59 | Protease, serine 59 (Fragment) | 20 | Chaperone | 0,613 | 0,382 |
| Hsp90b1 | Endoplasmin | 92 | Chaperone | 0,596 | 0,043 |
| Slc25a5 | Cluster of ADP/ATP translocase 2 | 33 | Mitochondria | 0,595 | 0,062 |
| Rab2a | Cluster of Ras-related protein Rab-2A | 24 | Mitochondria | 0,565 | 0,036 |
| Got2 | Aspartate aminotransferase | 47 | Mitochondria | 0,554 | 0,043 |
| Rpl30 | Large ribosomal subunit protein eL30 | 13 | Cytosol | 0,546 | 0,049 |
| Krt6a | Cluster of Keratin, type II cytoskeletal 6A | 59 | Cytoskeleton | 0,530 | 0,185 |
