# Supplementary material for: Engineering of Glioblastoma‐Derived Biomimetic Vesicles and Their Structural and Molecular Features
Source: Adv Healthc Mater. 2026 May 8;15(23):e03775. doi: 10.1002/adhm.202503775 (PMC13280188; doi:10.1002/adhm.202503775)
Supplement: Supplementary file 4 — Supporting File 4: adhm71222‐sup‐0004‐TableS3.docx. [file ADHM-15-0-s002.docx]

**Supporting Table 3.**

KEGG pathways identified for the common proteins between the protein extract and BV.

| **KEGG Pathway ID** | **Pathway Description** | **Number of hits** |
| --- | --- | --- |
| mmu01100 | Metabolic pathways | 163 |
| mmu03010 | Ribosome | 79 |
| mmu05171 | Coronavirus disease - COVID-19 | 62 |
| mmu05022 | Pathways of neurodegeneration - multiple diseases | 60 |
| mmu05014 | Amyotrophic lateral sclerosis | 57 |
| mmu05010 | Alzheimer disease | 54 |
| mmu05016 | Huntington disease | 51 |
| mmu05020 | Prion disease | 48 |
| mmu05012 | Parkinson disease | 47 |
| mmu01200 | Carbon metabolism | 44 |
| mmu04141 | Protein processing in endoplasmic reticulum | 34 |
| mmu05415 | Diabetic cardiomyopathy | 34 |
| mmu04714 | Thermogenesis | 31 |
| mmu05208 | Chemical carcinogenesis - reactive oxygen species | 30 |
| mmu00190 | Oxidative phosphorylation | 28 |
| mmu05200 | Pathways in cancer | 27 |
| mmu05132 | Salmonella infection | 26 |
| mmu04145 | Phagosome | 25 |
| mmu05203 | Viral carcinogenesis | 25 |
| mmu05165 | Human papillomavirus infection | 25 |
| mmu04820 | Cytoskeleton in muscle cells | 24 |
| mmu04151 | PI3K-Akt signaling pathway | 24 |
| mmu05169 | Epstein-Barr virus infection | 23 |
| mmu03040 | Spliceosome | 23 |
| mmu04519 | Cadherin signaling | 23 |
| mmu04144 | Endocytosis | 22 |
| mmu00280 | Valine, leucine and isoleucine degradation | 21 |
| mmu05034 | Alcoholism | 21 |
| mmu01230 | Biosynthesis of amino acids | 21 |
| mmu05017 | Spinocerebellar ataxia | 21 |
| mmu04810 | Regulation of actin cytoskeleton | 21 |
| mmu04517 | IgSF CAM signaling | 21 |
| mmu04613 | Neutrophil extracellular trap formation | 21 |
| mmu04932 | Non-alcoholic fatty liver disease | 20 |
| mmu04510 | Focal adhesion | 20 |
| mmu04518 | Integrin signaling | 19 |
| mmu01212 | Fatty acid metabolism | 19 |
| mmu00020 | Citrate cycle | 17 |
| mmu00010 | Glycolysis / Gluconeogenesis | 17 |
| mmu04142 | Lysosome biogenesis | 17 |
| mmu04137 | Mitophagy - animal | 16 |
| mmu05170 | Human immunodeficiency virus 1 infection | 16 |
| mmu00071 | Fatty acid degradation | 15 |
| mmu05322 | Systemic lupus erythematosus | 15 |
| mmu05168 | Herpes simplex virus 1 infection | 15 |
| mmu05417 | Lipid and atherosclerosis | 15 |
| mmu05163 | Human cytomegalovirus infection | 15 |
| mmu03050 | Proteasome | 15 |
| mmu04382 | Cornified envelope formation | 14 |
| mmu04611 | Platelet activation | 14 |
| mmu05412 | Arrhythmogenic right ventricular cardiomyopathy | 13 |
| mmu00620 | Pyruvate metabolism | 13 |
| mmu05205 | Proteoglycans in cancer | 13 |
| mmu04066 | HIF-1 signaling pathway | 13 |
| mmu04915 | Estrogen signaling pathway | 13 |
| mmu01210 | 2-Oxocarboxylic acid metabolism | 12 |
| mmu00640 | Propanoate metabolism | 12 |
| mmu00330 | Arginine and proline metabolism | 12 |
| mmu05230 | Central carbon metabolism in cancer | 12 |
| mmu05160 | Hepatitis C | 12 |
| mmu01240 | Biosynthesis of cofactors | 12 |
| mmu00310 | Lysine degradation | 12 |
| mmu04015 | Rap1 signaling pathway | 12 |
| mmu04024 | cAMP signaling pathway | 11 |
| mmu04260 | Cardiac muscle contraction | 11 |
| mmu04530 | Tight junction | 11 |
| mmu04919 | Thyroid hormone signaling pathway | 11 |
| mmu04670 | Leukocyte transendothelial migration | 11 |
| mmu04071 | Sphingolipid signaling pathway | 11 |
| mmu04360 | Axon guidance | 11 |
| mmu04512 | ECM-receptor interaction | 11 |
| mmu00062 | Fatty acid elongation | 11 |
| mmu05166 | Human T-cell leukemia virus 1 infection | 11 |
| mmu05164 | Influenza A | 10 |
| mmu04723 | Retrograde endocannabinoid signaling | 10 |
| mmu04022 | cGMP-PKG signaling pathway | 10 |
| mmu05414 | Dilated cardiomyopathy | 10 |
| mmu05100 | Bacterial invasion of epithelial cells | 10 |
| mmu05162 | Measles | 10 |
| mmu00380 | Tryptophan metabolism | 10 |
| mmu00630 | Glyoxylate and dicarboxylate metabolism | 9 |
| mmu04014 | Ras signaling pathway | 9 |
| mmu04110 | Cell cycle | 9 |
| mmu05410 | Hypertrophic cardiomyopathy | 9 |
| mmu05152 | Tuberculosis | 9 |
| mmu04148 | Efferocytosis | 9 |
| mmu05207 | Chemical carcinogenesis - receptor activation | 9 |
| mmu04814 | Motor proteins | 9 |
| mmu04140 | Autophagy - animal | 9 |
| mmu01040 | Biosynthesis of unsaturated fatty acids | 9 |
| mmu04721 | Synaptic vesicle cycle | 9 |
| mmu03060 | Protein export | 9 |
| mmu04612 | Antigen processing and presentation | 9 |
| mmu03013 | Nucleocytoplasmic transport | 9 |
| mmu04146 | Peroxisome | 9 |
| mmu04520 | Adherens junction | 9 |
| mmu04261 | Adrenergic signaling in cardiomyocytes | 8 |
| mmu05206 | MicroRNAs in cancer | 8 |
| mmu00650 | Butanoate metabolism | 8 |
| mmu04540 | Gap junction | 8 |
| mmu00270 | Cysteine and methionine metabolism | 8 |
| mmu04918 | Thyroid hormone synthesis | 8 |
| mmu05135 | Yersinia infection | 8 |
| mmu05167 | Kaposi sarcoma-associated herpesvirus infection | 8 |
| mmu05146 | Amoebiasis | 8 |
| mmu04934 | Cushing syndrome | 8 |
| mmu04010 | MAPK signaling pathway | 8 |
| mmu04217 | Necroptosis | 8 |
| mmu04961 | Endocrine and other factor-regulated calcium reabsorption | 8 |
| mmu04922 | Glucagon signaling pathway | 8 |
| mmu04726 | Serotonergic synapse | 8 |
| mmu04910 | Insulin signaling pathway | 8 |
| mu04210 | Apoptosis | 8 |
| mu04972 | Pancreatic secretion | 8 |
| mu04152 | AMPK signaling pathway | 8 |
| mu04218 | Cellular senescence | 8 |
| mu05134 | Legionellosis | 8 |
| mu00510 | N-Glycan biosynthesis | 8 |
| mu00970 | Aminoacyl-tRNA biosynthesis | 7 |
| mu05222 | Small cell lung cancer | 7 |
| mu04964 | Proximal tubule bicarbonate reclamation | 7 |
| mu04974 | Protein digestion and absorption | 7 |
| mu05211 | Renal cell carcinoma | 7 |
| mu04213 | Longevity regulating pathway - multiple species | 7 |
| mu04926 | Relaxin signaling pathway | 7 |
| mmu04976 | Bile secretion | 7 |
| mmu04728 | Dopaminergic synapse | 7 |
| mmu04730 | Long-term depression | 7 |
| mmu04514 | Cell adhesion molecule | 7 |
| mmu04371 | Apelin signaling pathway | 7 |
| mmu03320 | PPAR signaling pathway | 7 |
| mmu04921 | Oxytocin signaling pathway | 7 |
| mmu00410 | beta-Alanine metabolism | 7 |
| mmu04933 | AGE-RAGE signaling pathway in diabetic complications | 7 |
| mmu04020 | Calcium signaling pathway | 7 |
| mmu04062 | Chemokine signaling pathway | 7 |
| mmu04970 | Salivary secretion | 6 |
| mmu04722 | Neurotrophin signaling pathway | 6 |
| mmu04979 | Cholesterol metabolism | 6 |
| mmu05161 | Hepatitis B | 6 |
| mmu05210 | Colorectal cancer | 6 |
| mmu05145 | Toxoplasmosis | 6 |
| mmu04072 | Phospholipase D signaling pathway | 6 |
| mmu04666 | Fc gamma R-mediated phagocytosis | 6 |
| mmu00785 | Lipoic acid metabolism | 6 |
| mmu04925 | Aldosterone synthesis and secretion | 6 |
| mmu04390 | Hippo signaling pathway | 6 |
| mmu04640 | Hematopoietic cell lineage | 6 |
| mmu04962 | Vasopressin-regulated water reabsorption | 6 |
| mmu04130 | SNARE interactions in vesicular transport | 6 |
| mmu00670 | One carbon pool by folate | 6 |
| mmu00480 | Glutathione metabolism | 6 |
| mmu05150 | Staphylococcus aureus infection | 6 |
| mmu03018 | RNA degradation | 6 |
| mmu05418 | Fluid shear stress and atherosclerosis | 6 |
| mmu03008 | Ribosome biogenesis in eukaryotes | 6 |
| mmu00030 | Pentose phosphate pathway | 6 |
| mmu00513 | Various types of N-glycan biosynthesis | 6 |
| mmu05215 | Prostate cancer | 6 |
| mmu03030 | DNA replication | 5 |
| mu04914 | Progesterone-mediated oocyte maturation | 5 |
| mmu04911 | Insulin secretion | 5 |
| mmu04935 | Growth hormone synthesis, secretion and action | 5 |
| mmu04971 | Gastric acid secretion | 5 |
| mmu04725 | Cholinergic synapse | 5 |
| mmu04916 | Melanogenesis | 5 |
| mmu00260 | Glycine, serine and threonine metabolism | 5 |
| mmu04068 | FoxO signaling pathway | 5 |
| mu04724 | Glutamatergic synapse | 5 |
| mu04936 | Alcoholic liver disease | 5 |
| mu04081 | Hormone signaling | 5 |
| mu04270 | Vascular smooth muscle contraction | 5 |
| mu00051 | Fructose and mannose metabolism | 5 |
| mu04931 | Insulin resistance | 5 |
| mu01521 | EGFR tyrosine kinase inhibitor resistance | 5 |
| mu04621 | NOD-like receptor signaling pathway | 5 |
| mu05226 | Gastric cancer | 5 |
| mu04150 | mTOR signaling pathway | 5 |
| mu00250 | Alanine, aspartate and glutamate metabolism | 5 |
| mu04082 | Neuroactive ligand signaling | 5 |
| mu04912 | GnRH signaling pathway | 5 |
| mu00600 | Sphingolipid metabolism | 5 |
| mu05142 | Chagas disease | 5 |
| mu04310 | Wnt signaling pathway | 5 |
| mu00100 | Steroid biosynthesis | 4 |
| mu04114 | Oocyte meiosis | 4 |
| mu03015 | mRNA surveillance pathway | 4 |
| mu05213 | Endometrial cancer | 4 |
| mmu04660 | T cell receptor signaling pathway | 4 |
| mmu05225 | Hepatocellular carcinoma | 4 |
| mmu04727 | GABAergic synapse | 4 |
| mmu04927 | Cortisol synthesis and secretion | 4 |
| mmu05212 | Pancreatic cancer | 4 |
| mmu04913 | Ovarian steroidogenesis | 4 |
| mmu05416 | Viral myocarditis | 4 |
| mmu01522 | Endocrine resistance | 4 |
| mmu05031 | Amphetamine addiction | 4 |
| mmu00230 | Purine metabolism | 4 |
| mmu04978 | Mineral absorption | 4 |
| mmu05224 | Breast cancer | 4 |
| mmu00860 | Porphyrin metabolism | 4 |
| mmu00220 | Arginine biosynthesis | 4 |
| mmu03273 | Virion - Lassa virus and SFTS virus | 4 |
| mmu04610 | Complement and coagulation cascades | 4 |
| mmu04657 | IL-17 signaling pathway | 4 |
| mmu04975 | Fat digestion and absorption | 4 |
| mmu03272 | Virion - Hepatitis viruses | 4 |
| mmu04623 | Cytosolic DNA-sensing pathway | 4 |
| mmu04720 | Long-term potentiation | 4 |
| mmu04928 | Parathyroid hormone synthesis, secretion and action | 4 |
| mmu04211 | Longevity regulating pathway | 4 |
| mmu00920 | Sulfur metabolism | 4 |
| mmu04650 | Natural killer cell mediated cytotoxicity | 4 |
| mmu03082 | ATP-dependent chromatin remodeling | 4 |
| mmu00561 | Glycerolipid metabolism | 4 |
| mmu04370 | VEGF signaling pathway | 4 |
| mmu03250 | Viral life cycle - HIV-1 | 4 |
| mmu04330 | Notch signaling pathway | 3 |
| mmu04929 | GnRH secretion | 3 |
| mmu02010 | ABC transporters | 3 |
| mmu05030 | Cocaine addiction | 3 |
| mmu05231 | Choline metabolism in cancer | 3 |
| mmu04216 | Ferroptosis | 3 |
| mmu05221 | Acute myeloid leukemia | 3 |
| mu05220 | Chronic myeloid leukemia | 3 |
| mu04012 | ErbB signaling pathway | 3 |
| mu00140 | Steroid hormone biosynthesis | 3 |
| mu04662 | B cell receptor signaling pathway | 3 |
| mu00565 | Ether lipid metabolism | 3 |
| mu05235 | PD-L1 expression and PD-1 checkpoint pathway in cancer | 3 |
| mu05323 | Rheumatoid arthritis | 3 |
| mu00562 | Inositol phosphate metabolism | 3 |
| mu04750 | Inflammatory mediator regulation of TRP channels | 3 |
| mu04966 | Collecting duct acid secretion | 3 |
| mu04550 | Signaling pathways regulating pluripotency of stem cells | 3 |
| mu04973 | Carbohydrate digestion and absorption | 3 |
| mu04960 | Aldosterone-regulated sodium reabsorption | 3 |
| mu04625 | C-type lectin receptor signaling pathway | 3 |
| mu05216 | Thyroid cancer | 3 |
| mu04070 | Phosphatidylinositol signaling system | 3 |
| mu04664 | Fc epsilon RI signaling pathway | 3 |
| mu00514 | Other types of O-glycan biosynthesis | 3 |
| mu05032 | Morphine addiction | 3 |
| mu05140 | Leishmaniasis | 3 |
| mu05223 | Non-small cell lung cancer | 3 |
| mu05133 | Pertussis | 3 |
| mu04713 | Circadian entrainment | 3 |
| mu04350 | TGF-beta signaling pathway | 3 |
| mmu00900 | Terpenoid backbone biosynthesis | 2 |
| mmu00340 | Histidine metabolism | 2 |
| mmu00120 | Primary bile acid biosynthesis | 2 |
| mmu04923 | Regulation of lipolysis in adipocytes | 2 |
| mmu05219 | Bladder cancer | 2 |
| mmu04630 | JAK-STAT signaling pathway | 2 |
| mmu01232 | Nucleotide metabolism | 2 |
| mmu05218 | Melanoma | 2 |
| mmu05202 | Transcriptional misregulation in cancer | 2 |
| mmu00592 | alpha-Linolenic acid metabolism | 2 |
| mmu04668 | TNF signaling pathway | 2 |
| mmu00053 | Ascorbate and aldarate metabolism | 2 |
| mmu00052 | Galactose metabolism | 2 |
| mmu00520 | Amino sugar and nucleotide sugar metabolism | 2 |
| mmu04380 | Osteoclast differentiation | 2 |
| mmu04115 | p53 signaling pathway | 2 |
| mmu04930 | Type II diabetes mellitus | 2 |
| mmu00541 | Biosynthesis of various nucleotide sugars | 2 |
| mmu04920 | Adipocytokine signaling pathway | 2 |
| mmu04080 | Neuroactive ligand-receptor interaction | 2 |
| mmu04917 | Prolactin signaling pathway | 2 |
| mmu04940 | Type I diabetes mellitus | 2 |
| mmu00350 | Tyrosine metabolism | 2 |
| mmu04064 | NF-kappa B signaling pathway | 2 |
| mmu04659 | Th17 cell differentiation | 2 |
| mmu01250 | Biosynthesis of nucleotide sugars | 2 |
| mmu04924 | Renin secretion | 2 |
| mmu04980 | Cobalamin transport and metabolism | 2 |
| mmu04122 | Sulfur relay system | 2 |
| mmu05214 | Glioma | 2 |
| mmu00061 | Fatty acid biosynthesis | 2 |
| mmu04622 | RIG-I-like receptor signaling pathway | 2 |
| mmu04614 | Renin-angiotensin system | 2 |
| mmu03271 | Virion - Rotavirus | 2 |
| mu00360 | Phenylalanine metabolism | 2 |
| mmu04981 | Folate transport and metabolism | 2 |
| mu00910 | Nitrogen metabolism | 1 |
| mmu04215 | Apoptosis - multiple species | 1 |
| mmu00730 | Thiamine metabolism | 1 |
| mmu04120 | Ubiquitin mediated proteolysis | 1 |
| mmu01523 | Antifolate resistance | 1 |
| mmu05330 | Allograft rejection | 1 |
| mmu04977 | Vitamin digestion and absorption | 1 |
| mmu04742 | Taste transduction | 1 |
| mmu04740 | Olfactory transduction | 1 |
| mmu00500 | Starch and sucrose metabolism | 1 |
| mmu04744 | Phototransduction | 1 |
| mmu00563 | Glycosylphosphatidylinositol | 1 |
| mmu01524 | Platinum drug resistance | 1 |
| mmu03083 | Polycomb repressive complex | 1 |
| mmu00591 | Linoleic acid metabolism | 1 |
| mmu05217 | Basal cell carcinoma | 1 |
| mmu00982 | Drug metabolism - cytochrome P450 | 1 |
| mmu04620 | Toll-like receptor signaling pathway | 1 |
| mmu00760 | Nicotinate and nicotinamide metabolism | 1 |
| mmu00040 | Pentose and glucuronate interconversions | 1 |
| mmu00430 | Taurine and hypotaurine metabolism | 1 |
| mmu00524 | Neomycin, kanamycin and gentamicin biosynthesis | 1 |
| mmu00130 | Ubiquinone and other terpenoid-quinone biosynthesis | 1 |
| mmu05320 | Autoimmune thyroid disease | 1 |
| mmu04672 | Intestinal immune network for IgA production | 1 |
| mmu00400 | Phenylalanine, tyrosine and tryptophan biosynthesis | 1 |
| mmu00770 | Pantothenate and CoA biosynthesis | 1 |
| mmu00590 | Arachidonic acid metabolism | 1 |
| mmu00470 | D-Amino acid metabolism | 1 |
| mmu00564 | Glycerophospholipid metabolism | 1 |
| mmu05332 | Graft-versus-host disease | 1 |
| mmu05340 | Primary immunodeficiency | 1 |
| mmu00830 | Retinol metabolism | 1 |
| mmu04658 | Th1 and Th2 cell differentiation | 1 |
